# A Polyphenol‐Rich Diet Increases the Gut Microbiota Metabolite Indole 3‐Propionic Acid in Older Adults with Preserved Kidney Function

**DOI:** 10.1002/mnfr.202100349

**Published:** 2022-04-06

**Authors:** Gregorio Peron, Tomás Meroño, Giorgio Gargari, Nicole Hidalgo‐Liberona, Antonio Miñarro, Esteban Vegas Lozano, Pol Castellano‐Escuder, Raúl González‐Domínguez, Cristian del Bo', Stefano Bernardi, Paul A. Kroon, Antonio Cherubini, Patrizia Riso, Simone Guglielmetti, Cristina Andrés‐Lacueva

**Affiliations:** ^1^ Biomarkers and Nutrimetabolomics Laboratory Department of Nutrition Food Sciences and Gastronomy Food Innovation Network (XIA) Nutrition and Food Safety Research Institute (INSA) Faculty of Pharmacy and Food Sciences University of Barcelona Barcelona 08028 Spain; ^2^ Centro de Investigación Biomédica en Red de Fragilidad y Envejecimiento Saludable (CIBERFES) Instituto de Salud Carlos III Madrid 28029 Spain; ^3^ Department of Food, Environmental and Nutritional Sciences (DeFENS) Università degli Studi di Milano Milan 20133 Italy; ^4^ Department of Genetics, Microbiology and Statistics University of Barcelona Barcelona 08028 Spain; ^5^ Quadram Institute Bioscience Norwich Research Park Norwich NR4 7UQ UK; ^6^ Geriatria, Accettazione Geriatrica e Centro di Ricerca per l'Invecchiamento IRCCS INRCA Ancona 60127 Italy

**Keywords:** aging, gut microbiota, indole 3‐propionic acid, polyphenols, tryptophan gut metabolites

## Abstract

**Scope:**

Dietary polyphenols can alter the gut microbiota (GM) and promote the production of bioactive metabolites. Several indoles result of GM metabolism of dietary tryptophan have been associated with intestinal barrier integrity. Our aim is to study the changes in GM‐derived indoles during a polyphenol‐rich (PR) diet intervention in older adults.

**Methods and Results:**

Randomized, controlled, crossover trial in adults ≥ 60 years living in a residential care facility during an 8‐week PR versus control diet (*n* = 51). Seven GM‐tryptophan metabolites are measured in serum, and metataxonomic analysis of GM is performed on fecal samples. Exploratory subgroup analyses are performed based on renal function (RF). The PR‐diet significantly increases serum indole 3‐propionic acid (IPA) in subjects with normal RF, but not in subjects with impaired RF. Other GM‐tryptophan metabolites are not affected. Comparison of baseline GM composition shows shifts in Bacteroidales order members as well as higher abundance of Clostridiales in participants with normal RF. During the trial, variations of IPA are associated with changes in C‐reactive protein (β = 0.32, *p* = 0.010) and GM, particularly with the Clostridiales (*r* = 0.35, *p* < 0.001) and Enterobacteriales (*r* = −0.15, *p* < 0.05) orders.

**Conclusion:**

A PR diet increases the serum concentration of IPA in older adults with normal RF. Our findings may be important when defining appropriate dietary interventions for older adults.

**Trial registration number**: ISRCTN10214981 (https://doi.org/10.1186/ISRCTN10214981).

## Graphical abstract

In a randomized, controlled, crossover trial involving 51 adults ≥ 60 y receiving a PR‐diet for 8 weeks we observed that the PR‐diet significantly increased serum indole 3‐propionic acid (IPA), and the effect was dependent on renal function. During the trial, variation of IPA in volunteers with normal renal function was associated with changes in C‐reactive protein and gut microbiota, particularly with the Clostridiales and Enterobacteriales orders.

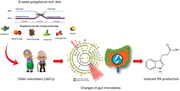

## 1 Introduction

Increasing evidence shows that alterations of the gut microbiota (GM) are associated with chronic diseases such as diabetes, cardiovascular disease, and cancer.^[^ ^1^ ^]^ GM is involved in the metabolism of dietary components such as amino acids, fibers, vitamins, and polyphenols, generating bioactive compounds with potential antioxidant and anti‐inflammatory activities, among others.^[^ ^2^, ^3^ ^]^ These GM‐derived metabolites could act in the intestinal lumen regulating local inflammatory process, mucosal immunity, and GM composition and functions, or could have a systemic effect after their diffusion to circulation.^[^ ^4^ ^]^ Among these metabolites, indoles derived from GM‐tryptophan metabolism have been related with extended lifespan in animal models ^[^ ^5^ ^]^ and healthy aging in humans.^[^ ^6^ ^]^ Of particular interest, indole 3‐propionic acid (IPA) has received increased attention during the last years for its anti‐inflammatory and antioxidant properties,^[^ ^7^ ^]^ as well as its association with gut barrier integrity ^[^ ^8^ ^]^ and cognitive performance.^[^ ^9^ ^]^ Moreover, IPA has been proposed as amarker of increased biodiversity of the intestinal microbiota.^[^ ^10^ ^]^ IPA is produced from the oxidative degradation of dietary tryptophan (Trp) by intestinal microorganisms, among which the only strains recognized so far belong to the bacterial species *Clostridium sporogenes*, *Peptostreptococcus anaerobius*, and *Clostridium cadaveris*.^[^ ^11^ ^]^ Circulating IPA levels have been positively correlated to microbiota α‐diversity and to butyrate‐producing gut bacteria such as *Faecalibacterium prausnitzii*,^[^ ^10^ ^]^ a species that is widely investigated for its beneficial effects on intestinal and systemic health.^[^ ^12^ ^]^ Moreover, an inverse correlation between circulating levels of IPA and arterial stiffness and components of metabolic syndrome, i.e., increased fasting glucose, insulin resistance, and visceral fat, has been reported,^[^ ^11^ ^]^ although the mechanism(s) of action involved are still unclear.

Among other bioactive GM‐derived indoles, indole 3‐acetic acid (IAA) and indolealdehyde have been shown to contribute to the maintenance of the intestinal barrier integrity and immune cell homeostasis through activation of the aryl hydrocarbon receptor (AhR),^[^ ^13^, ^14^ ^]^ while indole 3‐lactic acid (ILA), another activator of the AhR pathway produced by *Bifidobacterium longum* subsp. *infantis*, can significantly attenuate intestinal inflammatory processes through the inhibition of the NF‐kB pathway and the expression of IL‐8 in intestinal epithelial cells.^[^ ^15^ ^]^ However, compared to IPA, limited information about the effects exerted by these metabolites in vivo have been published.

Dietary polyphenols are natural compounds found in several foods and beverages, such as vegetables, fruits, green tea, and coffee, whose beneficial effects are widely investigated in literature.^[^ ^16^ ^]^ Due to their poor absorption, polyphenols accumulate in the intestine, where they can exert a local immunomodulatory activity and affect the GM community structure, leading to beneficial effects on intestinal permeability and host health.^[^ ^17^, ^18^ ^]^ This was recently reported in a study published by our group where, within the MaPLE trial, we showed that an 8‐week polyphenol‐rich (PR) diet led to a higher abundance of butyrate‐producing bacteria such as *F. prausnitzii* and *Butyricicoccus* spp. in older adults.^[^ ^19^ ^]^ Polyphenols can also affect the metabolism of other nutrients such as Trp through the modulation of GM composition, with consequent beneficial effects on health. Siddarth et al. reported the stabilization of performance of a memory score involving visual‐spatial learning in older subjects consuming 237 mL of an ellagitannin‐rich pomegranate juice (PJ) daily for 12 months ^[^ ^20^ ^]^ and, more recently, Yang et al. observed that plasma IPA levels were stable in volunteers undergoing PJ consumption, while in volunteers receiving the placebo, the amount of the same metabolite was decreasing.^[^ ^21^ ^]^ Alteration of plasma IPA levels were associated to PJ‐induced variations of some bacterial genera in the GM, among which Catenibacterium and Sutterrella.^[^ ^21^ ^]^ Although a direct correlation among the intake of PJ, plasma IPA levels and the effects on performance of memory score were not reported in these studies, the results by Siddarth and Yang are a preliminary confirmation that pomegranate polyphenols can alter the GM and interfere with the production of microbial Trp metabolites.

Considering the beneficial effects of IPA on gut microbiota and health in general, it is important to find reliable strategies to promote the production of this metabolite. Recently, a relationship between the consumption of dietary fiber and the production of IPA by the GM has been established,^[^ ^10^, ^22^ ^]^ but, in light of the preliminary literature data suggesting the same effect induced by pomegranate polyphenols, it is necessary to assess if the consumption of these widely diffused nutrients can lead to similar effects. Hence, in this posthoc analysis, we aimed at moving a step forward from the results by Yang et al. on pomegranate.^[^ ^21^ ^]^ Using a multitargeted metabolomics approach, we studied whether a diet with polyphenol‐rich foods (the PR‐diet), including ellagitannin‐rich pomegranate juice, administered in the MaPLE trial could positively affect the production of IPA and other six GM‐Trp metabolites (i.e., 3‐methylindole, indolealdehyde, indoleacetamide, ILA, IAA, and N‐acetyltryptophan) compared to a control diet. All these metabolites have been already reported to exert beneficial effects on host health, and are produced by specific bacteria in the human gut.^[^ ^5^, ^23^, ^24^, ^25^, ^26^, ^27^ ^]^ In this posthoc analysis, we also conducted an exploratory analysis to address the importance of impaired kidney function on the effects of the dietary intervention, considering that an association between renal function and IPA levels has been recently described.^[^ ^28^ ^]^

## 2 Results

### 2.1 Clinical Characteristics

**Table** **1** shows the clinical characteristics of the 51 subjects enrolled and stratified according to renal function. In total, 18 subjects had impaired renal function (IRF), 14 participants presented an estimated glomerular filtration rate (eGFR) < 60 mL min^−1^ 1.73 m^−2^ and four > 120 mL min^−1^ 1.73 m^−2^. These subjects with IRF were significantly older, mainly male, and showed a higher body mass index (BMI) than the other participants (Table 1). Significant differences between the participants with normal and impaired renal function were only noticed in glucose levels and eGFR after adjustment for age, sex, and BMI.

**Table 1:** General characteristics of the study population based on renal function

Variables	NRF (*n* = 33)	IRF (*n* = 18)	*p*‐value*	*p*‐value (ANCOVA)
Age [years]	75 (66‐80)	88 (80‐91)	**0.000**	–
Female sex (*n*, %)	18 (55%)	4 (22%)	**0.039**	–
BMI [kg m^−2^]	24.8 (21.2‐28.9)	28.8 (25.0‐32.3)	**0.027**	–
SBP [mmHg]	125 (120‐130)	126 (119‐130)	0.767	0.789
DBP [mmHg]	80 (73‐81)	73 (70‐76)	**0.010**	0.837
Glucose [mg dL^−1^]	97 (87‐119)	93 (83‐110)	0.183	**0.039**
Creatinine [mg dL^−1^]	0.90 (0.63‐ 1.02)	1.1 (0.8‐ 1.2)	0.636	0.100
eGFR [mL min^−1^]	73 (66‐ 83)	42.2 (36.7‐ 52.1)	**0.002**	**0.032**
Uric acid [mg dL^−1^]	5.2 (4.4‐ 6.6)	5.9 (4.3‐ 8.4)	0.882	0.463
IL‐6 [pg mL^−1^]	3.0 (1.5‐ 4.3)	4.6 (2.4‐ 7.7)	**0.049**	0.958
TNF‐α [pg mL^−1^]	1.2 (0.9‐ 1.6)	1.6 (1.2‐ 2.3)	0.090	0.162
CRP [mg L^−1^]	3.0 (0.9‐ 7.3)	8.0 (2.2‐ 10.5)	0.055	0.521
VCAM‐1 [ng mL^−1^]	928 (581‐1243)	1179 (896‐1456)	0.090	0.967
ICAM‐1 [ng mL^−1^]	51.6 (42.1‐ 67.6)	46.7 (28.5‐ 54.9)	0.554	0.867

Data are reported as median + IQR.

NRF, normal renal function group; IRF, impaired renal function group; BMI, body mass index; SBP, systolic blood pressure; DBP, diastolic blood pressure; eGFR, estimated glomerular filtration rate; IL‐6, interleukin 6; TNF‐α, tumor necrosis factor α; CRP, C‐reactive protein; VCAM‐1, vascular cell adhesion molecule‐1; ICAM‐1, intercellular cell adhesion molecule‐1.

*Obtained by Mann‐Whitney *U* test or exact Fisher test for categorical variables. ANCOVA analysis was adjusted for age, sex, and BMI. Comparisons are considered statistically significant if *p*‐value ≤ 0.05.

### 2.2 Baseline Concentration of Trp Metabolites and Clinical Variables

**Figure 1:**
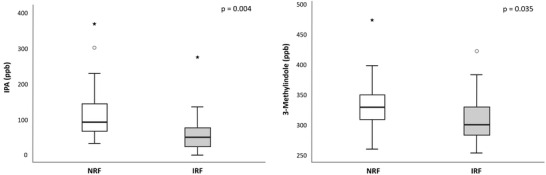
Indole 3‐propionic acid (IPA) and 3‐methylindole serum concentrations in subjects with normal renal function (NRF, *n* = 33) and in subjects with impaired renal function (IRF, *n* = 18). Reported *p*‐values are from Mann–Whitney *U* tests. In age‐, sex‐, and BMI‐adjusted comparisons only the difference in IPA was statistically significant, *p* = 0.013. *: outlier > 2 SD; °: outlier > 3 S.D.

Among the Trp metabolites analyzed at baseline, IPA and 3‐methylindole levels were significantly lower in subjects with IRF compared to subjects with normal renal function (NRF) (**Figure** **1**). When the comparison was adjusted for age, sex, and BMI, only the difference in IPA remained statistically significant (*p* = 0.013). Baseline correlations between IPA and renal function and inflammatory markers are shown in **Table** **2**. Briefly, IPA was inversely correlated with BMI, IL‐6, and CRP at baseline.

**Table 2:** Spearman correlation between IPA serum concentration and renal function and inflammatory markers at baseline

Variable	*r* (*p*‐value)
**BMI**	**−0.31 (0.029)**
SBP	−0.14 (0.924)
DBP	0.25 (0.078)
Glucose	0.11 (0.440)
Creatinine	−0.14 (0.337)
Uric acid	−0.21 (0.143)
GFR	0.11 (0.425)
**IL‐6**	**−0.37 (0.007)**
TNF‐α	−0.15 (0.284)
**CRP**	**−0.50 (<0.001)**
VCAM‐1	−0.13 (0.379)
ICAM‐1	0.05 (0.752)

Data with *p* ≤ 0.05 is significantly different. BMI, body mass index; SBP, systolic blood pressure; DBP, diastolic blood pressure; GFR, glomerular filtration rate; IL‐6, interleukin 6; TNF, tumor necrosis factor; CRP, C‐reactive protein; VCAM‐1, vascular cell adhesion molecule‐1; ICAM‐1, intercellular cell adhesion molecule‐1.

### 2.3 Changes of GM‐Trp Metabolites Following the PR Dietary Pattern

During the PR and control diets, subjects not only differed in their polyphenol intake,^[^ ^29^ ^]^ but also in the daily intake of animal and vegetable proteins and dietary fiber, especially among the participants with NRF (Supplementary Table S1, Supporting Information). No differences were observed between the NRF and IRF during the PR‐ or control‐ diets in comparisons adjusted by age, sex and BMI (Supplementary Table S1, Supporting Information). As Trp content of animal proteins is higher than vegetable proteins,^[^ ^30^ ^]^ the effect of diet on GM‐Trp metabolites was assessed adjusting by total energy, animal protein intake (as % of energy), as well as by total dietary fiber intake (g).

**Figure 2:**
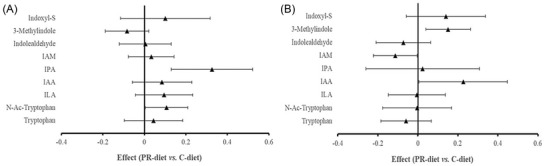
Effects of polyphenol rich‐diet (PR‐diet) compared to control diet (C‐diet) in gut microbiota‐derived Trp metabolites among participants with normal renal function (*n* = 33, Panel A) and with impaired renal function (*n* = 18, Panel B). The effects were estimated using linear mixed models for changes in the metabolites during treatment periods, adjusting for age, sex, BMI, baseline metabolite concentration, animal protein and dietary fiber intake, period and treatment × period interaction with subject specific random effects. IAM, indoleacetamide; IPA, indole 3‐propionic acid; IAA, indole 3‐acetic acid; ILA, indole 3‐lactic acid; N‐Ac‐tryptophan, N‐acetyl‐tryptophan.

In the whole cohort (*n* = 51), the PR‐diet induced a borderline significant increase of IPA and IAA (FDR‐adjusted *p*‐value: 0.10 < *p* < 0.05, Supplementary Figure S1, Supporting Information). Of note, in the subgroup of patients with NRF (*n* = 33), IPA levels were significantly increased (FDR‐adjusted *p*‐value = 0.026), while no effects were observed for the other metabolites (**Figure** **2**, Panel A). Conversely, in participants with IRF, there were no significant changes (all FDR‐adjusted *p*‐value > 0.05) (Figure 2, Panel B).

### 2.4 Association Between Changes in GM‐Trp Metabolites and Inflammatory Markers

Supplementary Figure S2 (Supporting Information) shows the effects of the PR‐diet in inflammatory markers among the NRF subjects. Only a trend toward a decrease of CRP levels was observed (β = −0.11, *p* = 0.069). During the trial, changes in IPA were significantly associated with changes in CRP (β = −0.32, *p* = 0.010) in fully adjusted models. Indeed, when baseline IPA and its changes during the trial periods were included in the linear mixed model for CRP, the treatment effect was reduced (β = −0.017, *p* = 0.807), suggesting baseline IPA and its changes as important predictors for the changes in CRP levels of older subjects with NRF, following a PR‐diet.

### 2.5 Association Between GM‐Trp Metabolites and GM Composition

**Figure 3:**
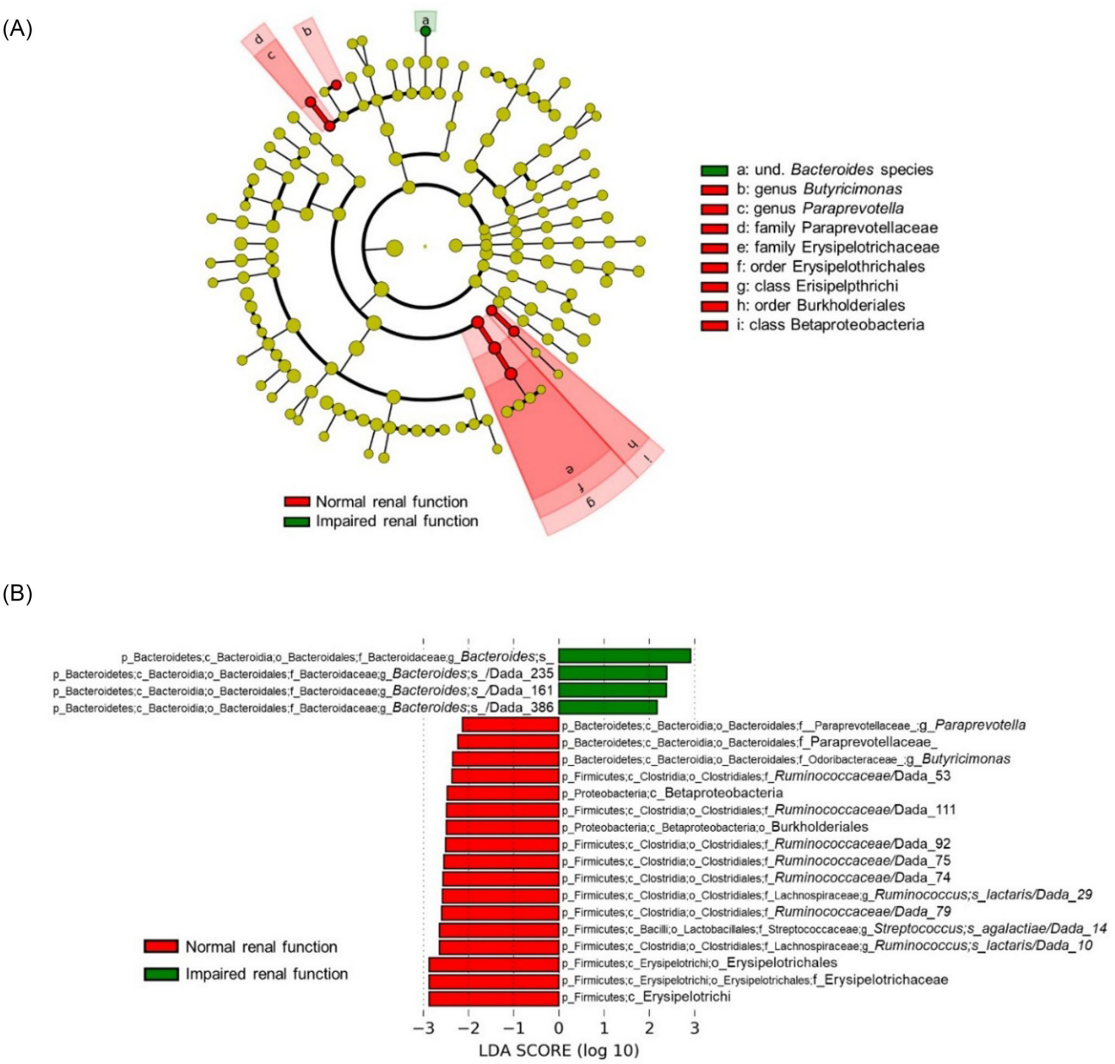
Linear discriminant analysis effect size (LEfSe) to identify significantly different bacterial taxa between older subjects with normal and impaired kidney function. LEfSe analysis was performed with the DESeq2‐normalized relative abundances on fecal bacterial taxa. A) Cladogram showing different abundant taxa with LDA score > 2.0 and *p* ≤ 0.05. (B) LDA scores of bacterial taxa with LDA > 2.0 and *p* < 0.1, including amplicon sequence variants (ASVs). “Dada_” indicates the specific ASV. The taxonomic lineage of each taxon is shown: p, phylum; c, class; o, order; f, family; g, genus; s, species. Und., undefined.

Subjects with IRF showed an altered GM composition at baseline characterized by altered abundance of members of the Bacteroidales order {higher *Bacteroides* spp. [three amplicon sequence variants (ASVs)] and lower abundance of *Paraprevotella* spp. and *Butyricimonas* spp.} and lower abundance of members belonging to the order Erysipelotrichales (one family), Clostridiales (six ASVs of the Ruminococcaceae family and two ASVs of the Lachnospiraceae family), and Burkholderiales (**Figure** **3**). Several of these differences are consistent with previous reports on subjects with various degrees of renal impairment.^[^ ^31^, ^32^, ^33^, ^34^ ^]^

**Figure 4:**
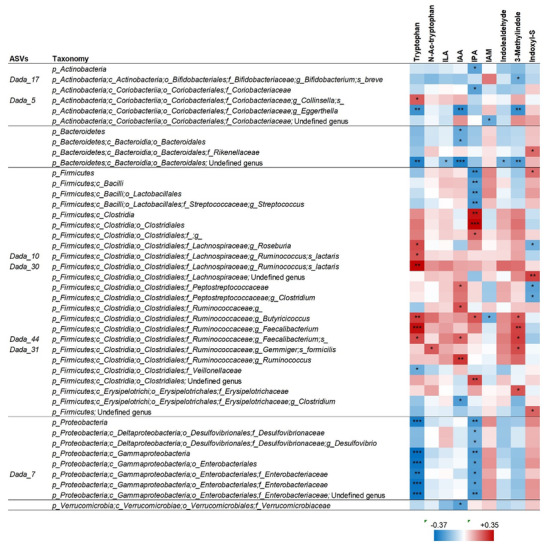
Correlations between the median abundances of tryptophan metabolites and fecal bacterial taxonomic units in the group of older subjects during the PR‐diet. This figure only includes taxa that significantly correlated with at least one tryptophan molecule according to Kendall's Tau rank correlation. The heatmap represents the R value of Spearman's correlation. Asterisks indicate the *p*‐values according to Kendall's Tau rank correlation: *p ≤ 0.05; **p ≤ 0.01; ***p ≤ 0.001. The taxonomic lineage of each taxon is shown: p, phylum; c, class; o, order; f, family; g, genus; s, species. ASVs, amplicon sequence variants. IAM, indoleacetamide; IPA, indole 3‐propionic acid; IAA, indole 3‐acetic acid; ILA, indole 3‐lactic acid; N‐Ac‐tryptophan, N‐acetyl‐tryptophan.

Changes in GM‐Trp metabolites were significantly correlated with some changes in GM composition during the intervention. **Figure** **4** shows the correlations of median data at the four time points for each volunteer between GM‐Trp metabolites and the bacterial relative abundance from the taxonomic level of phylum until ASVs. IPA levels were positively correlated with members of the order Clostridiales, and particularly within this family with the butyrate‐producing genus *Butyricicoccus*. Conversely, inverse correlations were observed with *Streptococcus* spp. and with several members of the Desulfovibrionaceae and Enterobacteriaceae families. Similar to median data correlations, these results were consistent across all the four time‐points of the intervention (Supplementary Figures S3–S7, Supporting Information).

## 3 Discussion

IPA is a metabolite generated by the microbial degradation (deamination) of dietary tryptophan in the gut. In humans, the production of IPA has only been demonstrated for *C. sporogenes* and few other bacterial species, including *Peptostreptooccus anaerobius* and *Clostridium cadaveris*.^[^ ^11^ ^]^ Notably, IPA was shown to inhibit gut dysbiosis ^[^ ^34^ ^]^ and exert antioxidant and anti‐inflammatory effects.^[^ ^35^ ^]^ Recently, Tuomainen et al. reported a positive correlation between the intake of dietary fibers and IPA serum levels, as a result of an unexplored diet‐induced modification of GM.^[^ ^22^ ^]^ Moreover, in the same study the authors reported a negative correlation with hsCRP.^[^ ^22^ ^]^ In our trial, IPA levels at baseline were negatively correlated with IL‐6 and CRP, which is an observation consistent with previous reports,^[^ ^22^, ^36^ ^]^ and during the trial, changes in IPA were associated with changes in CRP. Altogether, these results support the notion that this metabolite is involved in the regulation of inflammatory processes in older subjects. Furthermore, stratifying the study population into two groups according to renal function, we observed significantly different levels of IPA between the two groups, in agreement with a recent report.^[^ ^28^ ^]^

In the present study, the main findings were the significant increase of serum IPA after an 8‐week PR‐diet in subjects ≥ 60 years, and the differences in response between subjects with NRF versus subjects with IRF. To the best of our knowledge, this is the first report showing an association between the intake of a PR‐diet and increased concentrations of circulating IPA, with this being most likely a consequence of the modification of GM activity and/or composition. Indeed, the 16S rRNA gene profiling of fecal samples showed that bacterial taxa increased by the PR‐diet in the trial ^[^ ^19^ ^]^ were correlated with changes in IPA levels. Up to now, only the intake of dietary fiber ^[^ ^10^, ^22^ ^]^ and the prolonged consumption of pomegranate juice ^[^ ^21^ ^]^ have been demonstrated to increase the production of this metabolite by the GM, or to maintain constant its amounts in plasma. In our trial, we registered a small but significant increase of dietary fiber intake with the PR‐diet, however the increase of IPA remained significant after adjustment for dietary fiber intake.

There are several pieces of evidence that suggest that gut–kidney crosstalk plays a fundamental role in chronic kidney disease (CKD). On the one hand, CKD significantly modifies the composition and functions of the GM and contributes to dysbiosis in humans, while on the other hand, GM could lead to CKD onset and progression through the production of proinflammatory metabolites, such as the well known uremic toxin indoxyl‐sulfate.^[^ ^37^, ^38^ ^]^ In our study, the effects of the PR‐diet on the serum concentrations of IPA differed according to the presence of IRF. One explanation to this result could be the consequence of an altered GM composition at baseline in subjects with IRF. In fact, similarly to our findings, adults with CKD have been reported with increased abundance of several potentially proinflammatory bacteria such as Enterobacteriaceae, Streptococcaceae, and *Streptococcus*, and decreased abundances of Prevotellaceae, *Prevotella*, and *Roseburia*, among others.^[^ ^31^, ^32^, ^39^ ^]^ These results could be clinically relevant as lower circulating levels of IPA have been associated with rapid declines in renal function and with CKD.^[^ ^28^ ^]^ Indeed, alterations in the GM and its derived metabolites have been correlated with systemic inflammatory markers, albuminuria and with CKD severity.^[^ ^33^, ^40^ ^]^ Results of our trial support these findings and show that a PR‐diet might enhance IPA production in a manner partially related with changes in GM composition. Indeed, the PR‐diet in the whole MaPLE population led to a decrease in *Streptococcus* spp. and to an increase in the Clostridiales family Ruminococcaceae, and within this family the butyrate‐producing genus *Butyricicoccus*,^[^ ^19^ ^]^ all of which were correlated with IPA levels. Hence, the observed impact of IRF on IPA response might underline the critical role of an altered structure of the bacterial community in the gut, often associated to IRF and aging, in potentially hampering the effects of polyphenols on IPA production. Further studies should address if the GM composition in patients with IRF or CKD responds differently to a PR‐diet, and if the interactions between polyphenols and GM in these subjects can have a potential clinical relevance. In particular, we could not determine an increase of the specific *Clostridium* species directly involved in IPA production by the PR‐diet. In addition, we cannot assure which polyphenols could have promoted specific bacterial species. Further studies are required to assess the contribution of specific constituents of the MaPLE diet to the variations of microbiome and metabolome reported in this study, and food‐biomarker ontologies will represent valuable tools for this purpose.^[^ ^41^ ^]^

Overall, it appears that the PR‐dietary intervention led to changes in gut environment that allowed the thriving of beneficial bacteria, and reduced the growth of pathobionts such as *Streptococcus* spp. or *Proteobacteria*.^[^ ^11^, ^17^, ^42^ ^]^ Altogether, these changes were associated with the increase of circulating IPA levels in older adults, particularly among those with normal renal function.

## 4 Conclusion

A PR diet caused an increase in the serum concentration of the GM‐Trp metabolite IPA, and this effect was stronger in participants with preserved renal function and inversely correlated with serum CRP levels. Changes in IPA levels were correlated with PR‐diet‐induced changes in the GM, positively with members of the Clostridiales order and negatively with *Streptococcus* spp. and members of the Entereobacteriaceae family. Further studies will be required to confirm the influence of polyphenols on GM composition and IPA levels in older adults affected by chronic conditions, such as CKD.

### 4.1 Strengths and Limitations of the Study

The crossover design of this RCT is a clear strength of the study, as the same subject went through both treatments. Carry‐over effects were not evident in any Trp‐derived GM‐metabolite attesting for the adequacy of the 8‐week trial and washout‐periods. Furthermore, we adjusted the statistical analyses for clinical and diet covariates in an attempt to reduce residual confounding. Another strength of the study relies in the well‐controlled setting enabling the accurate evaluation of daily menus provided to the volunteers, and the use of weighed‐food records, which may be considered as the “gold standard” for dietary assessment. In the present study, from the borderline significant increase in IPA levels in the whole MaPLE population, we observed a near zero (β = 0.024, FDR‐adjusted *p*‐value = 0.97) effect in the IRF group. Furthermore, baseline differences in GM composition between the groups were consistent with previous studies in patients with different degrees of renal impairment, and abundances of specific bacterial taxa correlated with IPA levels, such as members of the family Ruminococcaceae from the Clostridiales order. However, the influence of renal function was results of an exploratory analysis and the present study was not specifically designed to address the differences between subjects with IRF and NRF. In fact, differences in age and sex between the subgroups were expected because of renal disease epidemiology. In particular, the age difference between the two subgroups was a relevant confounding factor. However, the studied population had a mean age of 78 ± 10 years, thus our results should be also considered in the framework of biological aging, which is correlated but different from chronological age. In this context, IRF may be considered as a proxy for biological aging mechanisms coping the functional reserve of multiple organ systems (including age‐related changes in GM, as explained previously) of the individual.^[^ ^43^ ^]^ Hence, whether these results were due to biological aging and/or IRF and/or alterations in GM composition itself cannot be established in the present study, and our results remain preliminary. The role of kidney function on the IPA‐increasing potential of polyphenols should be addressed on future trials specifically design for this purpose.

Finally, considering that the results here presented were obtained from a posthoc analysis of data acquired during a trial designed with other objectives, they should be validated in future studies specifically designed for evaluating the effects of dietary polyphenols on GM and IPA.

## 5 Experimental Section

### MaPLE Study Design and Participants

The full protocol of the MaPLE study has been reported in detail elsewhere^[^ ^44^ ^]^ and is described in the Supplementary Material. The MaPLE trial was an 8‐week, randomized, controlled, crossover study in which the effect of a PR‐diet versus a control diet was evaluated. The trial was carried out in a group of older subjects (≥ 60 y) with increased intestinal permeability recruited at Civitas Vitae (OIC Foundation, Padua, Italy), consisting of a mix of residential care and independent residences. The study protocol complied with the principles of the Declaration of Helsinki, and was approved by the Ethics Committee of the University of Milan, Italy (ref: 6/16/CE_15.02.16_Verbale_All‐7). All subjects and their parents were informed about the study protocol and signed an informed consent before the enrolment. The trial was registered under ISRCTN.com (ISRCTN10214981 https://doi.org/10.1186/ISRCTN10214981).

During the data analysis step, subjects were characterized for different functional capacity. In the present study, two groups of subjects were considered (with normal and impaired renal function, NRF and IRF, respectively) based on the eGFR, in order to evaluate the influence of kidney function on the response to the PR‐diet. NRF was defined by eGFR values between 60 and 120 mL min^−1^ 1.73 m^−2^ and IRF by eGFR values < 60 or > 120 mL min^−1^ 1.73 m^−2^, according to the international definition of reduced GFR^[^ ^45^ ^]^ and an age‐adjusted cut‐off value (> 97.5^th^ percentile) described in a pooled study of European Cohorts,^[^ ^46^ ^]^ respectively.

### Blood Sampling, Analysis, and Evaluation of Kidney Function Parameters

Briefly, after an overnight fast, blood samples were drawn in Vacutainer tubes containing silica gel for serum separation that was obtained by centrifugation (1400 X g at 4 °C for 15 min) and stored at −80 °C until analysis. Blood sampling and analysis of anthropometric, metabolic, and functional parameters were carried out as previously described.^[^ ^44^ ^]^ The eGFR was calculated using the Cockroft‐Gault formula.^[^ ^47^ ^]^ An eGFR < 15 mL min^−1^ 1.73 m^−2^ was considered as an exclusion criterion due to severe renal failure.^[^ ^44^ ^]^

### Sample Preparation and UPLC‐MS/MS Metabolomics Analysis of Serum

Serum samples were prepared for UPLC‐MS/MS analysis by a simple protein precipitation protocol, as already reported in a previously published work.^[^ ^48^ ^]^ Briefly, after thawing on ice, 100 µL of serum were added of 500 µL ACN containing 1.5 % v/v formic acid and 10 mM of ammonium formate. Samples were mixed and kept at −20 °C for 10 min, then centrifuged at 10 000 rpm and 4 °C for 10 min. After protein precipitation, 500 µL of supernatant were dried on vacuum and the residue was recovered with 100 µL of an 80/20 v/v mixture of water/ACN, containing 0.5 % v/v formic acid, 10 mM of ammonium formate, and 100 ppb of a mixture of internal standards. Finally, after centrifugation at 10 000 rpm and 4 °C for 5 min, samples were transferred to a 96‐well plate and analyzed using the semitargeted UPLC‐MS/MS method described in other works previously published by our group.^[^ ^48^, ^49^, ^50^ ^]^

The quality control of metabolomics data was performed using the POMA R/Bioconductor package (https://github.com/pcastellanoescuder/POMA). Data preprocessing included the removal of metabolites with more than 80 % missing values in all the study groups,^[^ ^51^ ^]^ the imputation of the remaining missing values using the KNN algorithm, and finally data normalization by means of *log* transformation and Pareto scaling. Afterwards, distances to the group centroid were computed based on Euclidean distances to remove outliers from the data matrix (± 1.5 × IQR). Finally, the coefficients of variation for areas, retention times, and peak widths of the internal standards added to samples were calculated for analytical reproducibility assessment.

### Dietary Assessment

During both PR and control intervention periods, weighed food records were used to estimate food, energy, nutrient, and polyphenol intake as previously reported.^[^ ^29^, ^44^ ^]^ Up to six detailed daily diaries reporting the information about the amounts of food consumed by each subject (calculated by the difference of the weight of food provided and the weight of leftovers) were analyzed. In addition, one diary was filled in by participants at baseline and scheduled the day of blood drawings and sampling according to what was previously reported.^[^ ^29^, ^44^ ^]^

### Metataxonomics of Fecal Samples

The bacterial community structure of fecal samples was assessed as described in the study by Del Bo' et al.^[^ ^19^ ^]^ In brief, DNA was isolated from faces resuspended in Lysing Matrix E bead beating tubes (MP Biomedicals, Santa Ana, CA, USA) through the FastDNA SPIN Kit for Soil (MP Biomedicals) according to the manufacturer's protocol. Then, the V3‐V4 region of the 16S rRNA gene was amplified and sequenced using an Illumina MiSeq sequencer (Illumina Inc, San Diego, CA, USA) using a 600 cycle MiSeq v3 reagent kit. Pairing, filtering, taxonomic assignment, and biodiversity analyses of sequencing reads were carried out by means of the bioinformatic pipeline Quantitative Insights Into Microbial Ecology (QIIME) 2 ^[^ ^52^ ^]^ through the Divisive Amplicon Denoising Algorithm (DADA2) using the Greengenes database (version 13_5).

### Statistical Analyses

Continuous variables are expressed as median (25^th^ and 75^th^ percentile) and categorical variables as *n*, percentage. Comparisons between the participants with normal and impaired renal function were analyzed by Mann–Whitney *U* test or the Fisher's Exact test. For adjusted comparisons, ANCOVA models, including age, sex, and BMI were used. In all these models, normality of the residuals was visually inspected through Q‐Q plots, and if major deviations were noticed, the dependent variable was log‐transformed and we repeated the analysis. Univariate comparisons of serum metabolite concentrations were done by Mann–Whitney *U* test and ANCOVA was used for adjusted comparisons, including age, sex, and BMI. Spearman's correlation coefficients were used to evaluate significant correlations between IPA serum concentration and renal function and inflammatory markers at baseline. For each trial period, the treatment effect on metabolomics data or inflammatory markers was estimated as the change between the “end of treatment – baseline” measurements. Afterwards, treatment effects on changes of metabolites or inflammatory markers concentrations were compared using a subject‐specific random effect linear mixed model using age, sex, BMI, baseline metabolite or inflammatory marker concentration, total energy (kcal), animal protein (as % of energy) and dietary fiber intake (g), period and treatment × period interaction as fixed factors or covariates. *p*‐values were adjusted for multiple comparisons using the Benjamini–Hochberg false discovery rate (FDR). A FDR‐adjusted *p*‐value < 0.05 was considered significant. The association of baseline IPA and IPA changes with CRP levels during the trial was assessed including them as covariates in the fully adjusted linear mixed model.

The relative abundances of bacterial taxa were used after data normalization carried out through the negative binomial distribution method [R/Bioconductor DESeq2 package ^[^ ^53^ ^]^]. Differently abundant taxa between NRF and IRF groups of volunteers were identified using linear discriminant analysis (LDA) combined with effect size (LEfSe) algorithm.^[^ ^54^ ^]^ A cut‐off value of LDA score (log_10_) above 2.0 was chosen. Correlation analyses (Spearman and Kendal tests) were carried out between the normalized relative abundance of bacterial taxa and other volunteers’ parameters using both median dada and data at each time point of the intervention trial, as previously described.^[^ ^55^, ^56^ ^]^

Finally, it should be noted that, in this study, a posthoc analysis of data acquired from the MaPLE trial ^[^ ^44^ ^]^ was performed. Hence, sample size calculations were based on the main outcomes of the study, thus they were not performed for the determinations included here.

All statistical analyses were performed using IBM SPSS Statistics 25 (IBM, USA) and R version 3.4.2 (R foundation, Austria).

## Conflict of Interest

The authors declare no conflict of interest.

## Author Contributions

G.P. and T.M. contributed equally to the work. Conceptualization, G.P., T.M., C.A.L., S.G., A.C., P.R., P.A.K.; Methodology, R.G.D., G.P., T.M., S.G., G.G., A.M., E.V.L., P.C.E., N.H.L., S.B., C.D.B.; Investigation, G.P., T.M., G.G., S.G.; Writing–Original Draft, G.P., T.M., G.G., S.G.; Writing–Review & Editing, G.P., T.M., G.G., N.H.L., A.M., E.L.V., P.C.E., R.G.D., C.D.B., S.B., P.A.K., A.C., P.R., S.G., C.A.L.; Funding acquisition, P.R., C.A.L., P.A.K.; Resources, C.A.L., S.G., P.R.; Supervision, C.A.L., S.G., P.R., A.C.

## Supporting information

Supporting Information

## Data Availability

Data available on request from the authors.
