# Supplementary material for: A Polyphenol‐Rich Diet Increases the Gut Microbiota Metabolite Indole 3‐Propionic Acid in Older Adults with Preserved Kidney Function
Source: Mol Nutr Food Res. 2022 Apr 6;66(21):2100349. doi: 10.1002/mnfr.202100349 (PMC9787513; doi:10.1002/mnfr.202100349)
Supplement: Supplementary file 1 — Supporting Information [file MNFR-66-2100349-s001.pdf]

SUPPLEMENTARY MATERIAL

Supplementary Materials and Methods

MaPLE trial

The trial was carried out at Civitas Vitae (OIC Foundation, Padua, Italy) in residential care and independent residences for older subjects, as previously described [1]. Inclusion criteria were: ≥ 60 years old and self-sufficient (Barthel index – activities of daily living, score ≥ 60), with an adequate nutritional status, evaluated with the Mini Nutritional Assessment test (score ≥ 24), and a good cognitive status (Mini Mental State Examination score ≥ 24). Exclusion criteria included: celiac disease and other severe disorders such as liver cirrhosis, renal insufficiency (dialysis) and severe Chronic Obstructive Pulmonary Disease (COPD; oxygen therapy for many hours a day) or severe cardiovascular disease (heart failure class III or IV NYHA - New York Heart Association). Subjects with malignant tumor that required treatment in the previous 2 years and those under antibiotic treatment in the last month before the trial were excluded.

The dietary intervention protocol has been already reported [1]. Briefly, the polyphenol-rich (PR) dietary pattern consisted in substituting some low-polyphenol products in the control diet with other comparable PR-products (e.g. foods used for snack or breakfast), while maintaining as much as possible the overall energy and nutrient composition. Specifically, subjects consumed 3 portions/day of selected PR-food such as berries and related products, blood orange and juice, pomegranate juice, green tea, Renetta apple and purée, and dark chocolate (callets and cocoa powder-based drink), providing a mean of 724 mg/day of total polyphenols as estimated by Folin-Ciocalteu analysis.

The study protocol complied with the principles of the Declaration of Helsinki, and was approved by the Ethics Committee of the University of Milan, Italy (ref: 6/16/CE_15.02.16_Verbale_All-7). All subjects and their parents were informed about the study protocol and signed an informed consent before the enrolment. The trial was registered under ISRCTN.com (ISRCTN10214981 https://doi.org/10.1186/ISRCTN10214981).

References

[1] S. Guglielmetti, S. Bernardi, C. Del Bo', A. Cherubini, M. Porrini, G. Gargari, N. Hidalgo-Liberona, R. Gonzalez-Dominguez, G. Peron, R. Zamora-Ros, M. S. Winterbone, B. Kirkup, P. A. Kroon, C. Andres-Lacueva, P. Riso, *BMC Geriatr.* **2020**, DOI 10.1186/s12877-020-1472-9.

Supplementary Tables

Supplementary Table 1. Intake of macronutrients of participants with NRF and IRF during the PR-diet and C-diet.

Nutrients	PR-diet				C-diet				PR *vs.* C-diet†	
	**NRF (n=33)**	**IRF (n=18)**	**p-value***	**p-value+**	**NRF (n=33)**	**IRF (n=18)**	**p-value***	**p-value+**	**NRF**	**IRF**
Energy (Kcal)	1530 (1403-1641)	1555 (1452-1643)	0.554	0.236	1541 (1457-1660)	1586 (1450-1679)	0.436	0.953	0.598	0.215
Carbohydrates (% of E)	48 (44-51)	46 (43-49)	0.180	0.350	44 (41-47)	45 (43-49)	0.134	0.180	**0.003**	0.811
Total Protein (% of E)	18.3 (16.2-19.0)	17.4 (16.8-18.7)	0.561	0.134	18.2 (16.9-19.0)	18.6 (16.4-19.3)	0.828	0.748	0.357	0.396
Animal Protein (% of E)	12.4 (11.0-12.8)	12.3 (11.9-13.2)	**0.042**	0.296	12.8 (11.9- 13.3)	13.3 (11.7-13.8)	0.767	0.282	**0.009**	0.711
Vegetable Protein (% of E)	5.9 (5.3-6.2)	4.9 (5.1-5.5)	**0.042**	0.561	5.4 (5.0-5.7)	5.2 (4.6-5.4)	0.419	0.385	0.514	**0.018**
Lipids (% of E)	35 (32-37)	36 (33-39)	0.270	0.262	38 (34-40)	37 (32-38)	0.168	0.166	**0.004**	0.811
SFA (% of E)	11.2 (9.7- 12.2)	12.1 (10.7- 13.9)	0.098	0.104	11.6 (9.6- 13.9)	11.6 (10.4- 13.4)	0.693	0.436	**0.014**	0.744
MUFA (% of E)	14.8 (12.9- 17.7)	16.3 (14.3- 17.3)	0.466	0.566	17.0 (15.0- 18.2)	16.2 (14.2- 17.8)	0.528	0.238	**0.014**	0.744
PUFA (% of E)	3.1 (2.5- 3.6)	3.2 (2.8- 3.6)	0.430	0.287	3.9 (3.1- 4.7)	3.3 (2.9- 3.4)	**0.016**	0.069	**<0.001**	0.811
Total Fibre (g)	18 (15- 20)	18 (15- 20)	0.813	0.900	17 (14- 18)	16 (14- 19)	0.581	0.903	**0.033**	0.058
Total Polyphenols (mg)	1342 (1262- 1476)	1416 (1266- 1679)	0.245	0.625	809 (699- 958)	788 (649- 974)	0.906	0.827	**<0.001**	**<0.001**

E, energy; NRF, normal renal function; IRF, impaired renal function; PR, polyphenol rich; C, control; SFA, saturated fatty acids; MUFA, monounsaturated fatty acids; PUFA, polyunsaturated fatty acids. *Mann-whitney U test; +ANCOVA adjusted for age, sex and BMI; †Wilcoxon signed-rank test.

Supplementary Figures

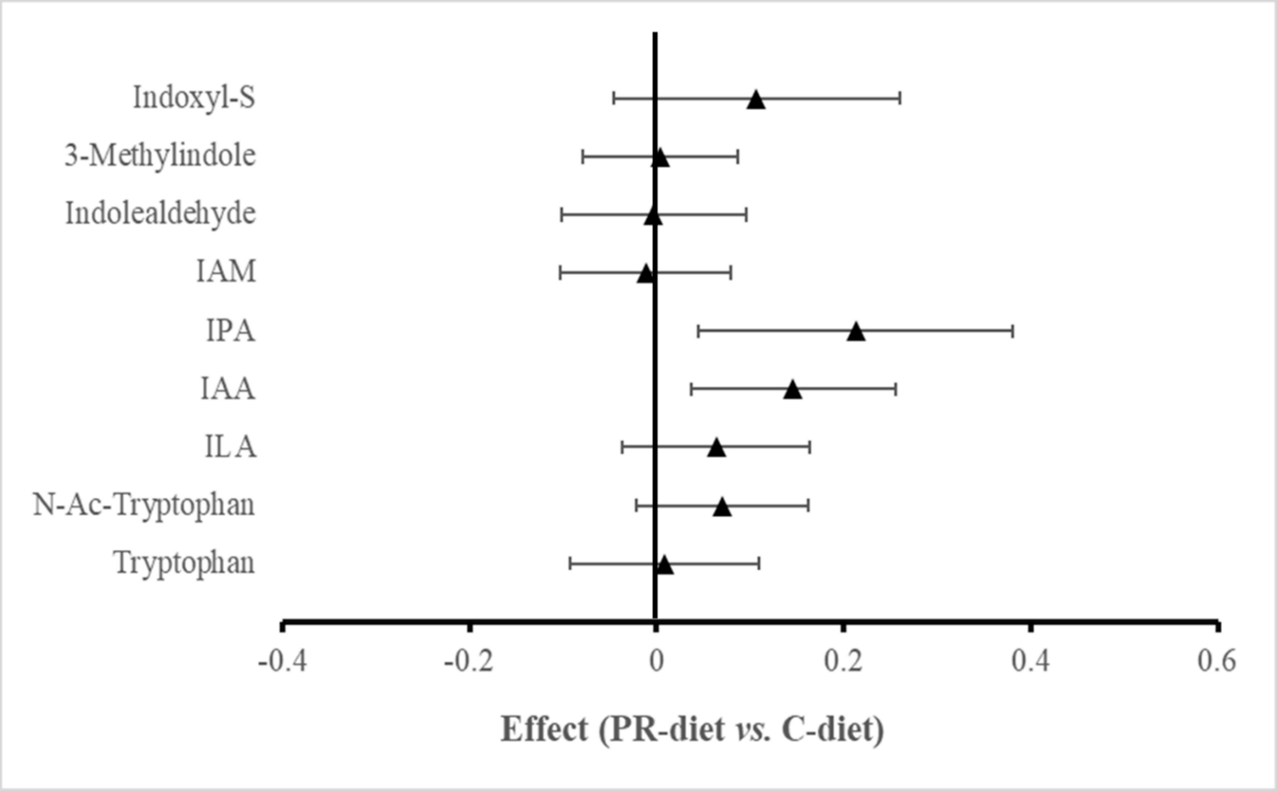

Supplementary Figure 1. Effects of polyphenol rich-diet (PR-diet) compared to control diet (C-diet) in gut microbiota-derived Trp metabolites among MaPLE participants (n=51). The effects were estimated using linear mixed models for changes in the metabolites during treatment periods, adjusting for age, sex, BMI, baseline metabolite concentration, total energy, animal protein and dietary fibre intake, period and treatment × period interaction with subject specific random effects. IAM, indoleacetamide; IPA, indole 3-propionic acid; IAA, indole 3-acetic acid; ILA, indole 3-lactic acid; N-Ac-tryptophan, N-acetyl-tryptophan.

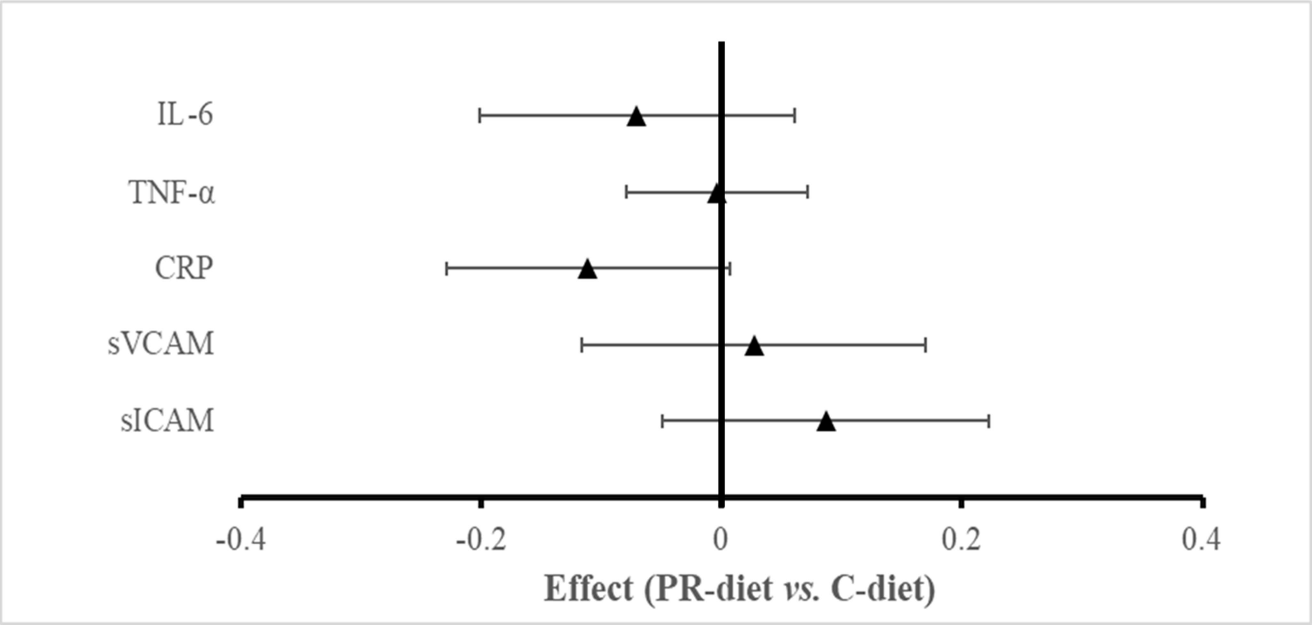

Supplementary Figure 2. Effects of polyphenol rich-diet (PR-diet) compared to control diet (C-diet) on inflammatory markers among participants with normal renal function (n=33). IL, interleukin; TNF, tumor necrosis factor; CRP, C-reactive protein; sVCAM, soluble vascular cell adhesion molecule; sICAM, soluble intercellular adhesion molecule. The effects were estimated using linear mixed models for changes in the inflammatory markers during treatment periods, adjusting for age, sex, BMI, baseline marker concentration, total energy, animal protein and dietary fibre intake, period and treatment × period interaction with subject specific random effects.

Supplementary Figures 3-7

The Figures here reported show correlations between the abundances of tryptophan metabolites and faecal bacterial taxonomic units in the group of older subjects at four time-points (T0, T1, T2 and T3) during the study. For each time-point, a heatmap showing the Spearman's rho for each correlation is reported, as well as a matrix showing merged Spearman's rho and p-values for each correlation. The taxonomic lineage of each taxon is shown: p, phylum; c, class; o, order; f, family; g, genus; s, species. ASVs, amplicon sequence variants. Tryptophan metabolites reported in columns are (from left column to right column): tryptophan, N-acetyltryptophan, indolelactic acid, indole 3-acetic acid, indole 3-propionic acid, indoleacetamide, indolealdehyde, 3-methylindole, and indoxyl sulphate. For color interpretation of the heatmaps, refer to Figure 4 in the manuscript. In the merged Spearman's rho and p-values matrices, red color indicates a significant ($p<0.05$) negative correlation, while green color a significant ($p<0.05$) positive correlation, according to Kendall's Tau rank correlation

Supplementary Figure 3A: Time-point: T0_*Heatmap*

	Spearman's rho								
	tryptophan	n_acetyltrypt	indolelactic	indoleacetic	indolepropio	indoleacetam	indolealdehy	x3_methylind	indoxyl_s
k__Bacteria.p__Actinobacteria	-0.08235294	0.03923107	-0.01803922	0.1545098	-0.02981562	0.00707549	-0.00470773	0.09654634	-0.0854902
k__Bacteria.p__Firmicutes	-0.1372549	-0.03609259	0.04627451	-0.02901961	-0.11455474	0.07468574	-0.00470773	-0.09968606	0.16078431
k__Bacteria.p__Actinobacteria.c__Coriobacteriia	-0.11058824	-0.01412319	-0.03058824	0.21411765	-0.08316988	0.00707549	-0.0439388	0.16875986	0.0054902
k__Bacteria.p__Firmicutes.c__Clostridia	0.01785457	0.07910125	-0.02635674	-0.16069111	0.08505511	-0.02301008	-0.03402204	-0.05871107	0.00935239
k__Bacteria.p__Actinobacteria.c__Coriobacteriia.o__Coriobacteriales	-0.11058824	-0.01412319	-0.03058824	0.21411765	-0.08316988	0.00707549	-0.0439388	0.16875986	0.0054902
k__Bacteria.p__Firmicutes.c__Clostridia.o__Clostridiales	0.09947545	0.03147039	0.01105283	-0.14708763	0.06123968	-0.02301008	-0.02381543	-0.01786859	-0.10967806
k__Bacteria.p__Actinobacteria.c__Coriobacteriia.o__Coriobacteriales.f__Coriobacteriaceae	-0.11058824	-0.01412319	-0.03058824	0.21411765	-0.08316988	0.00707549	-0.0439388	0.16875986	0.0054902
k__Bacteria.p__Firmicutes.c__Clostridia.o__Clostridiales.f__Lachnospiraceae	0.06980392	-0.06904669	-0.04156863	-0.16705882	-0.07375442	0.11713869	-0.02667713	-0.0133438	-0.03372549
k__Bacteria.p__Firmicutes.c__Clostridia.o__Clostridiales.f__Ruminococcaceae	-0.09490196	-0.00156924	-0.02745098	0.06980392	-0.10513928	-0.07311341	-0.07375442	-0.09968606	0.10901961
k__Bacteria.p__Firmicutes.c__Clostridia.o__Clostridiales.f__Lachnospiraceae.Other	0.06980392	0.15378581	0.06509804	0.08705882	0.02981562	-0.20518925	0.02353864	0.05729986	0.17333333
k__Bacteria.p__Firmicutes.c__Clostridia.o__Clostridiales.f__Lachnospiraceae.g__Ruminococcus	0.02901961	0.02667713	0.03372549	-0.10745098	-0.02353864	0.1926106	0.04236956	0.02433282	-0.04941176
k__Bacteria.p__Firmicutes.c__Clostridia.o__Clostridiales.f__Ruminococcaceae.Other	-0.01803922	-0.2557866	-0.1545098	0.04	0.04080032	-0.00707549	-0.12397019	0.00235479	0.09176471
k__Bacteria.p__Firmicutes.c__Clostridia.o__Clostridiales.Other	0.07846215	0.12323391	0.07375442	-0.04080032	0.15463108	-0.03145897	0.00235479	0.03455045	0.03923107
k__Bacteria.p__Firmicutes.c__Clostridia.o__Clostridiales.Other.Other	0.07846215	0.12323391	0.07375442	-0.04080032	0.15463108	-0.03145897	0.00235479	0.03455045	0.03923107
k__Bacteria.p__Bacteroidetes	-0.04083238	-0.12961512	-0.05182571	-0.18060476	-0.19402991	0.03778043	-0.11861748	-0.135167	0.08951714
k__Bacteria.p__Bacteroidetes.c__Bacteroidia	-0.03769143	-0.14532605	-0.06753047	-0.18688666	-0.18146117	0.02203858	-0.13118621	-0.135167	0.09265809
k__Bacteria.p__Firmicutes.c__Bacilli	-0.06753047	-0.02749412	0.07852381	-0.01099333	-0.13747058	0.09759944	0.03377849	-0.08644401	0.01413429
k__Bacteria.p__Bacteroidetes.c__Bacteroidia.o__Bacteroidales	-0.03769143	-0.14532605	-0.06753047	-0.18688666	-0.18146117	0.02203858	-0.13118621	-0.135167	0.09265809
k__Bacteria.p__Proteobacteria	-0.06942922	0.02131051	0.12150114	0.00631175	-0.12391665	0.02372494	0.06866719	-0.13738831	0.12465701
k__Bacteria.p__Actinobacteria.c__Actinobacteria	-0.01104556	0.1365451	0.05364985	0.06469541	0.06708863	-0.05061321	0.05446018	-0.04421693	-0.09467621
k__Bacteria.p__Actinobacteria.c__Coriobacteriia.o__Coriobacteriales.f__Coriobacteriaceae.g__Eggerthella	-0.03629255	0.02288906	-0.04576017	-0.01420143	-0.06235296	0.08224647	0.10181687	-0.17686771	0.01735731
k__Bacteria.p__Firmicutes.c__Bacilli.o__Lactobacillales	-0.06801342	-0.05459032	0.07908537	0.01581707	-0.07199592	0.1426898	0.04984333	-0.08389632	0.07592195
k__Bacteria.p__Bacteroidetes.c__Bacteroidia.o__Bacteroidales.f__Bacteroidaceae	0.02688903	-0.11155412	0.02372561	-0.13760854	-0.14161836	-0.0301234	-0.03402005	-0.10289171	0.09173903
k__Bacteria.p__Bacteroidetes.c__Bacteroidia.o__Bacteroidales.f__Bacteroidaceae.g__Bacteroides	0.02688903	-0.11155412	0.02372561	-0.13760854	-0.14161836	-0.0301234	-0.03402005	-0.10289171	0.09173903
k__Bacteria.p__Firmicutes.Other	-0.15196467	-0.01751065	-0.14719091	0.09467956	-0.02547004	-0.01355759	-0.05412383	-0.04140507	0.23789233
k__Bacteria.p__Firmicutes.Other.Other	-0.15196467	-0.01751065	-0.14719091	0.09467956	-0.02547004	-0.01355759	-0.05412383	-0.04140507	0.23789233
k__Bacteria.p__Firmicutes.Other.Other.Other	-0.15196467	-0.01751065	-0.14719091	0.09467956	-0.02547004	-0.01355759	-0.05412383	-0.04140507	0.23789233
k__Bacteria.p__Firmicutes.c__Clostridia.o__Clostridiales.f__	0.16469469	0.00795939	0.13446088	0.01352565	0.20694407	-0.04864782	0.14008521	0.28266921	0.05808074
k__Bacteria.p__Firmicutes.Other.Other.Other.Other	-0.15196467	-0.01751065	-0.14719091	0.09467956	-0.02547004	-0.01355759	-0.05412383	-0.04140507	0.23789233
k__Bacteria.p__Firmicutes.c__Clostridia.o__Clostridiales.f__.g__	0.16469469	0.00795939	0.13446088	0.01352565	0.20694407	-0.04864782	0.14008521	0.28266921	0.05808074
k__Bacteria.p__Firmicutes.c__Clostridia.o__Clostridiales.f__Ruminococcaceae.g__Clostridium	0.00079563	0.14645272	0.07240201	0.08035828	-0.0636751	-0.14594346	-0.00318375	0.08997639	0.08354079
k__Bacteria.p__Actinobacteria.c__Actinobacteria.o__Bifidobacteriales	0.00319413	0.11423489	0.04312073	0.04950899	0.09026953	-0.04162172	0.04233881	-0.02717141	-0.10061504
k__Bacteria.p__Actinobacteria.c__Actinobacteria.o__Bifidobacteriales.f__Bifidobacteriaceae	0.00319413	0.11423489	0.04312073	0.04950899	0.09026953	-0.04162172	0.04233881	-0.02717141	-0.10061504
k__Bacteria.p__Bacteroidetes.c__Bacteroidia.o__Bacteroidales.f__Rikenellaceae	-0.07027082	-0.08547646	-0.06068844	-0.08304733	-0.07589031	0.00480251	-0.077488	-0.1366562	0.20761833
k__Bacteria.p__Firmicutes.c__Clostridia.o__Clostridiales.f__Peptostreptococcaceae	-0.04152367	0.10944182	0.01916477	0.14373577	0.15417716	-0.08484427	0.01517806	0.03676132	-0.03353835
k__Bacteria.p__Actinobacteria.c__Actinobacteria.o__Bifidobacteriales.f__Bifidobacteriaceae.g__Bifidobacterium	0.00319413	0.11423489	0.04312073	0.04950899	0.09026953	-0.04162172	0.04233881	-0.02717141	-0.10061504
k__Bacteria.p__Bacteroidetes.c__Bacteroidia.o__Bacteroidales.f__Rikenellaceae.g__Alistipes	-0.00638826	-0.11743027	-0.03832954	-0.11498862	-0.03115497	0.04642422	-0.03594804	-0.12067302	0.16928879
k__Bacteria.p__Firmicutes.c__Clostridia.o__Clostridiales.f__Lachnospiraceae.g__Dorea	-0.07186788	-0.14618871	-0.25553026	0.01277651	-0.05831571	0.00480251	-0.12382103	-0.09909573	-0.07825614
k__Bacteria.p__Firmicutes.c__Clostridia.o__Clostridiales.f__Peptostreptococcaceae.g__Clostridium	-0.03034422	0.12382103	0.03034422	0.15172109	0.16855638	-0.0864451	0.02955728	0.04794955	-0.02874715
k__Bacteria.p__Firmicutes.c__Erysipelotrichi	0.04490069	0.12111901	0.01763956	-0.05933306	0.10347253	-0.1205537	-0.01684437	0.15727582	0.16035961
k__Bacteria.p__Firmicutes.c__Erysipelotrichi.o__Erysipelotrichales	0.04490069	0.12111901	0.01763956	-0.05933306	0.10347253	-0.1205537	-0.01684437	0.15727582	0.16035961
k__Bacteria.p__Firmicutes.c__Erysipelotrichi.o__Erysipelotrichales.f__Erysipelotrichaceae	0.04490069	0.12111901	0.01763956	-0.05933306	0.10347253	-0.1205537	-0.01684437	0.15727582	0.16035961
k__Bacteria.p__Firmicutes.c__Clostridia.o__Clostridiales.f__Ruminococcaceae.g__	-0.02405394	0.18689225	0.07216182	0.06414384	0.06015845	-0.00321477	0.06015845	-0.10511802	0.02084675
k__Bacteria.p__Firmicutes.c__Clostridia.o__Clostridiales.f__Lachnospiraceae.g__Clostridium	0.02013593	-0.05156822	-0.01369243	-0.02496856	-0.04512219	0.00242202	0.07735232	0.07818877	-0.04268818
k__Bacteria.p__Firmicutes.c__Bacilli.o__Lactobacillales.f__Streptococcaceae	-0.11394548	-0.0366397	0.02441689	0.03418364	-0.08712107	0.12237272	-0.01872696	-0.02280699	0.05534495
k__Bacteria.p__Firmicutes.c__Bacilli.o__Lactobacillales.f__Streptococcaceae.g__Streptococcus	-0.11394548	-0.0366397	0.02441689	0.03418364	-0.08712107	0.12237272	-0.01872696	-0.02280699	0.05534495
k__Bacteria.p__Firmicutes.c__Clostridia.o__Clostridiales.f__Ruminococcaceae.g__Faecalibacterium	0.09278417	-0.07083676	0.00162779	-0.02441689	-0.03338284	-0.15011053	-0.02524068	-0.00977442	-0.05371715
Dada_3.k__Bacteria.p__Firmicutes.c__Clostridia.o__Clostridiales.f__Lachnospiraceae.g__Ruminococcus.s__lactaris	0.07663671	0.03627246	0.11948735	-0.02224937	0.00659499	0.0223019	0.09232989	-0.04865717	-0.05521139
k__Bacteria.p__Bacteroidetes.c__Bacteroidia.o__Bacteroidales.Other	-0.14585697	-0.15498231	-0.24144685	-0.04037848	0.00989249	-0.18915319	-0.19372789	-0.03298791	0.03048987
k__Bacteria.p__Bacteroidetes.c__Bacteroidia.o__Bacteroidales.Other.Other	-0.14585697	-0.15498231	-0.24144685	-0.04037848	0.00989249	-0.18915319	-0.19372789	-0.03298791	0.03048987
k__Bacteria.p__Actinobacteria.c__Coriobacteriia.o__Coriobacteriales.f__Coriobacteriaceae.Other	-0.21990187	-0.01162202	-0.04232074	0.22488078	0.00498086	-0.02578511	-0.1867824	0.18602527	0.13194112
Dada_6.k__Bacteria.p__Firmicutes.c__Clostridia.o__Clostridiales.f__Lachnospiraceae.g__Ruminococcus.s__lactaris	0.03511541	0.07444043	0.07859164	0.00334432	-0.12127935	0.0771011	0.05102097	-0.05355122	-0.03511541
k__Bacteria.p__Proteobacteria.c__Deltaproteobacteria	-0.07524731	0.00585487	0.14547814	0.0802638	-0.13131626	0.02430361	0.0267651	-0.07697987	0.10367407
k__Bacteria.p__Proteobacteria.c__Deltaproteobacteria.o__Desulfovibrionales	-0.07524731	0.00585487	0.14547814	0.0802638	-0.13131626	0.02430361	0.0267651	-0.07697987	0.10367407
k__Bacteria.p__Proteobacteria.c__Deltaproteobacteria.o__Desulfovibrionales.f__Desulfovibrionaceae	-0.07524731	0.00585487	0.14547814	0.0802638	-0.13131626	0.02430361	0.0267651	-0.07697987	0.10367407
k__Bacteria.p__Firmicutes.c__Clostridia.o__Clostridiales.f__Ruminococcaceae.g__Gemmiger	-0.12374002	0.18317364	0.05685352	0.04514839	0.11291526	-0.01676111	0.03680201	0.05438795	0.03678757
k__Bacteria.p__Proteobacteria.c__Deltaproteobacteria.o__Desulfovibrionales.f__Desulfovibrionaceae.g__Desulfovibrio	-0.07524731	0.00585487	0.14547814	0.0802638	-0.13131626	0.02430361	0.0267651	-0.07697987	0.10367407
Dada_17.k__Bacteria.p__Actinobacteria.c__Actinobacteria.o__Bifidobacteriales.f__Bifidobacteriaceae.g__Bifidobacterium.s__brev	-0.06742956	-0.0143344	-0.06405809	-0.05900087	0.13406885	0.05238131	0.0092752	-0.04892482	-0.00505722
k__Bacteria.p__Proteobacteria.c__Gammaproteobacteria	-0.11968748	-0.01770721	0.04382922	-0.04888643	-0.14587365	-0.02281121	0.02782561	-0.15014859	0.11463026
k__Bacteria.p__Actinobacteria.c__Coriobacteriia.o__Coriobacteriales.f__Coriobacteriaceae.g__Collinsella	0.05057217	0.02192321	0.01854313	0.12811617	-0.02108001	0.00337944	-0.0059024	0.29354893	-0.06742956
Dada_1.k__Bacteria.p__Actinobacteria.c__Coriobacteriia.o__Coriobacteriales.f__Coriobacteriaceae.g__Collinsella.s__	0.04336109	-0.02976929	0.00595152	0.16239154	-0.01530992	-0.01619228	-0.02041323	0.30376599	-0.07736979
Dada_2.k__Bacteria.p__Actinobacteria.c__Coriobacteriia.o__Coriobacteriales.f__Coriobacteriaceae.g__Collinsella.s__	0.03315848	0.07229684	0.0484624	0.07396892	-0.02721764	0.06903023	0.03912535	0.24165137	-0.03825979
k__Bacteria.p__Proteobacteria.c__Gammaproteobacteria.o__Enterobacteriales	-0.1164798	0.0034022	0.04676196	-0.04336109	-0.12588156	-0.02556675	0.04252756	-0.15741375	0.11818023
k__Bacteria.p__Proteobacteria.c__Gammaproteobacteria.o__Enterobacteriales.f__Enterobacteriaceae	-0.1164798	0.0034022	0.04676196	-0.04336109	-0.12588156	-0.02556675	0.04252756	-0.15741375	0.11818023
k__Bacteria.p__Firmicutes.c__Clostridia.o__Clostridiales.f__Lachnospiraceae.g__Roseburia	0.1215811	-0.10546834	0.00595152	-0.18619763	-0.02126378	0.02301008	-0.09356062	0.06211461	-0.10967806
k__Bacteria.p__Proteobacteria.c__Gammaproteobacteria.o__Enterobacteriales.f__Enterobacteriaceae.Other	-0.1164798	0.0034022	0.04676196	-0.04336109	-0.12588156	-0.02556675	0.04252756	-0.15741375	0.11818023
Dada_4.k__Bacteria.p__Actinobacteria.c__Coriobacteriia.o__Coriobacteriales.f__Coriobacteriaceae.g__Collinsella.s__	0.05578064	-0.03176451	0.0094398	0.14331333	-0.04635902	-0.00602133	-0.02575501	0.31519336	-0.11070311
Dada_5.k__Bacteria.p__Actinobacteria.c__Coriobacteriia.o__Coriobacteriales.f__Coriobacteriaceae.g__Collinsella.s__	0.09525616	0.01287751	0.05234798	0.11241944	0.01373601	0.01118247	0.006868	0.31776987	-0.12100107
Dada_8.k__Bacteria.p__Firmicutes.c__Clostridia.o__Clostridiales.f__Lachnospiraceae.g__Ruminococcus.s__lactaris	0.22569703	0.10130304	-0.00429082	-0.17077456	-0.06009503	-0.11440527	0.10817105	0.05067141	0.03518471
k__Bacteria.Other	-0.03346838	0.03262301	0.06092962	0.18793783	0.06696303	0.086019	-0.07984053	-0.02490629	0.13473169
k__Bacteria.Other.Other	-0.03346838	0.03262301	0.06092962	0.18793783	0.06696303	0.086019	-0.07984053	-0.02490629	0.13473169
k__Bacteria.Other.Other.Other	-0.03346838	0.03262301	0.06092962	0.18793783	0.06696303	0.086019	-0.07984053	-0.02490629	0.13473169
k__Bacteria.Other.Other.Other.Other	-0.03346838	0.03262301	0.06092962	0.18793783	0.06696303	0.086019	-0.07984053	-0.02490629	0.13473169
k__Bacteria.p__Firmicutes.c__Clostridia.o__Clostridiales.f__Veillonellaceae	-0.18107253	-0.12534105	-0.0094398	0.10040514	-0.04120802	0.0172038	-0.0094435	-0.02147094	0.00257449
k__Bacteria.Other.Other.Other.Other.Other	-0.03346838	0.03262301	0.06092962	0.18793783	0.06696303	0.086019	-0.07984053	-0.02490629	0.13473169
k__Bacteria.p__Firmicutes.c__Clostridia.o__Clostridiales.f__Ruminococcaceae.g__Butyricicoccus	0.25830725	0.15281306	0.08324187	0.0283194	0.10902955	-0.16515648	0.07640653	0.38733571	-0.0660786
Dada_10.k__Bacteria.p__Firmicutes.c__Clostridia.o__Clostridiales.f__Lachnospiraceae.g__Ruminococcus.s__lactaris	0.08147465	-0.07023443	-0.03640357	0.06587312	-0.01300638	0.06255347	-0.10318391	0.1804259	-0.04507108
Dada_15.k__Bacteria.p__Firmicutes.c__Clostridia.o__Clostridiales.f__Lachnospiraceae.g__Ruminococcus.s__lactaris	0.19241886	0.1257283	0.19068535	-0.03293656	-0.15520942	0.0017376	0.18295635	0.05204593	0.03640357
Dada_33.k__Bacteria.p__Firmicutes.c__Clostridia.o__Clostridiales.f__Lachnospiraceae.g__Ruminococcus.s__lactaris	0.16469382	0.14635458	0.07008248	-0.12614846	-0.04294236	-0.06849176	0.07098635	0.11046658	0.04905773
Dada_39.k__Bacteria.p__Actinobacteria.c__Coriobacteriia.o__Coriobacteriales.f__Coriobacteriaceae.g__Collinsella.s__	0.08234691	0.01928024	0.01927268	0.11738815	-0.01665112	-0.01229339	-0.01840387	0.34981083	-0.0876031
Dada_41.k__Bacteria.p__Actinobacteria.c__Actinobacteria.o__Bifidobacteriales.f__Bifidobacteriaceae.g__Bifidobacterium.s__brev	-0.01927268	0.04294236	-0.04029742	-0.08234691	0.03242586	0.1870352	0.00262912	-0.10520626	-0.10687578
Dada_61.k__Bacteria.p__Actinobacteria.c__Actinobacteria.o__Bifidobacteriales.f__Bifidobacteriaceae.g__Bifidobacterium.s__brev	-0.04341684	0.01418249	-0.08063128	-0.08063128	0.03013779	0.16697225	-0.04786591	-0.13035281	-0.1267063
k__Bacteria.p__Bacteroidetes.c__Bacteroidia.o__Bacteroidales.f__Porphyromonadaceae	-0.10721302	0.03368342	0.0381005	-0.01329087	-0.06913964	-0.04973641	0.09218619	-0.0478847	0.13379476
k__Bacteria.p__Firmicutes.c__Clostridia.o__Clostridiales.f__Clostridiaceae	-0.061138	0.11345993	0.06999858	0.21176787	0.12941523	-0.13411069	-0.05584356	0.07360056	-0.03278415
k__Bacteria.p__Firmicutes.c__Erysipelotrichi.o__Erysipelotrichales.f__Erysipelotrichaceae.g__Clostridium	0.0274678	0.03545623	0.03632838	-0.08771974	-0.11789196	0.03819046	0.04432029	-0.08512836	0.15860439
Dada_30.k__Bacteria.p__Firmicutes.c__Clostridia.o__Clostridiales.f__Lachnospiraceae.g__Ruminococcus.s__lactaris	0.14260663	0.01794498	0.0905866	0.05650451	0.20636727	-0.07102218	0.07357442	0.21273152	0.00269069
k__Bacteria.p__Firmicutes.c__Bacilli.o__Bacillales	0.11211213	-0.05562944	-0.04036037	-0.04932934	-0.10766988	-0.14114534	-0.08164966	0.0646273	-0.19103907
k__Bacteria.p__Firmicutes.c__Clostridia.o__Clostridiales.f__Clostridiaceae.g__Clostridium	-0.08879281	0.09869739	0.05112313	0.19283287	0.12561486	-0.12316504	-0.08164966	0.07270571	-0.04394796
Dada_40.k__Bacteria.p__Firmicutes.c__Clostridia.o__Clostridiales.f__Ruminococcaceae.g__Gemmiger.s__formicilis	-0.07087249	0.25724084	0.09994839	0.13084152	0.1336198	-0.01366151	0.04726687	0.08820551	-0.02362416
Dada_53.k__Bacteria.p__Firmicutes.c__Clostridia.o__Clostridiales.f__Ruminococcaceae	0.06723801	-0.04090402	0.0417966	0.07632422	0.11544024	-0.09016595	-0.05726563	0.10730155	0.11812082
Dada_66.k__Bacteria.p__Actinobacteria.c__Actinobacteria.o__Bifidobacteriales.f__Bifidobacteriaceae.g__Bifidobacterium.s__brev	-0.05270006	0.03181424	-0.06723801	-0.09086217	0.01090774	0.14663352	-0.03908606	-0.1291256	-0.11630358
k__Bacteria.p__Verrucomicrobia	-0.10176563	-0.04272198	-0.13265877	-0.14901396	-0.05453869	0.14936582	-0.07544519	-0.24006449	-0.01453795
k__Bacteria.p__Verrucomicrobia.c__Verrucomicrobiae	-0.10176563	-0.04272198	-0.13265877	-0.14901396	-0.05453869	0.14936582	-0.07544519	-0.24006449	-0.01453795
k__Bacteria.p__Verrucomicrobia.c__Verrucomicrobiae.o__Verrucomicrobiales	-0.10176563	-0.04272198	-0.13265877	-0.14901396	-0.05453869	0.14936582	-0.07544519	-0.24006449	-0.01453795
k__Bacteria.p__Verrucomicrobia.c__Verrucomicrobiae.o__Verrucomicrobiales.f__Verrucomicrobiaceae	-0.10176563	-0.04272198	-0.13265877	-0.14901396	-0.05453869	0.14936582	-0.07544519	-0.24006449	-0.01453795
k__Bacteria.p__Bacteroidetes.c__Bacteroidia.o__Bacteroidales.f__Porphyromonadaceae.g__Macellibacteroides	-0.06360352	0.03726811	0.01635519	0.05088282	-0.01727059	-0.05737833	0.08453498	-0.03637341	0.09449666
Dada_12.k__Bacteria.p__Firmicutes.c__Clostridia.o__Clostridiales.f__Ruminococcaceae.g__Gemmiger.s__formicilis	-0.05343637	0.13364333	0.00737053	-0.04053793	0.13733005	-0.06187397	0.05530069	0.07099709	0.00737053
Dada_31.k__Bacteria.p__Firmicutes.c__Clostridia.o__Clostridiales.f__Ruminococcaceae.g__Gemmiger.s__formicilis	-0.0829185	0.22396779	0.0829185	-0.02026897	0.14654683	0.00461746	0.12534823	0.06915301	0.03501003
k__Bacteria.p__Firmicutes.c__Bacilli.o__Lactobacillales.Other	-0.07923323	0.1308783	-0.0276395	-0.0718627	-0.05437901	0.08865525	-0.00276503	-0.27661203	0.1547812
k__Bacteria.p__Firmicutes.c__Bacilli.o__Lactobacillales.Other.Other	-0.07923323	0.1308783	-0.0276395	-0.0718627	-0.05437901	0.08865525	-0.00276503	-0.27661203	0.1547812

Supplementary Figure 3B: Time-point: T0_*Merged Spearman's rho and p-values matrix*

	Merged rho+P								
	tryptophan	n_acetyltrypt	indolelactic	indoleacetic	indolepropioı	indoleacetam	indolealdehy	x3_methylind	indoxyl_s
k__Bacteria.p__Actinobacteria	-0.39375238	0.68465153	-0.85180894	0.10958182	-0.75758458	0.94170999	-0.96113058	0.31774728	-0.37598352
k__Bacteria.p__Firmicutes	-0.1552043	-0.70867661	0.63178892	-0.76377916	-0.23566893	0.44021469	-0.96113058	-0.30226476	0.09590213
k__Bacteria.p__Actinobacteria.c__Coriobacteriia	-0.25211316	-0.88375986	-0.75142099	0.02659825	-0.38924753	0.94170999	-0.64921081	0.0807431	0.95466032
k__Bacteria.p__Firmicutes.c__Clostridia	0.86015536	0.43525367	-0.79480984	-0.1128329	0.40149156	-0.82075553	-0.73718448	-0.56265666	0.92647329
k__Bacteria.p__Actinobacteria.c__Coriobacteriia.o__Coriobacteriales	-0.25211316	-0.88375986	-0.75142099	0.02659825	-0.38924753	0.94170999	-0.64921081	0.0807431	0.95466032
k__Bacteria.p__Firmicutes.c__Clostridia.o__Clostridiales	0.32631951	0.75624514	0.91315365	-0.14668021	0.54580943	-0.82075553	-0.81427944	-0.86014707	-0.27915192
k__Bacteria.p__Actinobacteria.c__Coriobacteriia.o__Coriobacteriales.f__Coriobacteriaceae	-0.25211316	-0.88375986	-0.75142099	0.02659825	-0.38924753	0.94170999	-0.64921081	0.0807431	0.95466032
k__Bacteria.p__Firmicutes.c__Clostridia.o__Clostridiales.f__Lachnospiraceae	0.46975558	-0.47474614	-0.66684856	-0.08362474	-0.44515749	0.22606578	-0.78242189	-0.8901719	-0.72689782
k__Bacteria.p__Firmicutes.c__Clostridia.o__Clostridiales.f__Ruminococcaceae	-0.32571201	-0.98703897	-0.77619781	0.46975558	-0.27641318	-0.44990005	-0.44515749	-0.30226476	0.25890317
k__Bacteria.p__Firmicutes.c__Clostridia.o__Clostridiales.f__Lachnospiraceae.Other	0.46975558	0.11138338	0.50022007	0.36728776	0.75758458	-0.03396362	0.80748302	0.55320757	0.07265232
k__Bacteria.p__Firmicutes.c__Clostridia.o__Clostridiales.f__Lachnospiraceae.g__Ruminococcus	0.76377916	0.78242189	0.72689782	-0.26581886	-0.80748302	0.04653469	0.66094135	0.80119167	-0.60886148
k__Bacteria.p__Firmicutes.c__Clostridia.o__Clostridiales.f__Ruminococcaceae.Other	-0.85180894	-0.00809867	-0.10958182	0.67870365	0.67275577	-0.94170999	-0.19936889	0.98055888	0.3419605
k__Bacteria.p__Firmicutes.c__Clostridia.o__Clostridiales.Other	0.4166494	0.20221325	0.44515749	-0.67275577	0.10955841	-0.7451871	0.98055888	0.72078292	0.68465153
k__Bacteria.p__Firmicutes.c__Clostridia.o__Clostridiales.Other.Other	0.4166494	0.20221325	0.44515749	-0.67275577	0.10955841	-0.7451871	0.98055888	0.72078292	0.68465153
k__Bacteria.p__Bacteroidetes	-0.67272865	-0.18012474	-0.59186817	-0.06171546	-0.04480348	0.69651832	-0.21995694	-0.162329	0.35442308
k__Bacteria.p__Bacteroidetes.c__Bacteroidia	-0.69660066	-0.13288023	-0.48480539	-0.05319678	-0.06058398	0.82002379	-0.17490262	-0.162329	0.33779212
k__Bacteria.p__Firmicutes.c__Bacilli	-0.48480539	-0.77616429	0.4166084	-0.90945602	-0.15514075	0.31366397	0.72685747	-0.37153052	0.8837497
k__Bacteria.p__Bacteroidetes.c__Bacteroidia.o__Bacteroidales	-0.69660066	-0.13288023	-0.48480539	-0.05319678	-0.06058398	0.82002379	-0.17490262	-0.162329	0.33779212
k__Bacteria.p__Proteobacteria	-0.47434739	0.82625115	0.21057309	0.94814335	-0.20180629	0.80725896	0.47936963	-0.1571624	0.19896343
k__Bacteria.p__Actinobacteria.c__Actinobacteria	-0.90938256	0.15957466	0.5803817	0.50499915	0.48952995	-0.6027459	0.57481431	-0.64889397	-0.32927387
k__Bacteria.p__Actinobacteria.c__Coriobacteriia.o__Coriobacteriales.f__Coriobacteriaceae.g__Eggerthella	-0.70842561	0.81360939	-0.63726292	-0.88365565	-0.52069388	0.3976974	0.29427935	-0.06857692	0.85805087
k__Bacteria.p__Firmicutes.c__Bacilli.o__Lactobacillales	-0.48421846	-0.57461201	0.41598219	0.87076859	-0.45916134	0.1430422	0.60832498	-0.38853771	0.43487601
k__Bacteria.p__Bacteroidetes.c__Bacteroidia.o__Bacteroidales.f__Bacteroidaceae	0.78211806	-0.25140332	0.80721267	-0.15697002	-0.14537772	-0.75718159	-0.72650508	-0.29028175	0.34539592
k__Bacteria.p__Bacteroidetes.c__Bacteroidia.o__Bacteroidales.f__Bacteroidaceae.g__Bacteroides	0.78211806	-0.25140332	0.80721267	-0.15697002	-0.14537772	-0.75718159	-0.72650508	-0.29028175	0.34539592
k__Bacteria.p__Firmicutes.Other	-0.11969106	-0.85774992	-0.13176202	0.33230094	-0.79431499	-0.8898156	-0.57957067	-0.6718066	0.01485296
k__Bacteria.p__Firmicutes.Other.Other	-0.11969106	-0.85774992	-0.13176202	0.33230094	-0.79431499	-0.8898156	-0.57957067	-0.6718066	0.01485296
k__Bacteria.p__Firmicutes.Other.Other.Other	-0.11969106	-0.85774992	-0.13176202	0.33230094	-0.79431499	-0.8898156	-0.57957067	-0.6718066	0.01485296
k__Bacteria.p__Firmicutes.c__Clostridia.o__Clostridiales.f__	0.09171432	0.93506632	0.16856227	0.88984644	0.03415128	-0.61911479	0.15159701	0.00382358	0.55202467
k__Bacteria.p__Firmicutes.Other.Other.Other.Other	-0.11969106	-0.85774992	-0.13176202	0.33230094	-0.79431499	-0.8898156	-0.57957067	-0.6718066	0.01485296
k__Bacteria.p__Firmicutes.c__Clostridia.o__Clostridiales.f__.g__	0.09171432	0.93506632	0.16856227	0.88984644	0.03415128	-0.61911479	0.15159701	0.00382358	0.55202467
k__Bacteria.p__Firmicutes.c__Clostridia.o__Clostridiales.f__Ruminococcaceae.g__Clostridium	0.99349973	0.13384998	0.45846644	0.41059662	-0.5145439	-0.13587857	-0.97400239	0.35722421	0.39231201
k__Bacteria.p__Actinobacteria.c__Actinobacteria.o__Bifidobacteriales	0.97397472	0.24348009	0.65963362	0.6130909	0.3567126	-0.67140075	0.66554008	-0.78153541	-0.30411708
k__Bacteria.p__Actinobacteria.c__Actinobacteria.o__Bifidobacteriales.f__Bifidobacteriaceae	0.97397472	0.24348009	0.65963362	0.6130909	0.3567126	-0.67140075	0.66554008	-0.78153541	-0.30411708
k__Bacteria.p__Bacteroidetes.c__Bacteroidia.o__Bacteroidales.f__Rikenellaceae	-0.47292997	-0.38282104	-0.53535716	-0.39631852	-0.43843646	0.96095978	-0.42885732	-0.16309127	0.03396085
k__Bacteria.p__Firmicutes.c__Clostridia.o__Clostridiales.f__Peptostreptococcaceae	-0.6714876	0.26382532	0.84481226	0.14208696	0.11545488	-0.38716554	0.87684715	0.7075148	-0.7319384
k__Bacteria.p__Actinobacteria.c__Actinobacteria.o__Bifidobacteriales.f__Bifidobacteriaceae.g__Bifidobacterium	0.97397472	0.24348009	0.65963362	0.6130909	0.3567126	-0.67140075	0.66554008	-0.78153541	-0.30411708
k__Bacteria.p__Bacteroidetes.c__Bacteroidia.o__Bacteroidales.f__Rikenellaceae.g__Alistipes	-0.94797713	-0.23054465	-0.6954398	-0.24021449	-0.75041618	0.63608795	-0.71359838	-0.2180906	0.08379967
k__Bacteria.p__Firmicutes.c__Clostridia.o__Clostridiales.f__Lachnospiraceae.g__Dorea	-0.46292937	-0.13554737	-0.00905711	0.89617517	-0.55157583	0.96095978	-0.20615595	-0.31182618	-0.42412862
k__Bacteria.p__Firmicutes.c__Clostridia.o__Clostridiales.f__Peptostreptococcaceae.g__Clostridium	-0.75661835	0.20615595	0.75661835	0.12123211	0.08525954	-0.37827155	0.76282107	0.6245685	-0.76905386
k__Bacteria.p__Firmicutes.c__Erysipelotrichi	0.64742688	0.21749248	0.85741229	-0.54561643	0.29208975	-0.22043965	-0.86382444	0.10942317	0.10239411
k__Bacteria.p__Firmicutes.c__Erysipelotrichi.o__Erysipelotrichales	0.64742688	0.21749248	0.85741229	-0.54561643	0.29208975	-0.22043965	-0.86382444	0.10942317	0.10239411
k__Bacteria.p__Firmicutes.c__Erysipelotrichi.o__Erysipelotrichales.f__Erysipelotrichaceae	0.64742688	0.21749248	0.85741229	-0.54561643	0.29208975	-0.22043965	-0.86382444	0.10942317	0.10239411
k__Bacteria.p__Firmicutes.c__Clostridia.o__Clostridiales.f__Ruminococcaceae.g__	-0.80645378	0.0570509	0.46233324	0.51353552	0.54018841	-0.97393275	0.54018841	-0.28465686	0.83184468
k__Bacteria.p__Firmicutes.c__Clostridia.o__Clostridiales.f__Lachnospiraceae.g__Clostridium	0.83796682	-0.60061007	-0.88940551	-0.79982499	-0.64689282	0.98041692	0.43228642	0.4274922	-0.66463045
k__Bacteria.p__Firmicutes.c__Bacilli.o__Lactobacillales.f__Streptococcaceae	-0.25021179	-0.71167948	0.80537784	0.73013156	-0.37949342	0.21783453	-0.85016539	-0.8181062	0.57650892
k__Bacteria.p__Firmicutes.c__Bacilli.o__Lactobacillales.f__Streptococcaceae.g__Streptococcus	-0.25021179	-0.71167948	0.80537784	0.73013156	-0.37949342	0.21783453	-0.85016539	-0.8181062	0.57650892
k__Bacteria.p__Firmicutes.c__Clostridia.o__Clostridiales.f__Ruminococcaceae.g__Faecalibacterium	0.34912168	-0.47487925	0.98689437	-0.80537784	-0.7363069	-0.13062812	-0.799021	-0.92148488	-0.58777364
Dada_3.k__Bacteria.p__Firmicutes.c__Clostridia.o__Clostridiales.f__Lachnospiraceae.g__Ruminococcus.s__lactaris	0.44250747	0.71634351	0.23116388	-0.82356833	0.94732717	0.8235208	0.35502278	-0.62609234	-0.58008146
k__Bacteria.p__Bacteroidetes.c__Bacteroidia.o__Bacteroidales.Other	-0.14384744	-0.12054381	-0.01554163	-0.68575158	0.9210625	-0.05855354	-0.05230453	-0.74115191	0.75995887
k__Bacteria.p__Bacteroidetes.c__Bacteroidia.o__Bacteroidales.Other.Other	-0.14384744	-0.12054381	-0.01554163	-0.68575158	0.9210625	-0.05855354	-0.05230453	-0.74115191	0.75995887
k__Bacteria.p__Actinobacteria.c__Coriobacteriia.o__Coriobacteriales.f__Coriobacteriaceae.Other	-0.02812863	-0.90765921	-0.6726398	0.0247555	0.96035295	-0.79725547	-0.06230058	0.06346464	0.18774056
Dada_6.k__Bacteria.p__Firmicutes.c__Clostridia.o__Clostridiales.f__Lachnospiraceae.g__Ruminococcus.s__lactaris	0.72689638	0.45923038	0.43440828	0.9734652	-0.22789272	0.44412468	0.61196563	-0.59456426	-0.72689638
k__Bacteria.p__Proteobacteria.c__Deltaproteobacteria	-0.45421418	0.95358034	0.14791881	0.42469542	-0.1916901	0.80938534	0.79015552	-0.44422051	0.3024753
k__Bacteria.p__Proteobacteria.c__Deltaproteobacteria.o__Desulfovibrionales	-0.45421418	0.95358034	0.14791881	0.42469542	-0.1916901	0.80938534	0.79015552	-0.44422051	0.3024753
k__Bacteria.p__Proteobacteria.c__Deltaproteobacteria.o__Desulfovibrionales.f__Desulfovibrionaceae	-0.45421418	0.95358034	0.14791881	0.42469542	-0.1916901	0.80938534	0.79015552	-0.44422051	0.3024753
k__Bacteria.p__Firmicutes.c__Clostridia.o__Clostridiales.f__Ruminococcaceae.g__Gemmiger	-0.21842942	0.06857932	0.5717584	0.6533994	0.26158789	-0.86787525	0.71444013	0.58881801	0.71444844
k__Bacteria.p__Proteobacteria.c__Deltaproteobacteria.o__Desulfovibrionales.f__Desulfovibrionaceae.g__Desulfovibrio	-0.45421418	0.95358034	0.14791881	0.42469542	-0.1916901	0.80938534	0.79015552	-0.44422051	0.3024753
Dada_17.k__Bacteria.p__Actinobacteria.c__Actinobacteria.o__Bifidobacteriales.f__Bifidobacteriaceae.g__Bifidobacterium.s__brev	-0.50411517	-0.88711127	-0.52567145	-0.55886698	0.18426156	0.6045545	0.92681186	-0.62813738	-0.96004098
k__Bacteria.p__Proteobacteria.c__Gammaproteobacteria	-0.23572056	-0.86079452	0.66412959	-0.62815813	-0.14855453	-0.82157431	0.78287818	-0.13715909	0.25610347
k__Bacteria.p__Actinobacteria.c__Coriobacteriia.o__Coriobacteriales.f__Coriobacteriaceae.g__Collinsella	0.61635589	0.828118	0.85424258	0.20435019	-0.83463145	0.97334727	-0.95338677	0.00365963	-0.50411517
Dada_1.k__Bacteria.p__Actinobacteria.c__Coriobacteriia.o__Coriobacteriales.f__Coriobacteriaceae.g__Collinsella.s__	0.66875526	-0.76903597	0.95317078	0.10907464	-0.87996557	-0.87332184	-0.84042453	0.00274285	-0.44520636
Dada_2.k__Bacteria.p__Actinobacteria.c__Coriobacteriia.o__Coriobacteriales.f__Coriobacteriaceae.g__Collinsella.s__	0.74352816	0.47577379	0.63251279	0.46546742	-0.78833963	0.49668178	0.69955461	0.01718532	-0.70578657
k__Bacteria.p__Proteobacteria.c__Gammaproteobacteria.o__Enterobacteriales	-0.25041529	0.97322928	0.64450134	-0.66875526	-0.21436155	-0.80123574	0.67486524	-0.12063276	0.24356594
k__Bacteria.p__Proteobacteria.c__Gammaproteobacteria.o__Enterobacteriales.f__Enterobacteriaceae	-0.25041529	0.97322928	0.64450134	-0.66875526	-0.21436155	-0.80123574	0.67486524	-0.12063276	0.24356594
k__Bacteria.p__Firmicutes.c__Clostridia.o__Clostridiales.f__Lachnospiraceae.g__Roseburia	0.23026466	-0.29819551	0.95317078	-0.06617032	-0.83387054	0.82075553	-0.35608205	0.54023385	-0.27915192
k__Bacteria.p__Proteobacteria.c__Gammaproteobacteria.o__Enterobacteriales.f__Enterobacteriaceae.Other	-0.25041529	0.97322928	0.64450134	-0.66875526	-0.21436155	-0.80123574	0.67486524	-0.12063276	0.24356594
Dada_4.k__Bacteria.p__Actinobacteria.c__Coriobacteriia.o__Coriobacteriales.f__Coriobacteriaceae.g__Collinsella.s__	0.58359278	-0.75501779	0.92609193	0.15903948	-0.64882509	-0.95291491	-0.80027036	0.00196752	-0.27665879
Dada_5.k__Bacteria.p__Actinobacteria.c__Coriobacteriia.o__Coriobacteriales.f__Coriobacteriaceae.g__Collinsella.s__	0.34924131	0.89933725	0.60696369	0.26928019	0.89266576	0.91268049	0.94621079	0.00180611	-0.23441935
Dada_8.k__Bacteria.p__Firmicutes.c__Clostridia.o__Clostridiales.f__Lachnospiraceae.g__Ruminococcus.s__lactaris	0.02656326	0.31967673	-0.9663672	-0.09331488	-0.55497154	-0.26191038	0.28796868	0.61878293	0.72952865
k__Bacteria.Other	-0.74224139	0.74861726	0.54934474	0.06477253	0.51067129	0.39893531	-0.43286915	-0.80678843	0.18550986
k__Bacteria.Other.Other	-0.74224139	0.74861726	0.54934474	0.06477253	0.51067129	0.39893531	-0.43286915	-0.80678843	0.18550986
k__Bacteria.Other.Other.Other	-0.74224139	0.74861726	0.54934474	0.06477253	0.51067129	0.39893531	-0.43286915	-0.80678843	0.18550986
k__Bacteria.Other.Other.Other.Other	-0.74224139	0.74861726	0.54934474	0.06477253	0.51067129	0.39893531	-0.43286915	-0.80678843	0.18550986
k__Bacteria.p__Firmicutes.c__Clostridia.o__Clostridiales.f__Veillonellaceae	-0.0751806	-0.2182282	-0.92609193	0.32380941	-0.68562779	0.8660288	-0.92608974	-0.83301425	0.97981649
k__Bacteria.Other.Other.Other.Other.Other	-0.74224139	0.74861726	0.54934474	0.06477253	0.51067129	0.39893531	-0.43286915	-0.80678843	0.18550986
k__Bacteria.p__Firmicutes.c__Clostridia.o__Clostridiales.f__Ruminococcaceae.g__Butyricicoccus	0.01113829	0.13332534	0.41335715	0.78079077	0.28415973	-0.10532542	0.45291845	0.00014266	-0.51611847
Dada_10.k__Bacteria.p__Firmicutes.c__Clostridia.o__Clostridiales.f__Lachnospiraceae.g__Ruminococcus.s__lactaris	0.42528932	-0.49205954	-0.72166772	0.51918117	-0.89875912	0.54130312	-0.31280664	0.07768239	-0.65917849
Dada_15.k__Bacteria.p__Firmicutes.c__Clostridia.o__Clostridiales.f__Lachnospiraceae.g__Ruminococcus.s__lactaris	0.05970928	0.21874179	0.06204544	-0.74722235	-0.12894723	0.98646224	0.07350401	0.6108005	0.72166772
Dada_33.k__Bacteria.p__Firmicutes.c__Clostridia.o__Clostridiales.f__Lachnospiraceae.g__Ruminococcus.s__lactaris	0.10854919	0.15400226	0.49468795	-0.21900846	-0.67574892	-0.50542659	0.48929678	0.2821126	0.63264298
Dada_39.k__Bacteria.p__Actinobacteria.c__Coriobacteriia.o__Coriobacteriales.f__Coriobacteriaceae.g__Collinsella.s__	0.42233654	0.85103721	0.85104151	0.25270243	-0.87115879	-0.90485222	-0.8577343	0.00065917	-0.39333244
Dada_41.k__Bacteria.p__Actinobacteria.c__Actinobacteria.o__Bifidobacteriales.f__Bifidobacteriaceae.g__Bifidobacterium.s__brev	-0.85104151	0.67574892	-0.69457712	-0.42233654	0.75212596	0.06896849	0.97956966	-0.30566182	-0.29769956
Dada_61.k__Bacteria.p__Actinobacteria.c__Actinobacteria.o__Bifidobacteriales.f__Bifidobacteriaceae.g__Bifidobacterium.s__brev	-0.67360148	0.89060235	-0.43406896	-0.43406896	0.77007494	0.1059869	-0.64250128	-0.20633621	-0.21897938
k__Bacteria.p__Bacteroidetes.c__Bacteroidia.o__Bacteroidales.f__Porphyromonadaceae	-0.29827872	0.74392325	0.71165553	-0.89740359	-0.50252794	-0.63015426	0.37130939	-0.64249165	0.19428198
k__Bacteria.p__Firmicutes.c__Clostridia.o__Clostridiales.f__Clostridiaceae	-0.55309218	0.27118555	0.49707815	0.03992974	0.20945289	-0.19416481	-0.58811349	0.47552399	-0.75044185
k__Bacteria.p__Firmicutes.c__Erysipelotrichi.o__Erysipelotrichales.f__Erysipelotrichaceae.g__Clostridium	0.78986954	0.73095523	0.72450732	-0.39476	-0.25290644	0.71158316	0.66732736	-0.40921478	0.12387579
Dada_30.k__Bacteria.p__Firmicutes.c__Clostridia.o__Clostridiales.f__Lachnospiraceae.g__Ruminococcus.s__lactaris	0.16838524	0.86244376	0.38159615	0.5852264	0.04631202	-0.49362617	0.47746651	0.04004933	0.9792662
k__Bacteria.p__Firmicutes.c__Bacilli.o__Bacillales	0.27886604	-0.59118539	-0.69665984	-0.63374464	-0.29853264	-0.17369062	-0.43049076	0.53277928	-0.06500688
k__Bacteria.p__Firmicutes.c__Clostridia.o__Clostridiales.f__Clostridiaceae.g__Clostridium	-0.39109504	0.34061382	0.62145533	0.06252747	0.22518739	-0.23517404	-0.43049076	0.48284122	-0.67121205
Dada_40.k__Bacteria.p__Firmicutes.c__Clostridia.o__Clostridiales.f__Ruminococcaceae.g__Gemmiger.s__formicilis	-0.49556202	0.01341144	0.33651473	0.20834336	0.19900851	-0.89570438	0.64958483	0.39669356	-0.82029695
Dada_53.k__Bacteria.p__Firmicutes.c__Clostridia.o__Clostridiales.f__Ruminococcaceae	0.51792905	-0.69418801	0.68775478	0.46300273	0.26715604	-0.38693356	-0.5820113	0.30252702	0.25603388
Dada_66.k__Bacteria.p__Actinobacteria.c__Actinobacteria.o__Bifidobacteriales.f__Bifidobacteriaceae.g__Bifidobacterium.s__brev	-0.6123314	0.75975303	-0.51792905	-0.38227944	0.91649684	0.15941879	-0.70713739	-0.21470539	-0.2634213
k__Bacteria.p__Verrucomicrobia	-0.32780309	-0.6813273	-0.20209415	-0.15189366	-0.60011347	0.15178728	-0.46833242	-0.021071	-0.88882382
k__Bacteria.p__Verrucomicrobia.c__Verrucomicrobiae	-0.32780309	-0.6813273	-0.20209415	-0.15189366	-0.60011347	0.15178728	-0.46833242	-0.021071	-0.88882382
k__Bacteria.p__Verrucomicrobia.c__Verrucomicrobiae.o__Verrucomicrobiales	-0.32780309	-0.6813273	-0.20209415	-0.15189366	-0.60011347	0.15178728	-0.46833242	-0.021071	-0.88882382
k__Bacteria.p__Verrucomicrobia.c__Verrucomicrobiae.o__Verrucomicrobiales.f__Verrucomicrobiaceae	-0.32780309	-0.6813273	-0.20209415	-0.15189366	-0.60011347	0.15178728	-0.46833242	-0.021071	-0.88882382
k__Bacteria.p__Bacteroidetes.c__Bacteroidia.o__Bacteroidales.f__Porphyromonadaceae.g__Macellibacteroides	-0.54080718	0.72017206	0.87503464	0.62464753	-0.86815027	-0.58192398	0.41646585	-0.72671284	0.36353373
Dada_12.k__Bacteria.p__Firmicutes.c__Clostridia.o__Clostridiales.f__Ruminococcaceae.g__Gemmiger.s__formicilis	-0.6089953	0.20096525	0.94375364	-0.69798595	0.18881801	-0.55449849	0.59669359	0.49706853	0.94375364
Dada_31.k__Bacteria.p__Firmicutes.c__Clostridia.o__Clostridiales.f__Ruminococcaceae.g__Gemmiger.s__formicilis	-0.42735874	0.03210482	0.42735874	-0.84615997	0.16083245	0.96481918	0.23035994	0.50831081	0.73753003
k__Bacteria.p__Firmicutes.c__Bacilli.o__Lactobacillales.Other	-0.44818577	0.21044297	-0.79133829	-0.4915219	-0.6028253	0.39707714	-0.97889198	-0.00814785	0.13844416
k__Bacteria.p__Firmicutes.c__Bacilli.o__Lactobacillales.Other.Other	-0.44818577	0.21044297	-0.79133829	-0.4915219	-0.6028253	0.39707714	-0.97889198	-0.00814785	0.13844416

Supplementary Figure 4A: Time-point: T1_*Heatmap*

	Spearman's rho								
	tryptophan	n_acetyltrypto	indolelactic	indoleacetic	indolepropion	indoleacetami	indolealdehyd	x3_methylindo	indoxyl_s
k__Bacteria.p__Actinobacteria	-0.118431373	-0.112985493	-0.132653102	-0.2118478	-0.236078431	-0.05886972	-0.124067616	-0.274833328	-0.071372549
k__Bacteria.p__Firmicutes	-0.094901961	-0.037661831	0.096546341	0.048646532	-0.181176471	0.04003141	-0.076953332	-0.062819046	0.168627451
k__Bacteria.p__Actinobacteria.c__Coriobacteriia	-0.160784314	-0.056492747	-0.102825777	-0.232247958	-0.316078431	-0.052590283	-0.182175234	-0.268551423	0.099607843
k__Bacteria.p__Firmicutes.c__Clostridia	0.117839245	0.10881737	0.015669257	0.011541236	0.220021528	-0.011545768	0.082502185	0.127878387	-0.11124684
k__Bacteria.p__Actinobacteria.c__Coriobacteriia.o__Coriobacteriales	-0.160784314	-0.056492747	-0.102825777	-0.232247958	-0.316078431	-0.052590283	-0.182175234	-0.268551423	0.099607843
k__Bacteria.p__Firmicutes.c__Clostridia.o__Clostridiales	0.155745576	0.103871126	0.07669689	0.095627386	0.22166963	0.003298791	0.120453191	0.192230092	-0.096413928
k__Bacteria.p__Actinobacteria.c__Coriobacteriia.o__Coriobacteriales.f__Coriobacteriaceae	-0.160784314	-0.056492747	-0.102825777	-0.232247958	-0.316078431	-0.052590283	-0.182175234	-0.268551423	0.099607843
k__Bacteria.p__Firmicutes.c__Clostridia.o__Clostridiales.f__Lachnospiraceae	0.079215686	-0.007846215	0.085557326	-0.08473912	-0.082352941	-0.025902677	0	0.015704762	-0.021176471
k__Bacteria.p__Firmicutes.c__Clostridia.o__Clostridiales.f__Ruminococcaceae	0.008627451	0.160062782	-0.005494507	0.047077289	0.022745098	0.049450565	0.089517141	0.048684761	0.04
k__Bacteria.p__Firmicutes.c__Clostridia.o__Clostridiales.f__Lachnospiraceae.Other	0.007058824	0.029815616	0.057299861	0.031384859	-0.054117647	-0.153061272	-0.103651426	0.075382856	0.082352941
k__Bacteria.p__Firmicutes.c__Clostridia.o__Clostridiales.f__Ruminococcaceae.Other	0.024313725	-0.004707729	-0.05886972	-0.091016092	0.088627451	0.146781835	0.069100951	-0.168040949	0.146666667
k__Bacteria.p__Bacteroidetes	-0.262745098	-0.086308363	-0.105965496	-0.194586127	-0.091764706	0.096546341	-0.158618092	-0.207302853	-0.005490196
k__Bacteria.p__Bacteroidetes.c__Bacteroidia	-0.242352941	-0.078462148	-0.113814792	-0.202432342	-0.096470588	0.104395637	-0.157047616	-0.193168567	-0.003921569
k__Bacteria.p__Bacteroidetes.c__Bacteroidia.o__Bacteroidales	-0.242352941	-0.078462148	-0.113814792	-0.202432342	-0.096470588	0.104395637	-0.157047616	-0.193168567	-0.003921569
k__Bacteria.p__Firmicutes.c__Clostridia.o__Clostridiales.Other	-0.029019608	-0.04236956	-0.055730001	-0.043938803	0.110588235	0.021193099	-0.04083238	0.017275238	0.105882353
k__Bacteria.p__Firmicutes.c__Clostridia.o__Clostridiales.Other.Other	-0.029019608	-0.04236956	-0.055730001	-0.043938803	0.110588235	0.021193099	-0.04083238	0.017275238	0.105882353
k__Bacteria.p__Firmicutes.c__Clostridia.o__Clostridiales.f__Lachnospiraceae.g__Ruminococcus	0.083921569	0.098862307	0.11695451	0	-0.061960784	0.159340708	0.098939998	0.009422857	0.071372549
k__Bacteria.p__Proteobacteria	-0.329799993	0.041633948	-0.114734783	-0.007069916	-0.249705709	0.02357564	-0.16745283	-0.145440252	0.149195235
k__Bacteria.p__Bacteroidetes.c__Bacteroidia.o__Bacteroidales.f__Bacteroidaceae	-0.095799046	-0.000785546	0.07229863	-0.120188568	-0.116215236	0.011001965	0.030660377	-0.14072327	-0.007852381
k__Bacteria.p__Bacteroidetes.c__Bacteroidia.o__Bacteroidales.f__Bacteroidaceae.g__Bacteroides	-0.095799046	-0.000785546	0.07229863	-0.120188568	-0.116215236	0.011001965	0.030660377	-0.14072327	-0.007852381
k__Bacteria.p__Actinobacteria.c__Actinobacteria	-0.074685742	-0.198191496	-0.104642144	-0.06763678	-0.03852212	-0.041699501	-0.004722553	-0.146399158	-0.239780541
k__Bacteria.p__Firmicutes.c__Bacilli	-0.049606684	-0.026782366	0.111111663	0.105554031	-0.185040804	0.060678	-0.014190042	-0.05833684	0.092126698
k__Bacteria.p__Firmicutes.c__Erysipelotrichi	0.070079283	0.015754333	0.00394013	-0.100827731	0.063780022	0.008668286	-0.074103554	0.107213652	-0.038582976
k__Bacteria.p__Firmicutes.c__Erysipelotrichi.o__Erysipelotrichales	0.070079283	0.015754333	0.00394013	-0.100827731	0.063780022	0.008668286	-0.074103554	0.107213652	-0.038582976
k__Bacteria.p__Firmicutes.c__Erysipelotrichi.o__Erysipelotrichales.f__Erysipelotrichaceae	0.070079283	0.015754333	0.00394013	-0.100827731	0.063780022	0.008668286	-0.074103554	0.107213652	-0.038582976
k__Bacteria.p__Bacteroidetes.c__Bacteroidia.o__Bacteroidales.f__Rikenellaceae	-0.209865607	-0.01183917	-0.105804789	-0.037096067	0.04576017	0.172130179	-0.063981761	-0.097157489	0.088364466
k__Bacteria.p__Firmicutes.c__Clostridia.o__Clostridiales.f__	0.157793689	-0.05603874	0.022108463	0.068667188	0.201975922	0.056850334	0.090838303	0.176147318	-0.018935243
k__Bacteria.p__Firmicutes.c__Clostridia.o__Clostridiales.f__.g__	0.157793689	-0.05603874	0.022108463	0.068667188	0.201975922	0.056850334	0.090838303	0.176147318	-0.018935243
k__Bacteria.p__Bacteroidetes.c__Bacteroidia.o__Bacteroidales.f__Rikenellaceae.g__Alistipes	-0.139190248	-0.00395582	-0.069649772	0.008702804	-0.003163415	0.212115216	-0.029296072	-0.083137503	0.061686587
k__Bacteria.p__Firmicutes.c__Clostridia.o__Clostridiales.f__Ruminococcaceae.g__Clostridium	0.064850002	0.053007988	-0.058569127	-0.007120476	-0.039542684	0.011080646	0.008709643	0.064134645	-0.012653659
k__Bacteria.p__Proteobacteria.c__Deltaproteobacteria	-0.371949966	-0.068230807	-0.064289136	-0.130114563	-0.19112994	0.081750383	-0.195325221	-0.193737211	0.046791147
k__Bacteria.p__Proteobacteria.c__Deltaproteobacteria.o__Desulfovibrionales	-0.371949966	-0.068230807	-0.064289136	-0.130114563	-0.19112994	0.081750383	-0.195325221	-0.193737211	0.046791147
k__Bacteria.p__Proteobacteria.c__Deltaproteobacteria.o__Desulfovibrionales.f__Desulfovibrionaceae	-0.373536107	-0.066644044	-0.065876522	-0.1285278	-0.189543799	0.083337769	-0.193737211	-0.192149201	0.045205007
k__Bacteria.p__Proteobacteria.c__Deltaproteobacteria.o__Desulfovibrionales.f__Desulfovibrionaceae.g__Desulfovibrio	-0.370363826	-0.072991096	-0.072226067	-0.134874852	-0.189543799	0.076988225	-0.196913231	-0.188973181	0.045205007
k__Bacteria.p__Firmicutes.c__Bacilli.o__Lactobacillales	-0.166094666	-0.024764207	0.051146182	0.091867219	-0.161303474	-0.007991591	-0.074351004	-0.077548897	0.178871179
k__Bacteria.p__Firmicutes.c__Clostridia.o__Clostridiales.f__Ruminococcaceae.g__	-0.073464949	0.019971135	-0.043154591	0.162165613	-0.014373577	-0.019179818	0.023184722	0.09353836	0.054300179
k__Bacteria.p__Actinobacteria.c__Actinobacteria.o__Bifidobacteriales	-0.068954632	-0.1901007	-0.078637909	-0.085826055	-0.049711479	-0.046540803	-0.004013715	-0.142085512	-0.226107048
k__Bacteria.p__Actinobacteria.c__Actinobacteria.o__Bifidobacteriales.f__Bifidobacteriaceae	-0.068954632	-0.1901007	-0.078637909	-0.085826055	-0.049711479	-0.046540803	-0.004013715	-0.142085512	-0.226107048
k__Bacteria.p__Bacteroidetes.c__Bacteroidia.o__Bacteroidales.Other	-0.254971778	-0.169245771	-0.239123438	-0.308813374	-0.113855322	-0.120364147	-0.313872514	-0.249653074	-0.109044534
k__Bacteria.p__Actinobacteria.c__Actinobacteria.o__Bifidobacteriales.f__Bifidobacteriaceae.g__Bifidobacterium	-0.068954632	-0.1901007	-0.078637909	-0.085826055	-0.049711479	-0.046540803	-0.004013715	-0.142085512	-0.226107048
k__Bacteria.p__Actinobacteria.c__Coriobacteriia.o__Coriobacteriales.f__Coriobacteriaceae.g__Eggerthella	-0.25817897	-0.028073943	-0.107525305	-0.317636613	-0.078576208	-0.001604855	-0.191855578	-0.167773288	0.123476899
k__Bacteria.p__Bacteroidetes.c__Bacteroidia.o__Bacteroidales.Other.Other	-0.254971778	-0.169245771	-0.239123438	-0.308813374	-0.113855322	-0.120364147	-0.313872514	-0.249653074	-0.109044534
k__Bacteria.p__Firmicutes.c__Clostridia.o__Clostridiales.f__Lachnospiraceae.g__Clostridium	-0.041693498	-0.020854929	0.073823343	-0.109889434	0	0.022467974	0.028096005	-0.042545379	0.059333055
k__Bacteria.p__Firmicutes.c__Clostridia.o__Clostridiales.f__Ruminococcaceae.g__Faecalibacterium	0.214881876	0.061762675	0.11233987	0.089034505	0.088197785	-0.006419421	0.118003222	0.161351344	-0.149134436
k__Bacteria.p__Firmicutes.c__Clostridia.o__Clostridiales.f__Veillonellaceae	-0.326202121	-0.024172601	-0.010478907	-0.001611507	-0.023357683	-0.081413049	-0.014514959	-0.119345214	0.024968557
k__Bacteria.p__Firmicutes.c__Clostridia.o__Clostridiales.f__Lachnospiraceae.g__Dorea	0.086612707	-0.07449996	-0.09640192	0.019434772	-0.028331259	-0.090731219	-0.061591809	-0.035658416	-0.081755919
k__Bacteria.p__Firmicutes.Other	-0.190451726	0.111547541	0.073308183	-0.160400479	-0.133478988	0.070050042	0.000814855	-0.036668497	0.198590689
k__Bacteria.p__Firmicutes.Other.Other	-0.190451726	0.111547541	0.073308183	-0.160400479	-0.133478988	0.070050042	0.000814855	-0.036668497	0.198590689
k__Bacteria.p__Firmicutes.Other.Other.Other	-0.190451726	0.111547541	0.073308183	-0.160400479	-0.133478988	0.070050042	0.000814855	-0.036668497	0.198590689
k__Bacteria.p__Firmicutes.Other.Other.Other.Other	-0.190451726	0.111547541	0.073308183	-0.160400479	-0.133478988	0.070050042	0.000814855	-0.036668497	0.198590689
k__Bacteria.p__Firmicutes.c__Bacilli.o__Lactobacillales.f__Streptococcaceae	-0.207962818	-0.056516004	-0.00983273	0.072897454	-0.119537683	-0.065551533	-0.069675876	-0.0860702	0.109712668
k__Bacteria.p__Actinobacteria.c__Coriobacteriia.o__Coriobacteriales.f__Coriobacteriaceae.Other	-0.037662558	0.146613981	-0.090133358	0.11385108	-0.022925035	-0.060635168	0.025411202	0.159844657	0.15720024
k__Bacteria.p__Firmicutes.c__Bacilli.o__Lactobacillales.f__Streptococcaceae.g__Streptococcus	-0.207962818	-0.056516004	-0.00983273	0.072897454	-0.119537683	-0.065551533	-0.069675876	-0.0860702	0.109712668
k__Bacteria.p__Firmicutes.c__Clostridia.o__Clostridiales.f__Ruminococcaceae.g__Gemmiger	-0.099710131	0.056057433	0.015669257	0.112114866	-0.022249368	-0.02556563	-0.04455118	0.110552928	-0.040378483
Dada_1.k__Bacteria.p__Actinobacteria.c__Coriobacteriia.o__Coriobacteriales.f__Coriobacteriaceae.g__Collinsella.s__	0.0267546	-0.034292782	-0.092041154	0.092841433	-0.145478136	-0.006693902	-0.081195461	-0.109655725	0.031771087
Dada_4.k__Bacteria.p__Actinobacteria.c__Coriobacteriia.o__Coriobacteriales.f__Coriobacteriaceae.g__Collinsella.s__	0.023410275	-0.020910233	-0.102082007	0.09618707	-0.162199761	0.006693902	-0.081195461	-0.119700524	0.021738112
k__Bacteria.Other	-0.24246356	0.066076335	-0.07865335	0.06105788	0.043476225	0.195796637	-0.09124026	0	0.172232736
k__Bacteria.Other.Other	-0.24246356	0.066076335	-0.07865335	0.06105788	0.043476225	0.195796637	-0.09124026	0	0.172232736
k__Bacteria.p__Proteobacteria.c__Gammaproteobacteria	-0.259185185	0.026765098	-0.107102434	0.025928689	-0.280923297	-0.008367378	-0.136441857	-0.149834922	0.096985424
k__Bacteria.Other.Other.Other	-0.24246356	0.066076335	-0.07865335	0.06105788	0.043476225	0.195796637	-0.09124026	0	0.172232736
k__Bacteria.Other.Other.Other.Other	-0.24246356	0.066076335	-0.07865335	0.06105788	0.043476225	0.195796637	-0.09124026	0	0.172232736
k__Bacteria.Other.Other.Other.Other.Other	-0.24246356	0.066076335	-0.07865335	0.06105788	0.043476225	0.195796637	-0.09124026	0	0.172232736
k__Bacteria.p__Actinobacteria.c__Coriobacteriia.o__Coriobacteriales.f__Coriobacteriaceae.g__Collinsella	0.035115412	-0.042656875	-0.093714629	0.074440428	-0.157183273	0.018408231	-0.079521327	-0.144812523	0.03344325
Dada_6.k__Bacteria.p__Firmicutes.c__Clostridia.o__Clostridiales.f__Lachnospiraceae.g__Ruminococcus.s__lactaris	0.118001739	-0.105400037	-0.08941433	-0.191406467	-0.082601217	0.096162581	-0.130798754	-0.08354243	-0.065743826
k__Bacteria.p__Proteobacteria.c__Gammaproteobacteria.o__Enterobacteriales	-0.259603825	0.050592018	-0.082666079	0.02782561	-0.257918086	0.018557691	-0.112233769	-0.122360124	0.111258782
k__Bacteria.p__Firmicutes.c__Clostridia.o__Clostridiales.f__Peptostreptococcaceae	0.079229739	-0.08684963	-0.074230765	0.02782561	0.003371478	-0.056516605	0.029535202	-0.027847477	-0.318604695
k__Bacteria.p__Proteobacteria.c__Gammaproteobacteria.o__Enterobacteriales.f__Enterobacteriaceae	-0.259603825	0.050592018	-0.082666079	0.02782561	-0.257918086	0.018557691	-0.112233769	-0.122360124	0.111258782
k__Bacteria.p__Firmicutes.c__Clostridia.o__Clostridiales.f__Peptostreptococcaceae.g__Clostridium	0.077544	-0.098654434	-0.082666079	0.022766408	0.001685739	-0.051455416	0.027847477	-0.036286106	-0.323661912
k__Bacteria.p__Proteobacteria.c__Gammaproteobacteria.o__Enterobacteriales.f__Enterobacteriaceae.Other	-0.259603825	0.050592018	-0.082666079	0.02782561	-0.257918086	0.018557691	-0.112233769	-0.122360124	0.111258782
Dada_2.k__Bacteria.p__Actinobacteria.c__Coriobacteriia.o__Coriobacteriales.f__Coriobacteriaceae.g__Collinsella.s__	0.046761962	-0.064641885	-0.079132316	0.003402204	-0.172594152	0.03828983	-0.098741465	-0.180458539	0.034858917
Dada_3.k__Bacteria.p__Firmicutes.c__Clostridia.o__Clostridiales.f__Lachnospiraceae.g__Ruminococcus.s__lactaris	0.107977622	-0.073147396	-0.110615066	-0.095261725	0.080770662	0.011912392	-0.066395123	0	-0.182796762
k__Bacteria.p__Firmicutes.c__Bacilli.o__Bacillales	0.323932867	0.060389129	0.028079209	-0.063791333	-0.046761962	0.085939397	0.051073171	0.10725366	-0.162391542
k__Bacteria.p__Bacteroidetes.c__Bacteroidia.o__Bacteroidales.f__Porphyromonadaceae	-0.164091977	-0.132685974	-0.092746478	-0.023815431	0.014453697	0.017868588	-0.013619512	-0.023834147	-0.012753262
k__Bacteria.p__Firmicutes.c__Clostridia.o__Clostridiales.f__Lachnospiraceae.g__Roseburia	0.119880667	-0.06123968	-0.029780979	-0.066342987	0.130083277	-0.079983201	0.040007318	0.090229269	-0.211704157
k__Bacteria.p__Firmicutes.c__Clostridia.o__Clostridiales.f__Ruminococcaceae.g__Butyricicoccus	0.131783712	0.110571645	0.091044708	0.307899503	0.101175882	-0.210168625	0.168541465	0.144707319	-0.068867617
k__Bacteria.p__Firmicutes.c__Clostridia.o__Clostridiales.f__Veillonellaceae.g__Succinispira	-0.278021122	-0.049331965	-0.091044708	-0.035723147	0.153889367	-0.068921695	-0.057882927	-0.114914635	0.118180232
Dada_5.k__Bacteria.p__Actinobacteria.c__Coriobacteriia.o__Coriobacteriales.f__Coriobacteriaceae.g__Collinsella.s__	0.069511254	-0.006868003	-0.038647687	0.056661024	-0.121001072	0.024906287	-0.039522051	-0.162384078	0.060929618
k__Bacteria.p__Verrucomicrobia	-0.193086816	-0.078982033	-0.181214711	-0.179426575	0.018021436	0.181214711	-0.138327178	-0.036085351	0.012872454
k__Bacteria.p__Verrucomicrobia.c__Verrucomicrobiae	-0.193086816	-0.078982033	-0.181214711	-0.179426575	0.018021436	0.181214711	-0.138327178	-0.036085351	0.012872454
k__Bacteria.p__Verrucomicrobia.c__Verrucomicrobiae.o__Verrucomicrobiales	-0.193086816	-0.078982033	-0.181214711	-0.179426575	0.018021436	0.181214711	-0.138327178	-0.036085351	0.012872454
k__Bacteria.p__Bacteroidetes.c__Bacteroidia.o__Bacteroidales.f__.Barnesiellaceae.	0.114135763	0.14251106	0.040365362	0.115039048	0.175923544	0.03778885	0.067874826	0.24658323	0.011156127
k__Bacteria.p__Verrucomicrobia.c__Verrucomicrobiae.o__Verrucomicrobiales.f__Verrucomicrobiaceae	-0.193086816	-0.078982033	-0.181214711	-0.179426575	0.018021436	0.181214711	-0.138327178	-0.036085351	0.012872454
k__Bacteria.p__Bacteroidetes.c__Bacteroidia.o__Bacteroidales.f__.Barnesiellaceae..g__Barnesiella	0.114135763	0.14251106	0.040365362	0.115039048	0.175923544	0.03778885	0.067874826	0.24658323	0.011156127
Dada_39.k__Bacteria.p__Actinobacteria.c__Coriobacteriia.o__Coriobacteriales.f__Coriobacteriaceae.g__Collinsella.s__	-0.006934013	-0.049424228	-0.147463477	0.054626778	-0.162949303	-0.004337161	-0.084173993	-0.125827103	0.010401019
Dada_44.k__Bacteria.p__Firmicutes.c__Clostridia.o__Clostridiales.f__Ruminococcaceae.g__Faecalibacterium.s__	0.123078729	0.002601275	0.090212951	0.228045121	-0.036403568	0.068527145	0.177025717	0.056405253	-0.169883316
Dada_15.k__Bacteria.p__Firmicutes.c__Clostridia.o__Clostridiales.f__Lachnospiraceae.g__Ruminococcus.s__lactaris	0.06307423	-0.144601828	-0.049972975	-0.079750099	-0.099867531	0.073644384	-0.016664205	0.021049522	-0.036793301
k__Bacteria.p__Firmicutes.c__Erysipelotrichi.o__Erysipelotrichales.f__Erysipelotrichaceae.g__Clostridium	-0.094611345	-0.067480853	-0.033315317	-0.32162952	-0.113884026	0	-0.160502605	-0.13594483	-0.019272681
Dada_12.k__Bacteria.p__Firmicutes.c__Clostridia.o__Clostridiales.f__Ruminococcaceae.g__Gemmiger.s__formicilis	0.004484485	0.139970842	0.017054425	0.183038794	-0.036772779	-0.059241688	0.036816118	0.102366767	-0.069061074
Dada_17.k__Bacteria.p__Actinobacteria.c__Actinobacteria.o__Bifidobacteriales.f__Bifidobacteriaceae.g__Bifidobacterium.s__breve	-0.352480544	-0.253024215	-0.254021178	-0.243154476	-0.083411426	0.140025808	-0.172407187	-0.1643256	-0.144400426
k__Bacteria.p__Proteobacteria.c__Betaproteobacteria	-0.009865868	-0.055629437	-0.108609762	0.061012931	0.149781809	0.14092341	-0.01975499	0.209223305	0.004484485
k__Bacteria.p__Firmicutes.c__Bacilli.o__Bacillales.Other	0.219739779	0.060115682	0.018849628	-0.113950622	-0.139019044	0.131947396	0.043101797	0.061060879	-0.112112132
k__Bacteria.p__Firmicutes.c__Bacilli.o__Bacillales.Other.Other	0.219739779	0.060115682	0.018849628	-0.113950622	-0.139019044	0.131947396	0.043101797	0.061060879	-0.112112132
Dada_7.k__Bacteria.p__Proteobacteria.c__Gammaproteobacteria.o__Enterobacteriales.f__Enterobacteriaceae	-0.314383108	0.103623519	-0.053650776	-0.031814238	-0.243510616	0.015458698	-0.08187233	-0.137363576	0.09086217
k__Bacteria.p__Actinobacteria.c__Actinobacteria.o__Actinomycetales	-0.012720704	0.075445193	-0.144584294	0.014543652	0.009086217	-0.146402965	-0.023652006	-0.019103544	0.058151789
k__Bacteria.p__Firmicutes.c__Clostridia.o__Clostridiales.f__Clostridiaceae	-0.003634487	-0.051811759	-0.027280056	0.078172128	-0.041796598	-0.056378781	-0.071865712	0.080962638	-0.003634487
k__Bacteria.p__Bacteroidetes.c__Bacteroidia.o__Bacteroidales.f__Porphyromonadaceae.g__Macellibacteroides	-0.139927742	-0.091806802	-0.038192078	0.044539933	0.083593196	-0.010912022	0.009096926	0.009096926	0.001817243
k__Bacteria.p__Firmicutes.c__Clostridia.o__Clostridiales.f__Clostridiaceae.g__Clostridium	-0.003634487	-0.051811759	-0.027280056	0.078172128	-0.041796598	-0.056378781	-0.071865712	0.080962638	-0.003634487
k__Bacteria.p__Verrucomicrobia.c__Verrucomicrobiae.o__Verrucomicrobiales.f__Verrucomicrobiaceae.g__Akkermansia	-0.125389795	-0.037268108	-0.112757563	-0.052720738	0.069055249	0.138218948	-0.054581553	0.000909693	0.014537947
Dada_31.k__Bacteria.p__Firmicutes.c__Clostridia.o__Clostridiales.f__Ruminococcaceae.g__Gemmiger.s__formicilis	-0.0110558	0.214751013	0.067308928	0.102306277	0.009213167	-0.153980698	0.036896099	0.035973696	0.0331674

Supplementary Figure 4B: Time-point: T1_*Merged Spearman's rho and p-values matrix*

	Merge rho+P								
	tryptophan	n_acetyltrypto	indolelactic	indoleacetic	indolepropioni	indoleacetamid	indolealdehyd	x3_methylindc	indoxyl_s
k__Bacteria.p__Actinobacteria	-0.220027922	-0.242147088	-0.16983187	-0.028302384	-0.01449383	-0.542387888	-0.19933924	-0.004468333	-0.459833398
k__Bacteria.p__Firmicutes	-0.325712013	-0.696626028	0.317747278	0.61454692	-0.060623599	0.678683634	-0.425999797	-0.515794805	0.08076311
k__Bacteria.p__Actinobacteria.c__Coriobacteriia	-0.095902127	-0.55866977	-0.287292526	-0.016205518	-0.00106318	-0.586287305	-0.059492041	-0.005468265	0.302296654
k__Bacteria.p__Firmicutes.c__Clostridia	0.237662026	0.275691239	0.875318975	0.907960579	0.027467766	-0.907957749	0.408891304	0.200523432	-0.264940785
k__Bacteria.p__Actinobacteria.c__Coriobacteriia.o__Coriobacteriales	-0.095902127	-0.55866977	-0.287292526	-0.016205518	-0.00106318	-0.586287305	-0.059492041	-0.005468265	0.302296654
k__Bacteria.p__Firmicutes.c__Clostridia.o__Clostridiales	0.118593414	0.298106729	0.442479281	0.338101845	0.026329254	0.973648407	0.227919472	0.054326787	-0.333968828
k__Bacteria.p__Actinobacteria.c__Coriobacteriia.o__Coriobacteriales.f__Coriobacteriaceae	-0.095902127	-0.55866977	-0.287292526	-0.016205518	-0.00106318	-0.586287305	-0.059492041	-0.005468265	0.302296654
k__Bacteria.p__Firmicutes.c__Clostridia.o__Clostridiales.f__Lachnospiraceae	0.412020416	-0.935263185	0.375952027	-0.380362954	-0.393752383	-0.788660331	-1	0.870943389	-0.826416596
k__Bacteria.p__Firmicutes.c__Clostridia.o__Clostridiales.f__Ruminococcaceae	0.928808271	0.097522361	-0.954657332	0.626011211	0.813786437	0.608837845	0.35443366	0.614523566	0.678703647
k__Bacteria.p__Firmicutes.c__Clostridia.o__Clostridiales.f__Lachnospiraceae.Other	0.941726621	0.757584584	0.553207567	0.745257391	-0.575184423	-0.113208361	-0.283613157	0.435503051	0.393752383
k__Bacteria.p__Firmicutes.c__Clostridia.o__Clostridiales.f__Ruminococcaceae.Other	0.801204512	-0.961130583	-0.542387888	-0.34608638	0.358718422	0.128774154	0.474716989	-0.082153528	0.128799381
k__Bacteria.p__Bacteroidetes	-0.006509643	-0.371604089	-0.272829039	-0.043970198	-0.341960499	0.317747278	-0.100828759	-0.03199477	-0.95466032
k__Bacteria.p__Bacteroidetes.c__Bacteroidia	-0.012081213	-0.416649397	-0.238877021	-0.036117725	-0.317779206	0.279997325	-0.104247179	-0.045688297	-0.96760597
k__Bacteria.p__Bacteroidetes.c__Bacteroidia.o__Bacteroidales	-0.012081213	-0.416649397	-0.238877021	-0.036117725	-0.317779206	0.279997325	-0.104247179	-0.045688297	-0.96760597
k__Bacteria.p__Firmicutes.c__Clostridia.o__Clostridiales.Other	-0.763779162	-0.660941347	-0.564131973	-0.649210806	0.252113162	0.826405325	-0.672735429	0.858168299	0.272860679
k__Bacteria.p__Firmicutes.c__Clostridia.o__Clostridiales.Other.Other	-0.763779162	-0.660941347	-0.564131973	-0.649210806	0.252113162	0.826405325	-0.672735429	0.858168299	0.272860679
k__Bacteria.p__Firmicutes.c__Clostridia.o__Clostridiales.f__Lachnospiraceae.g__Ruminococcus	0.384805241	0.306103466	0.226167901	-1	-0.521097423	0.099164509	0.306071554	0.92234825	0.459833398
k__Bacteria.p__Proteobacteria	-0.000645493	0.666800378	-0.235596887	-0.94171767	-0.009789549	0.807453992	-0.083556848	-0.132854713	0.122732107
k__Bacteria.p__Bacteroidetes.c__Bacteroidia.o__Bacteroidales.f__Bacteroidaceae	-0.321671115	-0.993518487	0.454832394	-0.213908542	-0.2292734	0.909450088	0.751368168	-0.145892177	-0.935257502
k__Bacteria.p__Bacteroidetes.c__Bacteroidia.o__Bacteroidales.f__Bacteroidaceae.g__Bacteroides	-0.321671115	-0.993518487	0.454832394	-0.213908542	-0.2292734	0.909450088	0.751368168	-0.145892177	-0.935257502
k__Bacteria.p__Actinobacteria.c__Actinobacteria	-0.440214688	-0.040613938	-0.279859943	-0.484718983	-0.690554877	-0.666738446	-0.961116928	-0.13070891	-0.013212749
k__Bacteria.p__Firmicutes.c__Bacilli	-0.608664419	-0.782305235	0.251821233	0.276149123	-0.056162859	0.531449171	-0.883688813	-0.547551334	0.341694966
k__Bacteria.p__Firmicutes.c__Erysipelotrichi	0.469511243	0.870881478	0.967586034	-0.298224175	0.510370382	0.928764549	-0.444877365	0.269013536	-0.69047737
k__Bacteria.p__Firmicutes.c__Erysipelotrichi.o__Erysipelotrichales	0.469511243	0.870881478	0.967586034	-0.298224175	0.510370382	0.928764549	-0.444877365	0.269013536	-0.69047737
k__Bacteria.p__Firmicutes.c__Erysipelotrichi.o__Erysipelotrichales.f__Erysipelotrichaceae	0.469511243	0.870881478	0.967586034	-0.298224175	0.510370382	0.928764549	-0.444877365	0.269013536	-0.69047737
k__Bacteria.p__Bacteroidetes.c__Bacteroidia.o__Bacteroidales.f__Rikenellaceae	-0.030577335	-0.902937689	-0.275948417	-0.702377051	0.637262921	0.076325706	-0.510165927	-0.317279083	0.362538317
k__Bacteria.p__Firmicutes.c__Clostridia.o__Clostridiales.f__	0.103957628	-0.563780346	0.819918639	0.479369629	0.037412673	0.558289044	0.349777959	0.069812411	-0.845302526
k__Bacteria.p__Firmicutes.c__Clostridia.o__Clostridiales.f__.g__	0.103957628	-0.563780346	0.819918639	0.479369629	0.037412673	0.558289044	0.349777959	0.069812411	-0.845302526
k__Bacteria.p__Bacteroidetes.c__Bacteroidia.o__Bacteroidales.f__Rikenellaceae.g__Alistipes	-0.152257132	-0.967557466	-0.474084792	0.9287019	-0.974044242	0.029251191	-0.763420881	-0.393013339	0.525780047
k__Bacteria.p__Firmicutes.c__Clostridia.o__Clostridiales.f__Ruminococcaceae.g__Clostridium	0.504773792	0.585751168	-0.547198327	-0.941639475	-0.684224129	0.909328943	0.928697271	0.509942255	-0.896451047
k__Bacteria.p__Proteobacteria.c__Deltaproteobacteria	-0.000134782	-0.483900431	-0.509661912	-0.181887546	-0.049799177	0.401777569	-0.045222554	-0.047000319	0.631052112
k__Bacteria.p__Proteobacteria.c__Deltaproteobacteria.o__Desulfovibrionales	-0.000134782	-0.483900431	-0.509661912	-0.181887546	-0.049799177	0.401777569	-0.045222554	-0.047000319	0.631052112
k__Bacteria.p__Proteobacteria.c__Deltaproteobacteria.o__Desulfovibrionales.f__Desulfovibrionaceae	-0.000126162	-0.494124859	-0.499266178	-0.187274206	-0.051726206	0.392699865	-0.047000319	-0.048836514	0.642671406
k__Bacteria.p__Proteobacteria.c__Deltaproteobacteria.o__Desulfovibrionales.f__Desulfovibrionaceae.g__Desulfovibrio	-0.000143954	-0.453927581	-0.458834599	-0.166417867	-0.051726206	0.429756147	-0.043501816	-0.052689877	0.642671406
k__Bacteria.p__Firmicutes.c__Bacilli.o__Lactobacillales	-0.089804663	-0.800390865	0.601662214	0.348265173	-0.099456639	-0.934993174	-0.448108309	-0.428827696	0.067711264
k__Bacteria.p__Firmicutes.c__Clostridia.o__Clostridiales.f__Ruminococcaceae.g__	-0.453047789	0.838428316	-0.659613148	0.097782062	-0.883284867	-0.844802425	0.813010473	0.339915015	0.579167063
k__Bacteria.p__Actinobacteria.c__Actinobacteria.o__Bifidobacteriales	-0.482465188	-0.05291808	-0.423481626	-0.382187182	-0.612617697	-0.635711342	-0.967425193	-0.148272979	-0.021277031
k__Bacteria.p__Actinobacteria.c__Actinobacteria.o__Bifidobacteriales.f__Bifidobacteriaceae	-0.482465188	-0.05291808	-0.423481626	-0.382187182	-0.612617697	-0.635711342	-0.967425193	-0.148272979	-0.021277031
k__Bacteria.p__Bacteroidetes.c__Bacteroidia.o__Bacteroidales.Other	-0.009403198	-0.084844094	-0.014938936	-0.001664784	-0.246177943	-0.22053931	-0.00140551	-0.011081697	-0.266705168
k__Bacteria.p__Actinobacteria.c__Actinobacteria.o__Bifidobacteriales.f__Bifidobacteriaceae.g__Bifidobacterium	-0.482465188	-0.05291808	-0.423481626	-0.382187182	-0.612617697	-0.635711342	-0.967425193	-0.148272979	-0.021277031
k__Bacteria.p__Actinobacteria.c__Coriobacteriia.o__Coriobacteriales.f__Coriobacteriaceae.g__Eggerthella	-0.008545938	-0.774995729	-0.273771482	-0.00122006	-0.423511214	-0.986967451	-0.050932565	-0.08781822	0.20850597
k__Bacteria.p__Bacteroidetes.c__Bacteroidia.o__Bacteroidales.Other.Other	-0.009403198	-0.084844094	-0.014938936	-0.001664784	-0.246177943	-0.22053931	-0.00140551	-0.011081697	-0.266705168
k__Bacteria.p__Firmicutes.c__Clostridia.o__Clostridiales.f__Lachnospiraceae.g__Clostridium	-0.671075358	-0.83183939	0.4524169	-0.263189339	-1	0.819114175	0.774981754	-0.66510135	0.545616432
k__Bacteria.p__Firmicutes.c__Clostridia.o__Clostridiales.f__Ruminococcaceae.g__Faecalibacterium	0.028618851	0.52943928	0.252863301	0.364649255	0.369001172	-0.947904556	0.229894089	0.100656315	-0.12875715
k__Bacteria.p__Firmicutes.c__Clostridia.o__Clostridiales.f__Veillonellaceae	-0.000923616	-0.806145492	-0.91530937	-0.986947106	-0.812490761	-0.408684074	-0.882934458	-0.226000931	0.799824989
k__Bacteria.p__Firmicutes.c__Clostridia.o__Clostridiales.f__Lachnospiraceae.g__Dorea	0.380550586	-0.450862059	-0.329418559	0.844070077	-0.774241993	-0.358662867	-0.533356033	-0.718382631	-0.407834016
k__Bacteria.p__Firmicutes.Other	-0.054622462	0.260491146	0.459769761	-0.105655498	-0.177996432	0.479957119	0.993446346	-0.711662227	0.045070238
k__Bacteria.p__Firmicutes.Other.Other	-0.054622462	0.260491146	0.459769761	-0.105655498	-0.177996432	0.479957119	0.993446346	-0.711662227	0.045070238
k__Bacteria.p__Firmicutes.Other.Other.Other	-0.054622462	0.260491146	0.459769761	-0.105655498	-0.177996432	0.479957119	0.993446346	-0.711662227	0.045070238
k__Bacteria.p__Firmicutes.Other.Other.Other.Other	-0.054622462	0.260491146	0.459769761	-0.105655498	-0.177996432	0.479957119	0.993446346	-0.711662227	0.045070238
k__Bacteria.p__Firmicutes.c__Bacilli.o__Lactobacillales.f__Streptococcaceae	-0.036491962	-0.56992994	-0.921287077	0.46365773	-0.229307364	-0.51005596	-0.483962124	-0.387238052	0.269880176
k__Bacteria.p__Actinobacteria.c__Coriobacteriia.o__Coriobacteriales.f__Coriobacteriaceae.Other	-0.704867574	0.140506222	-0.36505124	0.252398312	-0.817665696	-0.542296271	0.798511647	0.108327873	0.113900222
k__Bacteria.p__Firmicutes.c__Bacilli.o__Lactobacillales.f__Streptococcaceae.g__Streptococcus	-0.036491962	-0.56992994	-0.921287077	0.46365773	-0.229307364	-0.51005596	-0.483962124	-0.387238052	0.269880176
k__Bacteria.p__Firmicutes.c__Clostridia.o__Clostridiales.f__Ruminococcaceae.g__Gemmiger	-0.317706829	0.574428945	0.875318975	0.261402866	-0.823568326	-0.797947413	-0.655628318	0.268452201	-0.685751581
Dada_1.k__Bacteria.p__Actinobacteria.c__Coriobacteriia.o__Coriobacteriales.f__Coriobacteriaceae.g__Collinsella.s__	0.790161754	-0.733139918	-0.360309033	0.35597362	-0.147918808	-0.946956517	-0.419844531	-0.275956079	0.752006735
Dada_4.k__Bacteria.p__Actinobacteria.c__Coriobacteriia.o__Coriobacteriales.f__Coriobacteriaceae.g__Collinsella.s__	0.815887203	-0.835309081	-0.310310762	0.338906532	-0.106693522	0.946956517	-0.419844531	-0.234343077	0.828826619
k__Bacteria.Other	-0.015885464	0.511209351	-0.434380318	0.543810827	0.665440958	0.051657279	-0.364677707	-1	0.086708411
k__Bacteria.Other.Other	-0.015885464	0.511209351	-0.434380318	0.543810827	0.665440958	0.051657279	-0.364677707	-1	0.086708411
k__Bacteria.p__Proteobacteria.c__Gammaproteobacteria	-0.009941734	0.790155518	-0.287117165	0.796566842	-0.005205146	-0.933723133	-0.175234897	-0.136583255	0.334737895
k__Bacteria.Other.Other.Other	-0.015885464	0.511209351	-0.434380318	0.543810827	0.665440958	0.051657279	-0.364677707	-1	0.086708411
k__Bacteria.Other.Other.Other.Other	-0.015885464	0.511209351	-0.434380318	0.543810827	0.665440958	0.051657279	-0.364677707	-1	0.086708411
k__Bacteria.Other.Other.Other.Other.Other	-0.015885464	0.511209351	-0.434380318	0.543810827	0.665440958	0.051657279	-0.364677707	-1	0.086708411
k__Bacteria.p__Actinobacteria.c__Coriobacteriia.o__Coriobacteriales.f__Coriobacteriaceae.g__Collinsella	0.726896376	-0.671485088	-0.3516422	0.459230377	-0.117972441	0.85483311	-0.429493783	-0.150224895	0.739416829
Dada_6.k__Bacteria.p__Firmicutes.c__Clostridia.o__Clostridiales.f__Lachnospiraceae.g__Ruminococcus.s__lactaris	0.242383287	-0.296565623	-0.376053259	-0.058014399	-0.413165728	0.341097693	-0.195519302	-0.408372773	-0.514834691
k__Bacteria.p__Proteobacteria.c__Gammaproteobacteria.o__Enterobacteriales	-0.010113886	0.616345247	-0.413137521	0.782878184	-0.010612364	0.854233881	-0.266698119	-0.225929144	0.270354039
k__Bacteria.p__Firmicutes.c__Clostridia.o__Clostridiales.f__Peptostreptococcaceae	0.432492322	-0.389726692	-0.462414948	0.782878184	0.973354459	-0.575815108	0.770065595	-0.782865401	-0.001597097
k__Bacteria.p__Proteobacteria.c__Gammaproteobacteria.o__Enterobacteriales.f__Enterobacteriaceae	-0.010113886	0.616345247	-0.413137521	0.782878184	-0.010612364	0.854233881	-0.266698119	-0.225929144	0.270354039
k__Bacteria.p__Firmicutes.c__Clostridia.o__Clostridiales.f__Peptostreptococcaceae.g__Clostridium	0.442348663	-0.328558412	-0.413137521	0.821616348	0.986675372	-0.610469943	0.782865401	-0.719521753	-0.001343423
k__Bacteria.p__Proteobacteria.c__Gammaproteobacteria.o__Enterobacteriales.f__Enterobacteriaceae.Other	-0.010113886	0.616345247	-0.413137521	0.782878184	-0.010612364	0.854233881	-0.266698119	-0.225929144	0.270354039
Dada_2.k__Bacteria.p__Actinobacteria.c__Coriobacteriia.o__Coriobacteriales.f__Coriobacteriaceae.g__Collinsella.s__	0.64450134	-0.523727671	-0.435239915	0.973229284	-0.088560861	0.705769769	-0.330427921	-0.075288551	0.730873778
Dada_3.k__Bacteria.p__Firmicutes.c__Clostridia.o__Clostridiales.f__Lachnospiraceae.g__Ruminococcus.s__lactaris	0.286673363	-0.47059831	-0.27541322	-0.347404512	0.42545773	0.906497151	-0.512835923	-1	-0.071276665
k__Bacteria.p__Firmicutes.c__Bacilli.o__Bacillales	0.001391814	0.551401149	0.781883609	-0.529204864	-0.64450134	0.396785949	0.614678598	0.290441599	-0.109074636
k__Bacteria.p__Bacteroidetes.c__Bacteroidia.o__Bacteroidales.f__Porphyromonadaceae	-0.105416071	-0.190609511	-0.360457239	-0.814279443	0.8865911	0.860147072	-0.893211455	-0.814268521	-0.899858353
k__Bacteria.p__Firmicutes.c__Clostridia.o__Clostridiales.f__Lachnospiraceae.g__Roseburia	0.236849308	-0.545809434	-0.769029249	-0.512861171	0.199292138	-0.430318733	0.693333737	0.373814339	-0.03671234
k__Bacteria.p__Firmicutes.c__Clostridia.o__Clostridiales.f__Ruminococcaceae.g__Butyricicoccus	0.193481174	0.275427598	0.369337797	0.002389165	0.318117949	-0.038238019	0.096665054	0.153776562	-0.496797333
k__Bacteria.p__Firmicutes.c__Clostridia.o__Clostridiales.f__Veillonellaceae.g__Succinispira	-0.006082172	-0.626543947	-0.369337797	-0.724565786	0.128895261	-0.496771553	-0.56832017	-0.257355246	0.243565945
Dada_5.k__Bacteria.p__Actinobacteria.c__Coriobacteriia.o__Coriobacteriales.f__Coriobacteriaceae.g__Collinsella.s__	0.494561212	-0.946210787	-0.704311327	0.577804928	-0.23441935	0.806788429	-0.698051464	-0.110941141	0.549344737
k__Bacteria.p__Verrucomicrobia	-0.057772157	-0.437832744	-0.075163286	-0.077976868	0.8594355	0.075163286	-0.174516862	-0.723176148	0.899340222
k__Bacteria.p__Verrucomicrobia.c__Verrucomicrobiae	-0.057772157	-0.437832744	-0.075163286	-0.077976868	0.8594355	0.075163286	-0.174516862	-0.723176148	0.899340222
k__Bacteria.p__Verrucomicrobia.c__Verrucomicrobiae.o__Verrucomicrobiales	-0.057772157	-0.437832744	-0.075163286	-0.077976868	0.8594355	0.075163286	-0.174516862	-0.723176148	0.899340222
k__Bacteria.p__Bacteroidetes.c__Bacteroidia.o__Bacteroidales.f__.Barnesiellaceae.	0.26203778	0.161537884	0.691829077	0.25845336	0.083851519	0.710582702	0.505241348	0.015499866	0.912703711
k__Bacteria.p__Verrucomicrobia.c__Verrucomicrobiae.o__Verrucomicrobiales.f__Verrucomicrobiaceae	-0.057772157	-0.437832744	-0.075163286	-0.077976868	0.8594355	0.075163286	-0.174516862	-0.723176148	0.899340222
k__Bacteria.p__Bacteroidetes.c__Bacteroidia.o__Bacteroidales.f__.Barnesiellaceae..g__Barnesiella	0.26203778	0.161537884	0.691829077	0.25845336	0.083851519	0.710582702	0.505241348	0.015499866	0.912703711
Dada_39.k__Bacteria.p__Actinobacteria.c__Coriobacteriia.o__Coriobacteriales.f__Coriobacteriaceae.g__Collinsella.s__	-0.945902265	-0.628761817	-0.149310401	0.593091139	-0.110811346	-0.966171117	-0.410623877	-0.218714654	0.918931118
Dada_44.k__Bacteria.p__Firmicutes.c__Clostridia.o__Clostridiales.f__Ruminococcaceae.g__Faecalibacterium.s__	0.228435245	0.979699374	0.377697157	0.025697754	-0.721667722	0.502798724	0.08355583	0.581388079	-0.096430508
Dada_15.k__Bacteria.p__Firmicutes.c__Clostridia.o__Clostridiales.f__Lachnospiraceae.g__Ruminococcus.s__lactaris	0.538829162	-0.158993746	-0.626559919	-0.437283157	-0.330507528	0.473338856	-0.871151331	0.837666315	-0.719963468
k__Bacteria.p__Firmicutes.c__Erysipelotrichi.o__Erysipelotrichales.f__Erysipelotrichaceae.g__Clostridium	-0.356592878	-0.510998863	-0.745648103	-0.001731679	-0.267142792	-1	-0.118239522	-0.185775609	-0.851041507
Dada_12.k__Bacteria.p__Firmicutes.c__Clostridia.o__Clostridiales.f__Ruminococcaceae.g__Gemmiger.s__formicilis	0.965450587	0.17654787	0.86925433	0.077178856	-0.722453678	-0.567465416	0.722430771	0.323317892	-0.504741444
Dada_17.k__Bacteria.p__Actinobacteria.c__Actinobacteria.o__Bifidobacteriales.f__Bifidobacteriaceae.g__Bifidobacterium.s__breve	-0.000662746	-0.014564717	-0.01421599	-0.018888209	-0.420441554	0.176535455	-0.096226025	-0.112862206	-0.163095216
k__Bacteria.p__Proteobacteria.c__Betaproteobacteria	-0.924082438	-0.591185394	-0.294510303	0.555795337	0.147976876	0.173778337	-0.848837765	0.043524251	0.965450587
k__Bacteria.p__Firmicutes.c__Bacilli.o__Bacillales.Other	0.033802014	0.561621251	0.855635592	-0.271232925	-0.179350006	0.202830584	0.677513945	0.555772656	-0.278866039
k__Bacteria.p__Firmicutes.c__Bacilli.o__Bacillales.Other.Other	0.033802014	0.561621251	0.855635592	-0.271232925	-0.179350006	0.202830584	0.677513945	0.555772656	-0.278866039
Dada_7.k__Bacteria.p__Proteobacteria.c__Gammaproteobacteria.o__Enterobacteriales.f__Enterobacteriaceae	-0.00250269	0.31922539	-0.606193389	-0.759753032	-0.019205043	0.88191809	-0.431632746	-0.187033518	0.382279441
k__Bacteria.p__Actinobacteria.c__Actinobacteria.o__Actinomycetales	-0.902646727	0.468332415	-0.164751128	0.88882068	0.930377092	-0.159503448	-0.820281883	-0.854410812	0.576045298
k__Bacteria.p__Firmicutes.c__Clostridia.o__Clostridiales.f__Clostridiaceae	-0.972121076	-0.618466086	-0.793224205	0.452407837	-0.687754777	-0.588006361	-0.490014916	0.436767733	-0.972121076
k__Bacteria.p__Bacteroidetes.c__Bacteroidia.o__Bacteroidales.f__Porphyromonadaceae.g__Macellibacteroides	-0.178462451	-0.377524753	-0.71363649	0.668558541	0.421508024	-0.916494477	0.930371167	0.930371167	0.98605841
k__Bacteria.p__Firmicutes.c__Clostridia.o__Clostridiales.f__Clostridiaceae.g__Clostridium	-0.972121076	-0.618466086	-0.793224205	0.452407837	-0.687754777	-0.588006361	-0.490014916	0.436767733	-0.972121076
k__Bacteria.p__Verrucomicrobia.c__Verrucomicrobiae.o__Verrucomicrobiales.f__Verrucomicrobiaceae.g__Akkermansia	-0.227928711	-0.720172058	-0.278606667	-0.612321285	0.506680658	0.184143188	-0.600092732	0.993028344	0.888823821
Dada_31.k__Bacteria.p__Firmicutes.c__Clostridia.o__Clostridiales.f__Ruminococcaceae.g__Gemmiger.s__formicilis	-0.915717814	0.039887742	0.519685024	0.327605514	0.929724825	-0.140785102	0.724242839	0.730865684	0.750873491

Supplementary Figure 5A: Time-point: T2_*Heatmap*

	Spearman's rho								
	tryptophan	n_acetyltrypto	indolelactic	indoleacetic	indolepropion	indoleacetami	indolealdehyd	x3_methylindo	indoxyl_s
k__Bacteria.p__Actinobacteria	-0.079215686	-0.065908204	-0.110675073	-0.010196078	-0.116123979	-0.016844664	-0.139662624	-0.087877606	-0.146666667
k__Bacteria.p__Firmicutes	-0.104313725	-0.012553944	0.063579297	-0.035294118	-0.186739912	0.000802127	-0.053354261	-0.138093381	0.094901961
k__Bacteria.p__Firmicutes.c__Clostridia	0.081581016	0.014838732	-0.009071675	0.002472152	0.224229732	-0.049723211	0.057706181	0.140143583	-0.005768355
k__Bacteria.p__Firmicutes.c__Clostridia.o__Clostridiales	0.099710131	0.026379968	-0.014019862	-0.055211395	0.247312204	-0.017698092	0.056057433	0.12530485	0.025545571
k__Bacteria.p__Bacteroidetes	-0.123137255	-0.004707729	-0.072998453	-0.160784314	-0.16947824	-0.089036081	-0.139662624	-0.11141625	0.057254902
k__Bacteria.p__Bacteroidetes.c__Bacteroidia	-0.12	0.001569243	-0.066719016	-0.157647059	-0.172616726	-0.085827573	-0.133385652	-0.117693222	0.060392157
k__Bacteria.p__Bacteroidetes.c__Bacteroidia.o__Bacteroidales	-0.12	0.001569243	-0.066719016	-0.157647059	-0.172616726	-0.085827573	-0.133385652	-0.117693222	0.060392157
k__Bacteria.p__Firmicutes.c__Clostridia.o__Clostridiales.f__Lachnospiraceae	0.094901961	0.020400158	-0.046310846	-0.036862745	-0.075323662	-0.087431827	-0.112985493	0.001569243	0.021176471
k__Bacteria.p__Firmicutes.c__Clostridia.o__Clostridiales.f__Ruminococcaceae	-0.043137255	-0.047077289	-0.074568312	-0.049411765	0.155355053	0.267108242	0.054923504	0.056492747	0.030588235
k__Bacteria.p__Actinobacteria.c__Coriobacteriia	0.031384859	0.035321821	-0.017275227	-0.032954102	-0.162480377	-0.088268576	-0.022762951	-0.083987441	0.01569243
k__Bacteria.p__Actinobacteria.c__Coriobacteriia.o__Coriobacteriales	0.031384859	0.035321821	-0.017275227	-0.032954102	-0.162480377	-0.088268576	-0.022762951	-0.083987441	0.01569243
k__Bacteria.p__Actinobacteria.c__Coriobacteriia.o__Coriobacteriales.f__Coriobacteriaceae	0.031384859	0.035321821	-0.017275227	-0.032954102	-0.162480377	-0.088268576	-0.022762951	-0.083987441	0.01569243
k__Bacteria.p__Firmicutes.c__Clostridia.o__Clostridiales.f__Ruminococcaceae.Other	0.020400158	-0.184458399	-0.188457023	-0.032954102	0.109105181	0.222276322	-0.052590267	-0.043171115	0.091016092
k__Bacteria.p__Bacteroidetes.c__Bacteroidia.o__Bacteroidales.f__Bacteroidaceae	-0.010993333	0.041633948	0.033005896	-0.02041619	-0.117046383	-0.126885407	-0.043205041	-0.220738481	0.007852381
k__Bacteria.p__Bacteroidetes.c__Bacteroidia.o__Bacteroidales.f__Bacteroidaceae.g__Bacteroides	-0.010993333	0.041633948	0.033005896	-0.02041619	-0.117046383	-0.126885407	-0.043205041	-0.220738481	0.007852381
k__Bacteria.p__Firmicutes.c__Clostridia.o__Clostridiales.f__Lachnospiraceae.Other	-0.073812379	0.06205815	0.053438118	0.015704762	-0.183032264	-0.118854685	-0.112333106	-0.007069916	0.179034282
k__Bacteria.p__Firmicutes.c__Clostridia.o__Clostridiales.f__Lachnospiraceae.g__Ruminococcus	0.034550475	-0.113904199	-0.12259333	-0.058107618	-0.01649647	0.004818433	-0.124901845	-0.06205815	0.006281905
k__Bacteria.p__Firmicutes.c__Clostridia.o__Clostridiales.Other	0.101415376	0.053480245	0.040125935	-0.032232794	0.173024322	-0.001608042	0.047188451	-0.066063832	0.040094451
k__Bacteria.p__Firmicutes.c__Clostridia.o__Clostridiales.Other.Other	0.101415376	0.053480245	0.040125935	-0.032232794	0.173024322	-0.001608042	0.047188451	-0.066063832	0.040094451
k__Bacteria.p__Firmicutes.c__Bacilli	-0.059055576	-0.033084099	0.057525896	0.03385853	-0.195353728	0.017716404	-0.050413865	-0.105554031	0.059055576
k__Bacteria.p__Proteobacteria	-0.339256432	-0.187058893	-0.072642094	-0.118345267	-0.176009	-0.097633362	-0.187058893	-0.089188417	0.102565898
k__Bacteria.p__Actinobacteria.c__Actinobacteria	-0.066037002	-0.109839543	-0.09953141	0.023073169	-0.041388813	0.056958761	-0.133717705	-0.054919772	-0.199702258
k__Bacteria.p__Firmicutes.c__Bacilli.o__Lactobacillales	-0.067185557	-0.034010851	0.025113105	0.057471983	-0.226739008	0.001655698	-0.116608633	-0.098793425	0.078518061
k__Bacteria.p__Firmicutes.c__Bacilli.o__Lactobacillales.f__Streptococcaceae	-0.127085934	-0.080978217	-0.038074708	0.016998755	-0.263988988	-0.018212676	-0.157097741	-0.118228197	0.063947699
k__Bacteria.p__Firmicutes.c__Clostridia.o__Clostridiales.f__	0.075280203	-0.152239048	-0.013771703	-0.025093401	-0.006478257	-0.079473494	-0.014576079	0.090695603	0.00890411
k__Bacteria.p__Firmicutes.c__Bacilli.o__Lactobacillales.f__Streptococcaceae.g__Streptococcus	-0.127085934	-0.080978217	-0.038074708	0.016998755	-0.263988988	-0.018212676	-0.157097741	-0.118228197	0.063947699
k__Bacteria.p__Firmicutes.c__Clostridia.o__Clostridiales.f__.g__	0.075280203	-0.152239048	-0.013771703	-0.025093401	-0.006478257	-0.079473494	-0.014576079	0.090695603	0.00890411
k__Bacteria.p__Firmicutes.c__Clostridia.o__Clostridiales.f__Ruminococcaceae.g__Clostridium	-0.152988799	0.059923881	-0.033214107	-0.078518061	-0.071260831	0.130800125	-0.089076039	-0.158717306	0.091469494
k__Bacteria.p__Actinobacteria.c__Actinobacteria.o__Bifidobacteriales	-0.053717154	-0.101776954	-0.084711678	0.032555851	-0.023612253	0.071584789	-0.12294656	-0.076536269	-0.183940556
k__Bacteria.p__Actinobacteria.c__Actinobacteria.o__Bifidobacteriales.f__Bifidobacteriaceae	-0.053717154	-0.101776954	-0.084711678	0.032555851	-0.023612253	0.071584789	-0.12294656	-0.076536269	-0.183940556
k__Bacteria.p__Bacteroidetes.c__Bacteroidia.o__Bacteroidales.f__Rikenellaceae	-0.185568349	-0.000814216	0.004887212	-0.200218482	-0.088749504	0.05909907	-0.069208329	-0.008956372	0.144873535
k__Bacteria.p__Actinobacteria.c__Actinobacteria.o__Bifidobacteriales.f__Bifidobacteriaceae.g__Bifidobacterium	-0.053717154	-0.101776954	-0.084711678	0.032555851	-0.023612253	0.071584789	-0.12294656	-0.076536269	-0.183940556
k__Bacteria.p__Firmicutes.c__Clostridia.o__Clostridiales.f__Ruminococcaceae.g__	-0.032555851	-0.048038722	0	0.066739494	-0.026869116	0.061596214	-0.105033816	0.065951466	0.162779253
k__Bacteria.p__Firmicutes.c__Clostridia.o__Clostridiales.f__Ruminococcaceae.g__Faecalibacterium	0.306212968	0.136785111	0.136019431	0.108075165	0.179376882	0.116391165	0.217054218	0.210501637	-0.070412608
k__Bacteria.p__Firmicutes.c__Erysipelotrichi	0.168930387	0.126953598	0.037111398	-0.122783549	0.080788653	-0.166867725	0.079964279	0.176416039	-0.002472152
k__Bacteria.p__Firmicutes.c__Erysipelotrichi.o__Erysipelotrichales	0.168930387	0.126953598	0.037111398	-0.122783549	0.080788653	-0.166867725	0.079964279	0.176416039	-0.002472152
k__Bacteria.p__Firmicutes.c__Erysipelotrichi.o__Erysipelotrichales.f__Erysipelotrichaceae	0.168930387	0.126953598	0.037111398	-0.122783549	0.080788653	-0.166867725	0.079964279	0.176416039	-0.002472152
k__Bacteria.p__Bacteroidetes.c__Bacteroidia.o__Bacteroidales.f__Rikenellaceae.g__Alistipes	-0.124431651	-0.02473122	0.020617444	-0.137616461	-0.041218701	0.033710652	-0.059354929	-0.00824374	0.127727853
k__Bacteria.p__Firmicutes.c__Clostridia.o__Clostridiales.f__Lachnospiraceae.g__Dorea	0.063451901	0.082437401	-0.023916235	0.08652532	0.004946244	0.080062797	-0.028028717	-0.051111189	-0.08652532
k__Bacteria.p__Proteobacteria.c__Deltaproteobacteria	-0.005808729	0.092976126	0.122909556	-0.067215288	-0.172669948	-0.271572796	-0.122861309	-0.064751231	0.014106912
k__Bacteria.p__Proteobacteria.c__Deltaproteobacteria.o__Desulfovibrionales	-0.005808729	0.092976126	0.122909556	-0.067215288	-0.172669948	-0.271572796	-0.122861309	-0.064751231	0.014106912
k__Bacteria.p__Firmicutes.c__Clostridia.o__Clostridiales.f__Peptostreptococcaceae	-0.020745459	-0.033205759	0.057302428	0.193347681	-0.028224895	-0.095899144	0.083014398	0.122861309	-0.156835672
k__Bacteria.p__Proteobacteria.c__Deltaproteobacteria.o__Desulfovibrionales.f__Desulfovibrionaceae	-0.005808729	0.092976126	0.122909556	-0.067215288	-0.172669948	-0.271572796	-0.122861309	-0.064751231	0.014106912
k__Bacteria.p__Firmicutes.c__Clostridia.o__Clostridiales.f__Peptostreptococcaceae.g__Clostridium	-0.029043643	-0.011622016	0.068929008	0.214922958	-0.053129215	-0.090807154	0.074712958	0.104598142	-0.155176035
k__Bacteria.p__Proteobacteria.c__Deltaproteobacteria.o__Desulfovibrionales.f__Desulfovibrionaceae.g__Desulfovibri	-0.005808729	0.092976126	0.122909556	-0.067215288	-0.172669948	-0.271572796	-0.122861309	-0.064751231	0.014106912
k__Bacteria.p__Actinobacteria.c__Coriobacteriia.o__Coriobacteriales.f__Coriobacteriaceae.g__Eggerthella	-0.175577061	-0.046002512	-0.082837039	-0.272562484	-0.025928689	0.061565047	-0.122952168	-0.226666923	-0.043476225
k__Bacteria.p__Proteobacteria.c__Gammaproteobacteria	-0.405553746	-0.205833369	-0.111465951	-0.043361092	-0.128433218	-0.043476371	-0.193925654	-0.062090231	0.080770662
k__Bacteria.p__Actinobacteria.c__Coriobacteriia.o__Coriobacteriales.f__Coriobacteriaceae.g__Collinsella	0.189598502	0.06123968	0.016166817	0.153889367	-0.171811325	-0.131298639	0.022114329	0.034022044	0.029757612
k__Bacteria.p__Proteobacteria.c__Gammaproteobacteria.o__Enterobacteriales	-0.390464451	-0.187153079	-0.093613286	-0.024886745	-0.139935559	-0.059680473	-0.178568075	-0.040349517	0.098688817
k__Bacteria.p__Bacteroidetes.c__Bacteroidia.o__Bacteroidales.Other	-0.138164344	-0.030906013	-0.110790036	-0.27203787	-0.185436078	0.000877654	-0.227502596	-0.104737044	0.119284744
k__Bacteria.p__Firmicutes.c__Clostridia.o__Clostridiales.f__Veillonellaceae	-0.167341907	-0.013736006	-0.033496462	0.076376563	-0.16569057	-0.085132439	-0.149379063	-0.115039048	-0.030035727
k__Bacteria.p__Proteobacteria.c__Gammaproteobacteria.o__Enterobacteriales.f__Enterobacteriaceae	-0.390464451	-0.187153079	-0.093613286	-0.024886745	-0.139935559	-0.059680473	-0.178568075	-0.040349517	0.098688817
k__Bacteria.p__Bacteroidetes.c__Bacteroidia.o__Bacteroidales.Other.Other	-0.138164344	-0.030906013	-0.110790036	-0.27203787	-0.185436078	0.000877654	-0.227502596	-0.104737044	0.119284744
k__Bacteria.p__Proteobacteria.c__Gammaproteobacteria.o__Enterobacteriales.f__Enterobacteriaceae.Other	-0.387031796	-0.194021082	-0.100483986	-0.024886745	-0.136501557	-0.052659241	-0.178568075	-0.036915516	0.091823508
Dada_1.k__Bacteria.p__Actinobacteria.c__Coriobacteriia.o__Coriobacteriales.f__Coriobacteriaceae.g__Collinsella.s__	0.100543187	0.051158411	-0.03122756	0.097076181	-0.21243747	-0.127646935	-0.016474743	0.073702796	-0.038137071
Dada_4.k__Bacteria.p__Actinobacteria.c__Coriobacteriia.o__Coriobacteriales.f__Coriobacteriaceae.g__Collinsella.s__	0.150814781	0.066766062	0.017348644	0.147347774	-0.219374204	-0.109918194	0.061563512	0.061563512	-0.019068535
k__Bacteria.p__Firmicutes.c__Bacilli.o__Bacillales	0.057593772	-0.03722904	0.009754292	-0.085947628	0.039001851	-0.005437092	0.179940358	0.145370535	-0.055821656
k__Bacteria.Other	-0.065473485	-0.170477308	-0.072705708	0.164132162	0.012561486	0.050449693	-0.010766988	0.054732188	0.052916926
k__Bacteria.p__Verrucomicrobia	-0.131843868	-0.165093814	-0.209141111	-0.225121162	0.059218433	0.198129704	-0.208161766	-0.056526686	0.040360368
k__Bacteria.Other.Other	-0.065473485	-0.170477308	-0.072705708	0.164132162	0.012561486	0.050449693	-0.010766988	0.054732188	0.052916926
k__Bacteria.p__Verrucomicrobia.c__Verrucomicrobiae	-0.131843868	-0.165093814	-0.209141111	-0.225121162	0.059218433	0.198129704	-0.208161766	-0.056526686	0.040360368
k__Bacteria.Other.Other.Other	-0.065473485	-0.170477308	-0.072705708	0.164132162	0.012561486	0.050449693	-0.010766988	0.054732188	0.052916926
k__Bacteria.p__Verrucomicrobia.c__Verrucomicrobiae.o__Verrucomicrobiales	-0.131843868	-0.165093814	-0.209141111	-0.225121162	0.059218433	0.198129704	-0.208161766	-0.056526686	0.040360368
k__Bacteria.Other.Other.Other.Other	-0.065473485	-0.170477308	-0.072705708	0.164132162	0.012561486	0.050449693	-0.010766988	0.054732188	0.052916926
k__Bacteria.p__Bacteroidetes.c__Bacteroidia.o__Bacteroidales.f__.Barnesiellaceae.	0.024216221	0.078957911	-0.045777668	-0.135431456	-0.001794498	-0.175198025	0.051143192	0.00897249	-0.018834838
k__Bacteria.p__Verrucomicrobia.c__Verrucomicrobiae.o__Verrucomicrobiales.f__Verrucomicrobiaceae	-0.131843868	-0.165093814	-0.209141111	-0.225121162	0.059218433	0.198129704	-0.208161766	-0.056526686	0.040360368
k__Bacteria.Other.Other.Other.Other.Other	-0.065473485	-0.170477308	-0.072705708	0.164132162	0.012561486	0.050449693	-0.010766988	0.054732188	0.052916926
k__Bacteria.p__Bacteroidetes.c__Bacteroidia.o__Bacteroidales.f__.Barnesiellaceae..g__Barnesiella	0.024216221	0.078957911	-0.045777668	-0.135431456	-0.001794498	-0.175198025	0.051143192	0.00897249	-0.018834838
k__Bacteria.p__Firmicutes.c__Clostridia.o__Clostridiales.f__Lachnospiraceae.g__Clostridium	0.081775953	0.080899063	0.0491041	-0.07995871	0.002726935	-0.254616713	0.018179565	0.033632195	0.043613842
Dada_6.k__Bacteria.p__Firmicutes.c__Clostridia.o__Clostridiales.f__Lachnospiraceae.g__Ruminococcus.s__lactaris	-0.029482133	0.076499288	-0.044257925	-0.009213167	-0.163137036	-0.158296548	-0.129034944	0.017511885	0.020268966
k__Bacteria.p__Firmicutes.c__Clostridia.o__Clostridiales.f__Ruminococcaceae.g__Gemmiger	0.001842633	0.101384598	0.043335885	0.023954233	0.006451747	0.151700858	0.066360828	0.135486691	-0.114243265
k__Bacteria.p__Firmicutes.c__Clostridia.o__Clostridiales.f__Veillonellaceae.g__Succinispira	-0.136354865	-0.004608391	-0.024895083	0.009213167	-0.062674115	0.095166377	-0.109679702	-0.139173403	0.084761132

Supplementary Figure 5B: Time-point: T2_*Merged Spearman's rho and p-values matrix*

	Merge rho+P								
	tryptophan	n_acetyltrypto	indolelactic	indoleacetic	indolepropion	indoleacetami	indolealdehyd	x3_methylindc	indoxyl_s
k__Bacteria.p__Actinobacteria	-0.412020416	-0.495056708	-0.252081872	-0.915908655	-0.229314367	-0.863824248	-0.14823295	-0.362971432	-0.128799381
k__Bacteria.p__Firmicutes	-0.280029053	-0.896598339	0.510575076	-0.71473858	-0.053217721	0.993483707	-0.580723257	-0.152844402	0.325712013
k__Bacteria.p__Firmicutes.c__Clostridia	0.413635868	0.881835226	-0.927619501	0.980235954	0.024693564	-0.624242703	0.563225856	0.160362948	-0.953904846
k__Bacteria.p__Firmicutes.c__Clostridia.o__Clostridiales	0.317706829	0.791583834	-0.888352231	-0.580081465	0.013234877	-0.861588793	0.574428945	0.209402979	0.797959624
k__Bacteria.p__Bacteroidetes	-0.20224301	-0.961130583	-0.449999734	-0.095902127	-0.079352493	-0.364648565	-0.14823295	-0.248749514	0.553233748
k__Bacteria.p__Bacteroidetes.c__Bacteroidia	-0.213979031	0.987038969	-0.48992162	-0.102560637	-0.073946316	-0.382186498	-0.167333871	-0.223082695	0.531701833
k__Bacteria.p__Bacteroidetes.c__Bacteroidia.o__Bacteroidales	-0.213979031	0.987038969	-0.48992162	-0.102560637	-0.073946316	-0.382186498	-0.167333871	-0.223082695	0.531701833
k__Bacteria.p__Firmicutes.c__Clostridia.o__Clostridiales.f__Lachnospiraceae	0.325712013	0.83274334	-0.631766427	-0.702651318	-0.43553334	-0.37335378	-0.242147088	0.987038969	0.826416596
k__Bacteria.p__Firmicutes.c__Clostridia.o__Clostridiales.f__Ruminococcaceae	-0.655076076	-0.626011211	-0.440315256	-0.608861476	0.10778025	0.006535525	0.569645591	0.55866977	0.751420988
k__Bacteria.p__Actinobacteria.c__Coriobacteriia	0.745257391	0.71472059	-0.858168306	-0.732995089	-0.092683579	-0.368969938	-0.813774382	-0.384773894	0.87095183
k__Bacteria.p__Actinobacteria.c__Coriobacteriia.o__Coriobacteriales	0.745257391	0.71472059	-0.858168306	-0.732995089	-0.092683579	-0.368969938	-0.813774382	-0.384773894	0.87095183
k__Bacteria.p__Actinobacteria.c__Coriobacteriia.o__Coriobacteriales.f__Coriobacteriaceae	0.745257391	0.71472059	-0.858168306	-0.732995089	-0.092683579	-0.368969938	-0.813774382	-0.384773894	0.87095183
k__Bacteria.p__Firmicutes.c__Clostridia.o__Clostridiales.f__Ruminococcaceae.Other	0.83274334	-0.056282026	-0.05123294	-0.732995089	0.258871763	0.023676042	-0.586287315	-0.655054801	0.34608638
k__Bacteria.p__Bacteroidetes.c__Bacteroidia.o__Bacteroidales.f__Bacteroidaceae	-0.909456023	0.666800378	0.732955587	-0.832728843	-0.226127094	-0.196861036	-0.655026435	-0.022448485	0.935257502
k__Bacteria.p__Bacteroidetes.c__Bacteroidia.o__Bacteroidales.f__Bacteroidaceae.g__Bacteroides	-0.909456023	0.666800378	0.732955587	-0.832728843	-0.226127094	-0.196861036	-0.655026435	-0.022448485	0.935257502
k__Bacteria.p__Firmicutes.c__Clostridia.o__Clostridiales.f__Lachnospiraceae.Other	-0.445117451	0.521033297	0.58066506	0.870940576	-0.058388939	-0.226714259	-0.245375661	-0.94171767	0.064013166
k__Bacteria.p__Firmicutes.c__Clostridia.o__Clostridiales.f__Lachnospiraceae.g__Ruminococcus	0.720777017	-0.238835738	-0.205047455	-0.547762508	-0.864544053	0.96091285	-0.196485204	-0.521033297	0.948185506
k__Bacteria.p__Firmicutes.c__Clostridia.o__Clostridiales.Other	0.294608712	0.580615107	0.678596962	-0.73905435	0.073863785	-0.986964204	0.625912506	-0.494933414	0.678616897
k__Bacteria.p__Firmicutes.c__Clostridia.o__Clostridiales.Other.Other	0.294608712	0.580615107	0.678596962	-0.73905435	0.073863785	-0.986964204	0.625912506	-0.494933414	0.678616897
k__Bacteria.p__Firmicutes.c__Bacilli	-0.54219241	-0.732853854	0.552989421	0.726753573	-0.0438541	0.857331432	-0.602976761	-0.276149123	0.54219241
k__Bacteria.p__Proteobacteria	-0.000472608	-0.054001004	-0.454465213	-0.222665137	-0.069830676	-0.322602824	-0.054001004	-0.358253122	0.290568347
k__Bacteria.p__Actinobacteria.c__Actinobacteria	-0.498914523	-0.260877988	-0.308470246	0.81322923	-0.671816564	0.566362403	-0.171081998	-0.574007567	-0.040865711
k__Bacteria.p__Firmicutes.c__Bacilli.o__Lactobacillales	-0.496378847	-0.730690144	0.799444551	0.56066337	-0.021749317	0.9868517	-0.237944359	-0.317387357	0.426653839
k__Bacteria.p__Firmicutes.c__Bacilli.o__Lactobacillales.f__Streptococcaceae	-0.198219675	-0.412477261	-0.700091558	0.863360179	-0.007546654	-0.856150422	-0.111854769	-0.231489296	0.51736161
k__Bacteria.p__Firmicutes.c__Clostridia.o__Clostridiales.f__	0.445970477	-0.12338034	-0.889192127	-0.799456927	-0.947725462	-0.428929146	-0.882723523	0.358678019	0.928170579
k__Bacteria.p__Firmicutes.c__Bacilli.o__Lactobacillales.f__Streptococcaceae.g__Streptococcus	-0.198219675	-0.412477261	-0.700091558	0.863360179	-0.007546654	-0.856150422	-0.111854769	-0.231489296	0.51736161
k__Bacteria.p__Firmicutes.c__Clostridia.o__Clostridiales.f__.g__	0.445970477	-0.12338034	-0.889192127	-0.799456927	-0.947725462	-0.428929146	-0.882723523	0.358678019	0.928170579
k__Bacteria.p__Firmicutes.c__Clostridia.o__Clostridiales.f__Ruminococcaceae.g__Clostridium	-0.121409245	0.544208629	-0.736853669	-0.426653839	-0.470788353	0.192951927	-0.367324565	-0.10820751	0.354418313
k__Bacteria.p__Actinobacteria.c__Actinobacteria.o__Bifidobacteriales	-0.587773643	-0.304575574	-0.392984911	0.74251526	-0.811735601	0.477597483	-0.214894718	-0.440081066	-0.063430414
k__Bacteria.p__Actinobacteria.c__Actinobacteria.o__Bifidobacteriales.f__Bifidobacteriaceae	-0.587773643	-0.304575574	-0.392984911	0.74251526	-0.811735601	0.477597483	-0.214894718	-0.440081066	-0.063430414
k__Bacteria.p__Bacteroidetes.c__Bacteroidia.o__Bacteroidales.f__Rikenellaceae	-0.061125215	-0.993446756	0.960694775	-0.043339368	-0.370649824	0.557670713	-0.48509212	-0.928011459	0.143759512
k__Bacteria.p__Actinobacteria.c__Actinobacteria.o__Bifidobacteriales.f__Bifidobacteriaceae.g__Bifidobacterium	-0.587773643	-0.304575574	-0.392984911	0.74251526	-0.811735601	0.477597483	-0.214894718	-0.440081066	-0.063430414
k__Bacteria.p__Firmicutes.c__Clostridia.o__Clostridiales.f__Ruminococcaceae.g__	-0.74251526	-0.627968239	-1	0.500645242	-0.786359459	0.54114779	-0.289361154	0.505869769	0.100461251
k__Bacteria.p__Firmicutes.c__Clostridia.o__Clostridiales.f__Ruminococcaceae.g__Faecalibacterium	0.002073712	0.16910075	0.171654328	0.277093913	0.071345263	0.249844845	0.029106334	0.034330323	-0.478875215
k__Bacteria.p__Firmicutes.c__Erysipelotrichi	0.090488216	0.203470957	0.710176287	-0.218548507	0.418354484	-0.100198762	0.423119262	0.077194433	-0.980235954
k__Bacteria.p__Firmicutes.c__Erysipelotrichi.o__Erysipelotrichales	0.090488216	0.203470957	0.710176287	-0.218548507	0.418354484	-0.100198762	0.423119262	0.077194433	-0.980235954
k__Bacteria.p__Firmicutes.c__Erysipelotrichi.o__Erysipelotrichales.f__Erysipelotrichaceae	0.090488216	0.203470957	0.710176287	-0.218548507	0.418354484	-0.100198762	0.423119262	0.077194433	-0.980235954
k__Bacteria.p__Bacteroidetes.c__Bacteroidia.o__Bacteroidales.f__Rikenellaceae.g__Alistipes	-0.212429651	-0.804336684	0.836435364	-0.167883817	-0.679681621	0.739814392	-0.552129213	-0.934185882	0.200565147
k__Bacteria.p__Firmicutes.c__Clostridia.o__Clostridiales.f__Lachnospiraceae.g__Dorea	0.524878951	0.408920249	-0.810727185	0.385910105	0.960482813	0.430266625	-0.77888652	-0.60865587	-0.385910105
k__Bacteria.p__Proteobacteria.c__Deltaproteobacteria	-0.953753397	0.353442495	0.220110501	-0.502173651	-0.084833649	-0.007687302	-0.220124646	-0.518124699	0.887994284
k__Bacteria.p__Proteobacteria.c__Deltaproteobacteria.o__Desulfovibrionales	-0.953753397	0.353442495	0.220110501	-0.502173651	-0.084833649	-0.007687302	-0.220124646	-0.518124699	0.887994284
k__Bacteria.p__Firmicutes.c__Clostridia.o__Clostridiales.f__Peptostreptococcaceae	-0.835914989	-0.740338947	0.567531783	0.05356074	-0.778178273	-0.346573636	0.40738099	0.220124646	-0.11738646
k__Bacteria.p__Proteobacteria.c__Deltaproteobacteria.o__Desulfovibrionales.f__Desulfovibrionaceae	-0.953753397	0.353442495	0.220110501	-0.502173651	-0.084833649	-0.007687302	-0.220124646	-0.518124699	0.887994284
k__Bacteria.p__Firmicutes.c__Clostridia.o__Clostridiales.f__Peptostreptococcaceae.g__Clostridium	-0.771839135	-0.907659212	0.491650868	0.031890654	-0.595940521	-0.372780104	0.455872718	0.296520435	-0.121317151
k__Bacteria.p__Proteobacteria.c__Deltaproteobacteria.o__Desulfovibrionales.f__Desulfovibrionaceae.g__Desulfovibrio	-0.953753397	0.353442495	0.220110501	-0.502173651	-0.084833649	-0.007687302	-0.220124646	-0.518124699	0.887994284
k__Bacteria.p__Actinobacteria.c__Coriobacteriia.o__Coriobacteriales.f__Coriobacteriaceae.g__Eggerthella	-0.080761439	-0.64740205	-0.410338125	-0.006710094	-0.796566842	0.547200547	-0.221542527	-0.024220852	-0.665440958
k__Bacteria.p__Proteobacteria.c__Gammaproteobacteria	-6.28834E-05	-0.042327674	-0.271737108	-0.66875526	-0.205216035	-0.673226486	-0.05576987	-0.540245985	0.42545773
k__Bacteria.p__Actinobacteria.c__Coriobacteriia.o__Coriobacteriales.f__Coriobacteriaceae.g__Collinsella	0.061369264	0.545809434	0.873348034	0.128895261	-0.090132362	-0.202802916	0.827328064	0.737184485	0.76904269
k__Bacteria.p__Proteobacteria.c__Gammaproteobacteria.o__Enterobacteriales	-0.000124541	-0.06599664	-0.357964537	-0.80679968	-0.169249829	-0.564262795	-0.079410961	-0.691837761	0.332149242
k__Bacteria.p__Bacteroidetes.c__Bacteroidia.o__Bacteroidales.Other	-0.174555266	-0.76143519	-0.27663026	-0.007511847	-0.068518737	0.993235411	-0.025429903	-0.303547641	0.241122528
k__Bacteria.p__Firmicutes.c__Clostridia.o__Clostridiales.f__Veillonellaceae	-0.100086771	-0.892665755	-0.742226618	0.452931868	-0.103606447	-0.410854485	-0.142271653	-0.25845336	-0.767875736
k__Bacteria.p__Proteobacteria.c__Gammaproteobacteria.o__Enterobacteriales.f__Enterobacteriaceae	-0.000124541	-0.06599664	-0.357964537	-0.80679968	-0.169249829	-0.564262795	-0.079410961	-0.691837761	0.332149242
k__Bacteria.p__Bacteroidetes.c__Bacteroidia.o__Bacteroidales.Other.Other	-0.174555266	-0.76143519	-0.27663026	-0.007511847	-0.068518737	0.993235411	-0.025429903	-0.303547641	0.241122528
k__Bacteria.p__Proteobacteria.c__Gammaproteobacteria.o__Enterobacteriales.f__Enterobacteriaceae.Other	-0.000142794	-0.05666157	-0.323780668	-0.80679968	-0.179957701	-0.610965767	-0.079410961	-0.71688178	0.366881614
Dada_1.k__Bacteria.p__Actinobacteria.c__Coriobacteriia.o__Coriobacteriales.f__Coriobacteriaceae.g__Collinsella.s__	0.3251767	0.616769372	-0.760092988	0.342138258	-0.037702521	-0.219482803	-0.871969922	0.470931334	-0.709003928
Dada_4.k__Bacteria.p__Actinobacteria.c__Coriobacteriia.o__Coriobacteriales.f__Coriobacteriaceae.g__Collinsella.s__	0.139994007	0.513685978	0.865290344	0.149334366	-0.031878585	-0.290346294	0.547029411	0.547029411	-0.851976549
k__Bacteria.p__Firmicutes.c__Bacilli.o__Bacillales	0.576333422	-0.71806364	0.924662018	-0.404379842	0.705251826	-0.958646766	0.080973582	0.15859695	-0.588124292
k__Bacteria.Other	-0.527128017	-0.099761712	-0.482841219	0.112893198	0.903465346	0.631940025	-0.917202264	0.597183001	0.609270718
k__Bacteria.p__Verrucomicrobia	-0.202856503	-0.110929163	-0.043530277	-0.029674855	0.567476534	0.059950711	-0.044445503	-0.58521563	0.696659838
k__Bacteria.Other.Other	-0.527128017	-0.099761712	-0.482841219	0.112893198	0.903465346	0.631940025	-0.917202264	0.597183001	0.609270718
k__Bacteria.p__Verrucomicrobia.c__Verrucomicrobiae	-0.202856503	-0.110929163	-0.043530277	-0.029674855	0.567476534	0.059950711	-0.044445503	-0.58521563	0.696659838
k__Bacteria.Other.Other.Other	-0.527128017	-0.099761712	-0.482841219	0.112893198	0.903465346	0.631940025	-0.917202264	0.597183001	0.609270718
k__Bacteria.p__Verrucomicrobia.c__Verrucomicrobiae.o__Verrucomicrobiales	-0.202856503	-0.110929163	-0.043530277	-0.029674855	0.567476534	0.059950711	-0.044445503	-0.58521563	0.696659838
k__Bacteria.Other.Other.Other.Other	-0.527128017	-0.099761712	-0.482841219	0.112893198	0.903465346	0.631940025	-0.917202264	0.597183001	0.609270718
k__Bacteria.p__Bacteroidetes.c__Bacteroidia.o__Bacteroidales.f__.Barnesiellaceae.	0.815062641	0.445843681	-0.658607596	-0.190836949	-0.986176208	-0.096226482	0.621445326	0.930963948	-0.855643787
k__Bacteria.p__Verrucomicrobia.c__Verrucomicrobiae.o__Verrucomicrobiales.f__Verrucomicrobiaceae	-0.202856503	-0.110929163	-0.043530277	-0.029674855	0.567476534	0.059950711	-0.044445503	-0.58521563	0.696659838
k__Bacteria.Other.Other.Other.Other.Other	-0.527128017	-0.099761712	-0.482841219	0.112893198	0.903465346	0.631940025	-0.917202264	0.597183001	0.609270718
k__Bacteria.p__Bacteroidetes.c__Bacteroidia.o__Bacteroidales.f__.Barnesiellaceae..g__Barnesiella	0.815062641	0.445843681	-0.658607596	-0.190836949	-0.986176208	-0.096226482	0.621445326	0.930963948	-0.855643787
k__Bacteria.p__Firmicutes.c__Clostridia.o__Clostridiales.f__Lachnospiraceae.g__Clostridium	0.431672043	0.436793819	0.637050257	-0.441976681	0.979088351	-0.016099228	0.861279393	0.74648343	0.674939938
Dada_6.k__Bacteria.p__Firmicutes.c__Clostridia.o__Clostridiales.f__Lachnospiraceae.g__Ruminococcus.s__lactaris	-0.777781431	0.464164659	-0.672046531	-0.929724825	-0.118517686	-0.136366885	-0.216938603	0.866923359	0.846159967
k__Bacteria.p__Firmicutes.c__Clostridia.o__Clostridiales.f__Ruminococcaceae.g__Gemmiger	0.985927473	0.331982211	0.678492347	0.818638281	0.950773485	0.153465507	0.52543265	0.194822191	-0.274144004
k__Bacteria.p__Firmicutes.c__Clostridia.o__Clostridiales.f__Veillonellaceae.g__Succinispira	-0.191815343	-0.964827266	-0.811780828	0.929724825	-0.548695463	0.370547768	-0.293946603	-0.182951395	0.417160269

Supplementary Figure 6A: Time-point: T3_*Heatmap*

	Spearman's rho								
	tryptophan	n_acetyltrypto	indolelactic	indoleacetic	indolepropion	indoleacetami	indolealdehyd	x3_methylind	indoxyl_s
k__Bacteria.p__Actinobacteria	-0.18046294	-0.04631085	-0.02745098	-0.02353864	-0.21098039	0.030855599	-0.14823529	0.007858561	-0.02510789
k__Bacteria.p__Firmicutes	-0.14123187	0.043171128	0.014901961	0.094154578	-0.28784314	0.181177747	-0.04941176	-0.01886055	0.116123979
k__Bacteria.p__Firmicutes.c__Clostridia	0.259004922	0.045675849	0.062236378	-0.03154547	0.352672808	-0.22935762	0.191688044	-0.06485312	-0.14112448
k__Bacteria.p__Firmicutes.c__Clostridia.o__Clostridiales	0.249043195	0.039032089	0.078832745	-0.0282249	0.32611862	-0.22935762	0.214922958	-0.04822412	-0.1245216
k__Bacteria.p__Firmicutes.c__Clostridia.o__Clostridiales.f__Lachnospiraceae	0.142801109	-0.06043958	0.036862745	0.081600634	-0.11843137	0.113137196	-0.0054902	-0.00471514	0.073754419
k__Bacteria.p__Firmicutes.c__Clostridia.o__Clostridiales.f__Ruminococcaceae	-0.08003139	-0.02747254	-0.06039216	-0.0109847	0.12	-0.10047849	-0.04941176	-0.10844815	-0.05335426
k__Bacteria.p__Firmicutes.c__Clostridia.o__Clostridiales.f__Ruminococcaceae.Other	-0.1898784	-0.17660916	-0.19529412	0.010984701	0.138823529	-0.06724938	-0.15921569	-0.03614938	0.070615933
k__Bacteria.p__Bacteroidetes	0.032954102	0.07770803	-0.01647059	-0.19144764	0.016470588	-0.17326605	-0.03686275	-0.09115931	0.034523345
k__Bacteria.p__Actinobacteria.c__Coriobacteriia	-0.13024717	0.063579297	0.016470588	-0.03923107	-0.27058824	0.048261321	-0.12	0.023575684	0.050215775
k__Bacteria.p__Bacteroidetes.c__Bacteroidia	0.036092588	0.083987467	-0.01333333	-0.18517067	0.016470588	-0.17959541	-0.03372549	-0.09744616	0.028246373
k__Bacteria.p__Actinobacteria.c__Coriobacteriia.o__Coriobacteriales	-0.13024717	0.063579297	0.016470588	-0.03923107	-0.27058824	0.048261321	-0.12	0.023575684	0.050215775
k__Bacteria.p__Bacteroidetes.c__Bacteroidia.o__Bacteroidales	0.036092588	0.083987467	-0.01333333	-0.18517067	0.016470588	-0.17959541	-0.03372549	-0.09744616	0.028246373
k__Bacteria.p__Actinobacteria.c__Coriobacteriia.o__Coriobacteriales.f__Coriobacteriaceae	-0.13024717	0.063579297	0.016470588	-0.03923107	-0.27058824	0.048261321	-0.12	0.023575684	0.050215775
k__Bacteria.p__Firmicutes.c__Clostridia.o__Clostridiales.f__Lachnospiraceae.Other	-0.10357004	-0.15463113	-0.04	0.279325247	-0.04784314	0.057755352	-0.01019608	-0.00943027	0.224401743
k__Bacteria.p__Firmicutes.c__Clostridia.o__Clostridiales.Other	0.237515205	0.054288029	-0.02122647	-0.16358663	0.198899924	-0.12133472	0.109277033	0.045687282	-0.11639818
k__Bacteria.p__Firmicutes.c__Clostridia.o__Clostridiales.Other.Other	0.237515205	0.054288029	-0.02122647	-0.16358663	0.198899924	-0.12133472	0.109277033	0.045687282	-0.11639818
k__Bacteria.p__Actinobacteria.c__Actinobacteria	-0.09610143	-0.16312138	-0.12204819	-0.03623497	-0.06850447	0.005560031	-0.1535445	-0.01577912	-0.14809073
k__Bacteria.p__Firmicutes.c__Bacilli	-0.14285932	0.041058575	0.058383665	0.054460184	-0.32189913	0.177477808	-0.01893524	0.0118578	0.093924085
k__Bacteria.p__Bacteroidetes.c__Bacteroidia.o__Bacteroidales.f__Bacteroidaceae	0.149173547	0.027635579	-0.05049398	-0.17916611	0.02051318	-0.20851653	-0.01577937	-0.03241132	-0.10181687
k__Bacteria.p__Bacteroidetes.c__Bacteroidia.o__Bacteroidales.f__Bacteroidaceae.g__Bacteroides	0.149173547	0.027635579	-0.05049398	-0.17916611	0.02051318	-0.20851653	-0.01577937	-0.03241132	-0.10181687
k__Bacteria.p__Firmicutes.c__Clostridia.o__Clostridiales.f__Lachnospiraceae.g__Ruminococcus	0.095502641	0.104225613	0.118345267	-0.01815339	-0.02051318	0.127338337	0.075740971	0.02292508	0.093924085
k__Bacteria.p__Proteobacteria	-0.17010026	-0.06173503	0.085412197	0.157441636	-0.14235366	0.112485051	-0.14868049	0.046752128	0.291939516
k__Bacteria.p__Actinobacteria.c__Actinobacteria.o__Bifidobacteriales	-0.07537124	-0.17699355	-0.11340905	-0.04601613	-0.05630799	0.009600028	-0.14513186	-0.0238389	-0.14598219
k__Bacteria.p__Actinobacteria.c__Actinobacteria.o__Bifidobacteriales.f__Bifidobacteriaceae	-0.07537124	-0.17699355	-0.11340905	-0.04601613	-0.05630799	0.009600028	-0.14513186	-0.0238389	-0.14598219
k__Bacteria.p__Actinobacteria.c__Actinobacteria.o__Bifidobacteriales.f__Bifidobacteriaceae.g__Bifidobacterium	-0.07537124	-0.17699355	-0.11340905	-0.04601613	-0.05630799	0.009600028	-0.14513186	-0.0238389	-0.14598219
k__Bacteria.p__Firmicutes.c__Clostridia.o__Clostridiales.f__	0.135309583	0.164027764	0.172650956	-0.02387816	0.180607222	-0.03611614	0.191745993	0.122767442	0.058103527
k__Bacteria.p__Firmicutes.c__Clostridia.o__Clostridiales.f__.g__	0.135309583	0.164027764	0.172650956	-0.02387816	0.180607222	-0.03611614	0.191745993	0.122767442	0.058103527
k__Bacteria.p__Firmicutes.c__Erysipelotrichi	0.027959588	0.020778136	-0.10380917	0.002396536	0.076659077	0.074107086	0.025553026	0.103213212	-0.14938409
k__Bacteria.p__Firmicutes.c__Bacilli.o__Lactobacillales	-0.16056792	0.019179818	0.046314859	0.075890312	-0.35295117	0.237625982	-0.06547963	-0.0648083	0.136602561
k__Bacteria.p__Firmicutes.c__Erysipelotrichi.o__Erysipelotrichales	0.027959588	0.020778136	-0.10380917	0.002396536	0.076659077	0.074107086	0.025553026	0.103213212	-0.14938409
k__Bacteria.p__Bacteroidetes.c__Bacteroidia.o__Bacteroidales.f__Rikenellaceae	-0.10305105	0.119873863	-0.02395596	0.003994227	0.145332833	-0.17640709	0.012776513	-0.1336171	0.095062601
k__Bacteria.p__Firmicutes.c__Erysipelotrichi.o__Erysipelotrichales.f__Erysipelotrichaceae	0.027959588	0.020778136	-0.10380917	0.002396536	0.076659077	0.074107086	0.025553026	0.103213212	-0.14938409
k__Bacteria.p__Bacteroidetes.c__Bacteroidia.o__Bacteroidales.f__Rikenellaceae.g__Alistipes	-0.05512033	0.116677227	0.004791192	0.01038499	0.148526961	-0.13774252	0.038329538	-0.10161301	0.104648745
k__Bacteria.p__Firmicutes.Other	-0.11951479	-0.00160486	0.012828769	0.03930352	0.112251726	0.007279257	-0.05131507	-0.09078136	0.11470211
k__Bacteria.p__Firmicutes.Other.Other	-0.11951479	-0.00160486	0.012828769	0.03930352	0.112251726	0.007279257	-0.05131507	-0.09078136	0.11470211
k__Bacteria.p__Firmicutes.Other.Other.Other	-0.11951479	-0.00160486	0.012828769	0.03930352	0.112251726	0.007279257	-0.05131507	-0.09078136	0.11470211
k__Bacteria.p__Actinobacteria.c__Coriobacteriia.o__Coriobacteriales.f__Coriobacteriaceae.g__Eggerthella	-0.20774718	-0.0898719	-0.04971148	-0.06817958	-0.06895463	0.104336018	-0.04490069	-0.18557959	-0.00401056
k__Bacteria.p__Firmicutes.Other.Other.Other.Other	-0.11951479	-0.00160486	0.012828769	0.03930352	0.112251726	0.007279257	-0.05131507	-0.09078136	0.11470211
k__Bacteria.p__Firmicutes.c__Clostridia.o__Clostridiales.f__Ruminococcaceae.g__Clostridium	0.065951466	0.079824466	0.065111701	-0.15877205	0.001627793	-0.02791435	0.030928058	-0.06768624	0.018726959
k__Bacteria.p__Bacteroidetes.c__Bacteroidia.o__Bacteroidales.Other	-0.16954801	-0.00491636	0.01965003	-0.04668713	-0.09006264	0.096631205	-0.02456254	-0.1041859	0.197396477
k__Bacteria.p__Bacteroidetes.c__Bacteroidia.o__Bacteroidales.Other.Other	-0.16954801	-0.00491636	0.01965003	-0.04668713	-0.09006264	0.096631205	-0.02456254	-0.1041859	0.197396477
k__Bacteria.p__Firmicutes.c__Clostridia.o__Clostridiales.f__Ruminococcaceae.g__Faecalibacterium	0.273692173	0.103087218	0.076636712	-0.02308247	0.201892414	-0.18287576	0.119487347	0.097429204	-0.15250919
k__Bacteria.p__Proteobacteria.c__Deltaproteobacteria	-0.07139238	-0.02740551	0.067215288	0.023244031	-0.13526039	-0.01506729	-0.14355858	0.026606409	0.104598142
k__Bacteria.p__Proteobacteria.c__Deltaproteobacteria.o__Desulfovibrionales	-0.07139238	-0.02740551	0.067215288	0.023244031	-0.13526039	-0.01506729	-0.14355858	0.026606409	0.104598142
k__Bacteria.p__Proteobacteria.c__Deltaproteobacteria.o__Desulfovibrionales.f__Desulfovibrionaceae	-0.07139238	-0.02740551	0.067215288	0.023244031	-0.13526039	-0.01506729	-0.14355858	0.026606409	0.104598142
k__Bacteria.p__Firmicutes.c__Clostridia.o__Clostridiales.f__Lachnospiraceae.g__Dorea	0.092145982	0.057302428	0.055597831	0.046488063	-0.07717311	0.127234882	0.100408023	0.039078163	-0.01826317
k__Bacteria.p__Firmicutes.c__Clostridia.o__Clostridiales.f__Ruminococcaceae.g__	0.063090943	0.147823656	0.24479642	0.224138875	-0.02904364	0.098774448	0.103727296	-0.10226838	0.189272828
k__Bacteria.p__Firmicutes.c__Bacilli.o__Lactobacillales.f__Streptococcaceae	-0.15473572	-0.04016341	-0.07524731	0.091168615	-0.33108817	0.269884544	-0.14380597	-0.02010541	0.136334717
k__Bacteria.p__Firmicutes.c__Bacilli.o__Lactobacillales.f__Streptococcaceae.g__Streptococcus	-0.15473572	-0.04016341	-0.07524731	0.091168615	-0.33108817	0.269884544	-0.14380597	-0.02010541	0.136334717
k__Bacteria.p__Proteobacteria.c__Deltaproteobacteria.o__Desulfovibrionales.f__Desulfovibrionaceae.g__Desulfovibrio	-0.07778607	-0.06191859	0.038459737	0.007527684	-0.13210084	0.016024395	-0.133773	0.044399449	0.099532708
Dada_4.k__Bacteria.p__Actinobacteria.c__Coriobacteriia.o__Coriobacteriales.f__Coriobacteriaceae.g__Collinsella.s__	0.083354009	0.1029571	0.063766312	-0.00680441	-0.31713113	0.033448312	-0.08587197	0.109893747	0.019562676
k__Bacteria.p__Bacteroidetes.c__Bacteroidia.o__Bacteroidales.f__.Barnesiellaceae.	0.103767236	0.037438945	0.000850217	-0.03061984	0.106277187	-0.11664027	0.012753262	-0.07752195	0.077400151
k__Bacteria.p__Firmicutes.c__Clostridia.o__Clostridiales.f__Peptostreptococcaceae	0.021263778	-0.0170177	0.031458047	0.119077156	0.090973272	-0.2641559	0.126682407	-0.07241061	-0.28748628
k__Bacteria.p__Actinobacteria.c__Coriobacteriia.o__Coriobacteriales.f__Coriobacteriaceae.g__Collinsella	0.069745191	0.099553559	0.080770662	0.051033067	-0.32393287	0.006003543	-0.08587197	0.126931537	0.043378107
k__Bacteria.p__Bacteroidetes.c__Bacteroidia.o__Bacteroidales.f__.Barnesiellaceae..g__Barnesiella	0.103767236	0.037438945	0.000850217	-0.03061984	0.106277187	-0.11664027	0.012753262	-0.07752195	0.077400151
k__Bacteria.p__Firmicutes.c__Clostridia.o__Clostridiales.f__Lachnospiraceae.g__Clostridium	0.164156365	-0.13018542	-0.03825979	-0.09696283	0.072268487	0.036021259	0.041660657	-0.00937078	-0.22284439
k__Bacteria.p__Firmicutes.c__Clostridia.o__Clostridiales.f__Peptostreptococcaceae.g__Clostridium	0.026367084	-0.01871947	0.019555002	0.103767236	0.089272837	-0.2658712	0.118180232	-0.08433706	-0.28578517
Dada_1.k__Bacteria.p__Actinobacteria.c__Coriobacteriia.o__Coriobacteriales.f__Coriobacteriaceae.g__Collinsella.s__	0.106454045	0.170908661	0.131299035	0.017170007	-0.28061951	-0.03895491	0.004290818	0.150473972	0.014594506
Dada_6.k__Bacteria.p__Firmicutes.c__Clostridia.o__Clostridiales.f__Lachnospiraceae.g__Ruminococcus.s__lactaris	0.09615204	0.124531436	0.026603072	0.085850036	0.016305109	-0.11080507	0.004290818	0.091144234	0.042066518
k__Bacteria.p__Firmicutes.c__Clostridia.o__Clostridiales.f__Veillonellaceae	-0.03434001	-0.00772954	0.031752054	-0.01717001	-0.15704394	0.063193516	-0.09525616	-0.08082602	0.211191089
k__Bacteria.p__Actinobacteria.c__Coriobacteriia.o__Coriobacteriales.f__Coriobacteriaceae.Other	-0.16397357	-0.05925979	-0.17077456	0.133926056	0.050631654	-0.10387975	-0.04548267	0.004299256	-0.05236852
k__Bacteria.Other	0.039886219	-0.08761065	0.076274142	0.192494361	0.16641631	-0.02885281	0.123078729	0.053844281	0.176019618
k__Bacteria.Other.Other	0.039886219	-0.08761065	0.076274142	0.192494361	0.16641631	-0.02885281	0.123078729	0.053844281	0.176019618
k__Bacteria.Other.Other.Other	0.039886219	-0.08761065	0.076274142	0.192494361	0.16641631	-0.02885281	0.123078729	0.053844281	0.176019618
k__Bacteria.Other.Other.Other.Other	0.039886219	-0.08761065	0.076274142	0.192494361	0.16641631	-0.02885281	0.123078729	0.053844281	0.176019618
k__Bacteria.Other.Other.Other.Other.Other	0.039886219	-0.08761065	0.076274142	0.192494361	0.16641631	-0.02885281	0.123078729	0.053844281	0.176019618
k__Bacteria.p__Proteobacteria.c__Gammaproteobacteria	-0.11831059	-0.05611001	0.064826292	0.083255598	-0.08059485	0.104275198	-0.12089227	0.068464792	0.216464554
k__Bacteria.p__Proteobacteria.c__Gammaproteobacteria.o__Enterobacteriales	-0.11831059	-0.05611001	0.064826292	0.083255598	-0.08059485	0.104275198	-0.12089227	0.068464792	0.216464554
k__Bacteria.p__Bacteroidetes.c__Bacteroidia.o__Bacteroidales.f__Porphyromonadaceae	0.118310586	0.131507829	0.278577848	-0.04294236	-0.11388403	0.109577327	0.049057734	0.048276456	0.170893069
k__Bacteria.p__Proteobacteria.c__Gammaproteobacteria.o__Enterobacteriales.f__Enterobacteriaceae	-0.11831059	-0.05611001	0.064826292	0.083255598	-0.08059485	0.104275198	-0.12089227	0.068464792	0.216464554
k__Bacteria.p__Firmicutes.c__Clostridia.o__Clostridiales.f__Ruminococcaceae.g__Butyricicoccus	0.272552536	0.20778237	0.17170207	0.004381874	0.014016496	0.001767376	0.175206194	0.021943844	-0.05170611
k__Bacteria.p__Firmicutes.c__Clostridia.o__Clostridiales.f__Ruminococcaceae.g__Gemmiger	0.015774745	-0.00350688	-0.02978505	0.028043991	-0.0876031	0.146692228	-0.0140165	-0.24928206	-0.13583808
k__Bacteria.p__Proteobacteria.c__Gammaproteobacteria.o__Enterobacteriales.f__Enterobacteriaceae.Other	-0.11700555	-0.04877146	0.066454352	0.097504627	-0.10544091	0.090274085	-0.12138995	0.091443454	0.204759718
Dada_15.k__Bacteria.p__Firmicutes.c__Clostridia.o__Clostridiales.f__Lachnospiraceae.g__Ruminococcus.s__lactaris	-0.00897249	0.004488007	0.060092103	0.123820361	-0.11031834	0.016285259	-0.04215416	0.174340209	0.004486245
k__Bacteria.p__Firmicutes.c__Clostridia.o__Clostridiales.f__Lachnospiraceae.g__Roseburia	0.118436867	-0.11040496	-0.08341143	-0.02781472	0.070854868	-0.05247472	-0.06367969	0.035047774	-0.00269175
Dada_3.k__Bacteria.p__Firmicutes.c__Clostridia.o__Clostridiales.f__Lachnospiraceae.g__Ruminococcus.s__lactaris	0.082717019	-0.0181867	0.016355191	0.146345495	0.119938065	-0.06599259	-0.02180692	0.137471694	0.036359129
Dada_8.k__Bacteria.p__Firmicutes.c__Clostridia.o__Clostridiales.f__Lachnospiraceae.g__Ruminococcus.s__lactaris	-0.01566853	-0.16596722	-0.00368527	0.137330047	0.003685267	0.087360746	-0.055279	0.114467933	0.094011173
Dada_17.k__Bacteria.p__Actinobacteria.c__Actinobacteria.o__Bifidobacteriales.f__Bifidobacteriaceae.g__Bifidobacterium.s__breve	0.051613977	-0.09404809	-0.04606583	-0.1096797	-0.1437254	0.131041119	-0.0718627	0.037848268	-0.12626991
k__Bacteria.p__Verrucomicrobia	0.088481104	0.113410933	-0.01842633	-0.06359579	0.333516629	-0.05297407	0.112400632	0.118160447	-0.01382517
k__Bacteria.p__Verrucomicrobia.c__Verrucomicrobiae	0.088481104	0.113410933	-0.01842633	-0.06359579	0.333516629	-0.05297407	0.112400632	0.118160447	-0.01382517
k__Bacteria.p__Firmicutes.c__Bacilli.o__Bacillales	0.025806989	-0.13646194	-0.14741066	-0.03225874	0.147410665	-0.04739785	0.0055279	0.14677743	-0.24608807
k__Bacteria.p__Verrucomicrobia.c__Verrucomicrobiae.o__Verrucomicrobiales	0.088481104	0.113410933	-0.01842633	-0.06359579	0.333516629	-0.05297407	0.112400632	0.118160447	-0.01382517
k__Bacteria.p__Verrucomicrobia.c__Verrucomicrobiae.o__Verrucomicrobiales.f__Verrucomicrobiaceae	0.088481104	0.113410933	-0.01842633	-0.06359579	0.333516629	-0.05297407	0.112400632	0.118160447	-0.01382517
k__Bacteria.p__Bacteroidetes.c__Bacteroidia.o__Bacteroidales.f__Porphyromonadaceae.g__Macellibacteroides	0.137330047	0.129085615	0.248755497	-0.05622237	-0.09581693	0.113383095	0.029482133	0.034155754	0.135486691

Supplementary Figure 6B: Time-point: T3_*Merged Spearman’s rho and p-values matrix*

	Merge rho+P								
	tryptophan	n_acetyltryptc	indolelactic	indoleacetic	indolepropion	indoleacetami	indolealdehyd	x3_methylind	indoxyl_s
k__Bacteria.p__Actinobacteria	-0.06173835	-0.63176643	-0.77619781	-0.80748302	-0.02889803	0.751060766	-0.12475978	0.93525466	-0.794926
k__Bacteria.p__Firmicutes	-0.14372854	0.655054793	0.877355843	0.329710341	-0.00287448	0.062494085	-0.60886148	-0.84542017	0.229314367
k__Bacteria.p__Firmicutes.c__Clostridia	0.009739162	0.648609178	0.534359867	-0.75288747	0.000429817	-0.02300521	0.055645401	-0.51807337	-0.15899217
k__Bacteria.p__Firmicutes.c__Clostridia.o__Clostridiales	0.012935725	0.696971206	0.431245797	-0.77817827	0.001130138	-0.02300521	0.031890654	-0.63080401	-0.21395372
k__Bacteria.p__Firmicutes.c__Clostridia.o__Clostridiales.f__Lachnospiraceae	0.139329856	-0.53167476	0.702651318	0.398257238	-0.22002792	0.244741519	-0.95466032	-0.96112546	0.445157495
k__Bacteria.p__Firmicutes.c__Clostridia.o__Clostridiales.f__Ruminococcaceae	-0.40739144	-0.77618344	-0.53170183	-0.90946395	0.213979031	-0.30157267	-0.60886148	-0.26226656	-0.58072326
k__Bacteria.p__Firmicutes.c__Clostridia.o__Clostridiales.f__Ruminococcaceae.Other	-0.04934022	-0.06760689	-0.04313189	0.909463954	0.150538676	-0.48929999	-0.0991868	-0.70863992	0.464765035
k__Bacteria.p__Bacteroidetes	0.732995089	0.421309224	-0.86456469	-0.04749213	0.864564693	-0.07484222	-0.70265132	-0.34602273	0.720800552
k__Bacteria.p__Actinobacteria.c__Coriobacteriia	-0.17755178	0.510575076	0.864564693	-0.68465153	-0.00507612	0.619755138	-0.21397903	0.807458115	0.603176032
k__Bacteria.p__Bacteroidetes.c__Bacteroidia	0.708676608	0.384773881	-0.8901791	-0.05525	0.864564693	-0.0648189	-0.72689782	-0.31378087	0.76997374
k__Bacteria.p__Actinobacteria.c__Coriobacteriia.o__Coriobacteriales	-0.17755178	0.510575076	0.864564693	-0.68465153	-0.00507612	0.619755138	-0.21397903	0.807458115	0.603176032
k__Bacteria.p__Bacteroidetes.c__Bacteroidia.o__Bacteroidales	0.708676608	0.384773881	-0.8901791	-0.05525	0.864564693	-0.0648189	-0.72689782	-0.31378087	0.76997374
k__Bacteria.p__Actinobacteria.c__Coriobacteriia.o__Coriobacteriales.f__Coriobacteriaceae	-0.17755178	0.510575076	0.864564693	-0.68465153	-0.00507612	0.619755138	-0.21397903	0.807458115	0.603176032
k__Bacteria.p__Firmicutes.c__Clostridia.o__Clostridiales.f__Lachnospiraceae.Other	-0.28364493	-0.1095584	-0.67870365	0.003832825	-0.62027907	0.552639189	-0.91590866	-0.92234314	0.020177594
k__Bacteria.p__Firmicutes.c__Clostridia.o__Clostridiales.Other	0.014139887	0.575050014	-0.82636774	-0.09103588	0.03983005	-0.21316363	0.258766988	0.637426239	-0.22918128
k__Bacteria.p__Firmicutes.c__Clostridia.o__Clostridiales.Other.Other	0.014139887	0.575050014	-0.82636774	-0.09103588	0.03983005	-0.21316363	0.258766988	0.637426239	-0.22918128
k__Bacteria.p__Actinobacteria.c__Actinobacteria	-0.32144762	-0.09250429	-0.20780092	-0.70852371	-0.47955333	0.954567688	-0.11303392	-0.8708647	-0.12654584
k__Bacteria.p__Firmicutes.c__Bacilli	-0.14114402	0.672457334	0.547436345	0.574814313	-0.00091001	0.069423551	-0.84530253	0.902925069	0.333304485
k__Bacteria.p__Bacteroidetes.c__Bacteroidia.o__Bacteroidales.f__Bacteroidaceae	0.124395025	0.775979705	-0.60284892	-0.06496081	0.832594563	-0.03290921	-0.87083634	-0.7388499	-0.29427935
k__Bacteria.p__Bacteroidetes.c__Bacteroidia.o__Bacteroidales.f__Bacteroidaceae.g__Bacteroides	0.124395025	0.775979705	-0.60284892	-0.06496081	0.832594563	-0.03290921	-0.87083634	-0.7388499	-0.29427935
k__Bacteria.p__Firmicutes.c__Clostridia.o__Clostridiales.f__Lachnospiraceae.g__Ruminococcus	0.325243635	0.283178984	0.222665137	-0.85166706	-0.83259456	0.19267345	0.435118979	0.813585483	0.333304485
k__Bacteria.p__Proteobacteria	-0.08030977	-0.52575311	0.379680908	0.105502896	-0.14315552	0.25071321	-0.12621151	0.631233892	0.002685772
k__Bacteria.p__Actinobacteria.c__Actinobacteria.o__Bifidobacteriales	-0.43934287	-0.06947986	-0.24442915	-0.63683968	-0.56331558	0.922073235	-0.13633518	-0.80705059	-0.13419393
k__Bacteria.p__Actinobacteria.c__Actinobacteria.o__Bifidobacteriales.f__Bifidobacteriaceae	-0.43934287	-0.06947986	-0.24442915	-0.63683968	-0.56331558	0.922073235	-0.13633518	-0.80705059	-0.13419393
k__Bacteria.p__Actinobacteria.c__Actinobacteria.o__Bifidobacteriales.f__Bifidobacteriaceae.g__Bifidobacterium	-0.43934287	-0.06947986	-0.24442915	-0.63683968	-0.56331558	0.922073235	-0.13633518	-0.80705059	-0.13419393
k__Bacteria.p__Firmicutes.c__Clostridia.o__Clostridiales.f__	0.166043332	0.093272071	0.077078812	-0.80690785	0.064405472	-0.71350132	0.049597368	0.209538295	0.552011849
k__Bacteria.p__Firmicutes.c__Clostridia.o__Clostridiales.f__.g__	0.166043332	0.093272071	0.077078812	-0.80690785	0.064405472	-0.71350132	0.049597368	0.209538295	0.552011849
k__Bacteria.p__Firmicutes.c__Erysipelotrichi	0.775287176	0.83205458	-0.28902263	0.980478901	0.433646352	0.452379071	0.794100929	0.292670622	-0.12720826
k__Bacteria.p__Firmicutes.c__Bacilli.o__Lactobacillales	-0.10113201	0.844802425	0.636182712	0.438436462	-0.00031226	0.015971956	-0.50363295	-0.50878252	0.163104774
k__Bacteria.p__Firmicutes.c__Erysipelotrichi.o__Erysipelotrichales	0.775287176	0.83205458	-0.28902263	0.980478901	0.433646352	0.452379071	0.794100929	0.292670622	-0.12720826
k__Bacteria.p__Bacteroidetes.c__Bacteroidia.o__Bacteroidales.f__Rikenellaceae	-0.29273258	0.221154183	-0.80670634	0.967470604	0.137709361	-0.07364883	0.896175174	-0.17311732	0.331755337
k__Bacteria.p__Firmicutes.c__Erysipelotrichi.o__Erysipelotrichales.f__Erysipelotrichaceae	0.775287176	0.83205458	-0.28902263	0.980478901	0.433646352	0.452379071	0.794100929	0.292670622	-0.12720826
k__Bacteria.p__Bacteroidetes.c__Bacteroidia.o__Bacteroidales.f__Rikenellaceae.g__Alistipes	-0.57358759	0.233716759	0.960970736	0.915558379	0.129266931	-0.16249612	0.695439795	-0.30021772	0.28531382
k__Bacteria.p__Firmicutes.Other	-0.22364638	-0.98696745	0.896037557	0.689020239	0.252893586	0.941321027	-0.60120115	-0.35601059	0.242852377
k__Bacteria.p__Firmicutes.Other.Other	-0.22364638	-0.98696745	0.896037557	0.689020239	0.252893586	0.941321027	-0.60120115	-0.35601059	0.242852377
k__Bacteria.p__Firmicutes.Other.Other.Other	-0.22364638	-0.98696745	0.896037557	0.689020239	0.252893586	0.941321027	-0.60120115	-0.35601059	0.242852377
k__Bacteria.p__Actinobacteria.c__Coriobacteriia.o__Coriobacteriales.f__Coriobacteriaceae.g__Eggerthella	-0.03440748	-0.36032928	-0.6126177	-0.48755772	-0.48246519	0.291392845	-0.64742688	-0.05918354	-0.96742727
k__Bacteria.p__Firmicutes.Other.Other.Other.Other	-0.22364638	-0.98696745	0.896037557	0.689020239	0.252893586	0.941321027	-0.60120115	-0.35601059	0.242852377
k__Bacteria.p__Firmicutes.c__Clostridia.o__Clostridiales.f__Ruminococcaceae.g__Clostridium	0.505869769	0.420857188	0.511149644	-0.10924272	0.986894365	-0.77974228	0.754965969	-0.49536875	0.850165394
k__Bacteria.p__Bacteroidetes.c__Bacteroidia.o__Bacteroidales.Other	-0.08829443	-0.96059551	0.843347962	-0.63882293	-0.36508107	0.334659804	-0.80489577	-0.29562377	0.047208556
k__Bacteria.p__Bacteroidetes.c__Bacteroidia.o__Bacteroidales.Other.Other	-0.08829443	-0.96059551	0.843347962	-0.63882293	-0.36508107	0.334659804	-0.80489577	-0.29562377	0.047208556
k__Bacteria.p__Firmicutes.c__Clostridia.o__Clostridiales.f__Ruminococcaceae.g__Faecalibacterium	0.006113326	0.301942865	0.442507467	-0.81714182	0.043059141	-0.06885957	0.23116388	0.329779154	-0.12658177
k__Bacteria.p__Proteobacteria.c__Deltaproteobacteria	-0.476143	-0.7845326	0.502173651	0.816551087	-0.17687744	-0.88128217	-0.15177766	0.79088918	0.296520435
k__Bacteria.p__Proteobacteria.c__Deltaproteobacteria.o__Desulfovibrionales	-0.476143	-0.7845326	0.502173651	0.816551087	-0.17687744	-0.88128217	-0.15177766	0.79088918	0.296520435
k__Bacteria.p__Proteobacteria.c__Deltaproteobacteria.o__Desulfovibrionales.f__Desulfovibrionaceae	-0.476143	-0.7845326	0.502173651	0.816551087	-0.17687744	-0.88128217	-0.15177766	0.79088918	0.296520435
k__Bacteria.p__Firmicutes.c__Clostridia.o__Clostridiales.f__Lachnospiraceae.g__Dorea	0.357756965	0.567531783	0.578836138	0.642671865	-0.44100816	0.207262931	0.316117636	0.696944708	-0.85536889
k__Bacteria.p__Firmicutes.c__Clostridia.o__Clostridiales.f__Ruminococcaceae.g__	0.528911431	0.140267569	0.01452408	0.025287611	-0.77183914	0.327563599	0.300385342	-0.30811091	0.058891052
k__Bacteria.p__Firmicutes.c__Bacilli.o__Lactobacillales.f__Streptococcaceae	-0.12394089	-0.68976415	-0.45421418	0.364706894	-0.00099126	0.007702085	-0.15263364	-0.84178889	0.175261162
k__Bacteria.p__Firmicutes.c__Bacilli.o__Lactobacillales.f__Streptococcaceae.g__Streptococcus	-0.12394089	-0.68976415	-0.45421418	0.364706894	-0.00099126	0.007702085	-0.15263364	-0.84178889	0.175261162
k__Bacteria.p__Proteobacteria.c__Deltaproteobacteria.o__Desulfovibrionales.f__Desulfovibrionaceae.g__Desulfovibrio	-0.43929852	-0.53829388	0.702076013	0.940339578	-0.18888942	0.874278765	-0.18335264	0.65936059	0.32237377
Dada_4.k__Bacteria.p__Actinobacteria.c__Coriobacteriia.o__Coriobacteriales.f__Coriobacteriaceae.g__Collinsella.s__	0.410973808	0.310023641	0.529217199	-0.9464887	-0.00175266	0.743172336	-0.39681423	0.279079925	0.846989605
k__Bacteria.p__Bacteroidetes.c__Bacteroidia.o__Bacteroidales.f__.Barnesiellaceae.	0.306056553	0.712013231	0.993306344	-0.76263245	0.294330471	-0.25320774	0.899858353	-0.4451382	0.445192733
k__Bacteria.p__Firmicutes.c__Clostridia.o__Clostridiales.f__Peptostreptococcaceae	0.833870536	-0.86674291	0.756252206	0.240177001	0.369366471	-0.00966369	0.21129462	-0.47572093	-0.00457274
k__Bacteria.p__Actinobacteria.c__Coriobacteriia.o__Coriobacteriales.f__Coriobacteriaceae.g__Collinsella	0.49148585	0.326290535	0.42545773	0.614699793	-0.00139181	0.953103509	-0.39681423	0.211226241	0.66874593
k__Bacteria.p__Bacteroidetes.c__Bacteroidia.o__Bacteroidales.f__.Barnesiellaceae..g__Barnesiella	0.306056553	0.712013231	0.993306344	-0.76263245	0.294330471	-0.25320774	0.899858353	-0.4451382	0.445192733
k__Bacteria.p__Firmicutes.c__Clostridia.o__Clostridiales.f__Lachnospiraceae.g__Clostridium	0.10540564	-0.19926522	-0.70578657	-0.33886271	0.475787008	0.724193768	0.681015149	-0.92646231	-0.02794368
k__Bacteria.p__Firmicutes.c__Clostridia.o__Clostridiales.f__Peptostreptococcaceae.g__Clostridium	0.794803832	-0.85356098	0.84699413	0.306056553	0.378381697	-0.00920247	0.243565945	-0.40615993	-0.00481875
Dada_1.k__Bacteria.p__Actinobacteria.c__Coriobacteriia.o__Coriobacteriales.f__Coriobacteriaceae.g__Collinsella.s__	0.295689469	0.093295423	0.196964733	0.866060295	-0.00582296	-0.70392431	0.966367202	0.139945903	0.88600185
Dada_6.k__Bacteria.p__Firmicutes.c__Clostridia.o__Clostridiales.f__Lachnospiraceae.g__Ruminococcus.s__lactaris	0.344902471	0.221384001	0.793766554	0.39904722	0.872702406	-0.27971056	0.966367202	0.371305974	0.679438991
k__Bacteria.p__Firmicutes.c__Clostridia.o__Clostridiales.f__Veillonellaceae	-0.73586842	-0.93949753	0.755024837	-0.8660603	-0.12277323	0.53757079	-0.34924131	-0.42788338	0.038025734
k__Bacteria.p__Actinobacteria.c__Coriobacteriia.o__Coriobacteriales.f__Coriobacteriaceae.Other	-0.10723444	-0.5606267	-0.09331488	0.188313182	0.618803771	-0.31086476	-0.65491225	0.96636221	-0.60695301
k__Bacteria.Other	0.696411551	-0.39161029	0.455432849	0.05970176	0.103422364	-0.77925089	0.228435245	0.598928517	0.085099784
k__Bacteria.Other.Other	0.696411551	-0.39161029	0.455432849	0.05970176	0.103422364	-0.77925089	0.228435245	0.598928517	0.085099784
k__Bacteria.Other.Other.Other	0.696411551	-0.39161029	0.455432849	0.05970176	0.103422364	-0.77925089	0.228435245	0.598928517	0.085099784
k__Bacteria.Other.Other.Other.Other	0.696411551	-0.39161029	0.455432849	0.05970176	0.103422364	-0.77925089	0.228435245	0.598928517	0.085099784
k__Bacteria.Other.Other.Other.Other.Other	0.696411551	-0.39161029	0.455432849	0.05970176	0.103422364	-0.77925089	0.228435245	0.598928517	0.085099784
k__Bacteria.p__Proteobacteria.c__Gammaproteobacteria	-0.24916513	-0.58483965	0.527611504	0.417403707	-0.43227629	0.3131333	-0.2388151	0.505476878	0.034994002
k__Bacteria.p__Proteobacteria.c__Gammaproteobacteria.o__Enterobacteriales	-0.24916513	-0.58483965	0.527611504	0.417403707	-0.43227629	0.3131333	-0.2388151	0.505476878	0.034994002
k__Bacteria.p__Bacteroidetes.c__Bacteroidia.o__Bacteroidales.f__Porphyromonadaceae	0.249165135	0.200383709	0.006639297	-0.67574892	-0.26714279	0.289163943	0.632642976	0.638681683	0.096002915
k__Bacteria.p__Proteobacteria.c__Gammaproteobacteria.o__Enterobacteriales.f__Enterobacteriaceae	-0.24916513	-0.58483965	0.527611504	0.417403707	-0.43227629	0.3131333	-0.2388151	0.505476878	0.034994002
k__Bacteria.p__Firmicutes.c__Clostridia.o__Clostridiales.f__Ruminococcaceae.g__Butyricicoccus	0.007936931	0.043060217	0.094320298	0.965956045	0.891367268	0.986359959	0.087788763	0.830992709	-0.61451961
k__Bacteria.p__Firmicutes.c__Clostridia.o__Clostridiales.f__Ruminococcaceae.g__Gemmiger	0.877885231	-0.97276106	-0.77164759	0.784732811	-0.39333244	0.155906206	-0.89136727	-0.01532744	-0.18580131
k__Bacteria.p__Proteobacteria.c__Gammaproteobacteria.o__Enterobacteriales.f__Enterobacteriaceae.Other	-0.25649183	-0.63634552	0.519115312	0.344351807	-0.30633648	0.384606303	-0.23892828	0.375871093	0.047059597
Dada_15.k__Bacteria.p__Firmicutes.c__Clostridia.o__Clostridiales.f__Lachnospiraceae.g__Ruminococcus.s__lactaris	-0.93096395	0.965448605	0.56163248	0.231882751	-0.2866297	0.875911902	-0.68389107	0.092790941	0.965449596
k__Bacteria.p__Firmicutes.c__Clostridia.o__Clostridiales.f__Lachnospiraceae.g__Roseburia	0.252812901	-0.28660204	-0.42044155	-0.78826867	0.493739049	-0.61485052	-0.53850831	0.735435543	-0.97926561
Dada_3.k__Bacteria.p__Firmicutes.c__Clostridia.o__Clostridiales.f__Lachnospiraceae.g__Ruminococcus.s__lactaris	0.42655923	-0.86127549	0.875034639	0.159515317	0.248791638	-0.52872556	-0.83391043	0.187008438	0.726720305
Dada_8.k__Bacteria.p__Firmicutes.c__Clostridia.o__Clostridiales.f__Lachnospiraceae.g__Ruminococcus.s__lactaris	-0.88082061	-0.11239299	-0.97185932	0.18881801	0.971859323	0.406448925	-0.59670394	0.274076379	0.368346927
Dada_17.k__Bacteria.p__Actinobacteria.c__Actinobacteria.o__Bifidobacteriales.f__Bifidobacteriaceae.g__Bifidobacterium.s__breve	0.621388219	-0.36833343	-0.65924537	-0.2939466	-0.1688896	0.213044126	-0.4915219	0.717625332	-0.22695052
k__Bacteria.p__Verrucomicrobia	0.39718577	0.278005306	-0.85999376	-0.54283241	0.00141039	-0.61468711	0.281958163	0.258895879	-0.89475467
k__Bacteria.p__Verrucomicrobia.c__Verrucomicrobiae	0.39718577	0.278005306	-0.85999376	-0.54283241	0.00141039	-0.61468711	0.281958163	0.258895879	-0.89475467
k__Bacteria.p__Firmicutes.c__Bacilli.o__Bacillales	0.804953174	-0.1917903	-0.15822783	-0.75756694	0.158227833	-0.65241816	0.957799924	0.160785256	-0.01853429
k__Bacteria.p__Verrucomicrobia.c__Verrucomicrobiae.o__Verrucomicrobiales	0.39718577	0.278005306	-0.85999376	-0.54283241	0.00141039	-0.61468711	0.281958163	0.258895879	-0.89475467
k__Bacteria.p__Verrucomicrobia.c__Verrucomicrobiae.o__Verrucomicrobiales.f__Verrucomicrobiaceae	0.39718577	0.278005306	-0.85999376	-0.54283241	0.00141039	-0.61468711	0.281958163	0.258895879	-0.89475467
k__Bacteria.p__Bacteroidetes.c__Bacteroidia.o__Bacteroidales.f__Porphyromonadaceae.g__Macellibacteroides	0.18881801	0.216925647	0.017258282	-0.59059043	-0.35904466	0.281283595	0.777781431	0.744157367	0.194822191
